# Potential Immune-Related Adverse Events Associated With Monotherapy and Combination Therapy of Ipilimumab, Nivolumab, and Pembrolizumab for Advanced Melanoma: A Systematic Review and Meta-Analysis

**DOI:** 10.3389/fonc.2020.00091

**Published:** 2020-02-11

**Authors:** Abdulaali R. Almutairi, Ali McBride, Marion Slack, Brian L. Erstad, Ivo Abraham

**Affiliations:** ^1^Center for Health Outcomes and PharmacoEconomic Research, College of Pharmacy, University of Arizona, Tucson, AZ, United States; ^2^Department of Pharmacy Practice and Science, College of Pharmacy, University of Arizona, Tucson, AZ, United States; ^3^University of Arizona Cancer Center, Tucson, AZ, United States

**Keywords:** immune checkpoint inhibitors, ipilimumab, nivolumab, pembrolizumab, incidence, immune-related adverse events, advanced, melanoma

## Abstract

**Background:** The use of ipilimumab, nivolumab, and pembrolizumab as monotherapies or in combination has transformed the management of advanced melanoma even though these drugs are associated with a new profile of immune-related adverse events (irAEs). The incidence of irAEs from clinical trials of these agents is an important factor for clinicians when treating patients with advanced melanoma. In the current study, we aimed to profile the incidence of potential irAEs of these agents when used as monotherapy and as combination therapy.

**Methods:** We searched the Medline, Embase, and Cochrane databases; clinicaltrials.gov; and websites of regulatory agencies in the USA, Europe, Australia, and Japan for phase 1–3 trials of ipilimumab, nivolumab, and pembrolizumab for advanced melanoma. Random effect meta-analysis was utilized to profile the incidence of potential irAEs.

**Results:** A total of 58 reports of 35 trials including 6,331 patients with advanced melanoma and reporting irAE data were included in the meta-analyses. We found higher incidences of potential irAEs in combination therapies vs. monotherapies for most of the types of irAEs. Among the monotherapies, ipilimumab users had the most frequent incidence of potential irAEs related to the gastrointestinal system (diarrhea, 29%; and colitis, 8%) and skin (rash, 31%; pruritus, 27%; and dermatitis, 10%), with hypophysitis in 4% of the patients. The most frequent potential irAEs among nivolumab users were maculopapular rash (13%), erythema (4%), hepatitis (3%), and infusion-related reactions (3%), while they were arthralgia (12%), hypothyroidism (8%), and hyperglycemia (6%), among pembrolizumab users.

**Conclusion:** Especially the combination therapies tend to elevate the incidence of potential irAEs. Clinicians should be vigilant about irAEs following combination therapy as well as gastrointestinal and skin irAEs following ipilimumab therapy, in addition to being aware of potential irAEs leading to hyperglycemia, thyroid, hepatic, and musculoskeletal disorders following nivolumab and pembrolizumab therapy.

## Background

Melanoma is a type of skin cancer, with increasing in incidence over the past several years. It is estimated to be the fifth most common cancer in the USA in 2019 and was the 21st most common cancer worldwide in 2018 (1, 2). Surgery is the standard primary treatment for melanoma. However, pharmacotherapy choices for patients with advanced melanoma have greatly expanded over the last few years, including the use of immune checkpoints inhibitors (ICIs) that target the cytotoxic T lymphocyte antigen-4 (CTLA-4) and programmed death-1 (PD-1) pathways (3). The US Food and Drug Administration (FDA) approved ipilimumab as the first ICI (anti-CTLA-4) therapy for advanced melanoma in 2011, followed by the anti-PD-1 drugs nivolumab and pembrolizumab in 2014 (4–6). The combined use of anti-CTLA-4 and anti-PD-1 drugs provides better clinical benefits over monotherapies, leading to the FDA-approved combination regimen of nivolumab and ipilimumab in 2015 (7).

The clinical benefits of ICIs are associated with a spectrum of adverse events (AEs) owing to the activation of the immune system that can affect healthy tissues of the body organs. These immune-related adverse events (irAEs) require close monitoring, use of corticosteroids and infliximab, holding the ICIs, or discontinuation of the drugs in case of severe irAEs such as diarrhea and colitis (8–11). The reported incidence of irAEs is higher after anti-CTLA-4 treatment (90%) than after anti-PD-1 treatment (70%) across several types of advanced cancer (11), and the rates may vary based on the cancer type (12–16).

The incidences of irAEs owing to anti-CTLA-4 or anti-PD-1 monotherapy as well as those owing to concomitant or sequential combination of these drugs are not well-estimated for advanced melanoma. Therefore, the aim of systematic review and meta-analysis was to profile the incidence of potential irAEs associated with mono- and combination therapies of ipilimumab, nivolumab, and pembrolizumab in advanced melanoma.

## Methods

### Search Strategy

To identify eligible trials, we performed a comprehensive search of Medline, Embase, and Cochrane library from inception until October 30, 2018 (Table S1). We further searched clinicaltrials.gov as well as the websites of regulatory bodies in the USA (FDA), Europe [the European Medicines Agency (EMA)], Australia [Therapeutic Goods Administration (TGA)], and Japan [Pharmaceuticals and Medical Devices Agency (PMDA)]. In addition, we screened the references of published reviews, meta-analyses, and relevant trials to include any related citation. We have reported this systemic review and meta-analysis following the Cochrane recommendations for the preferred reporting items for systematic reviews and meta-analyses (PRISMA) (17).

### Inclusion and Exclusion Criteria

We included phase 1–3 clinical trials reporting any treatment-related AEs that could potentially be classified as irAEs following the use of anti-CTLA-4 or anti-PD-1 as monotherapy or in any concomitant or sequential combination of these agents in patients with advanced melanoma. We excluded trials reported in a non-English language, those restricted to uveal melanoma or pediatric populations, and those in the settings of compassionate care and expanded access programs or adjuvant therapy. We selected the most recently updated safety data for each included trial.

### Outcome Measures

The primary outcome of interest was the incidence of potential irAEs across all regimens and doses studied in the trials. We obtained these events across all included trials if an AE was reported in any trial as “irAE,” “AE of special interest,” “selected treatment-related AE of special interest,” or listed in the clinical practice guidelines for managing toxicities related to immunotherapies published by the European Society for Medical Oncology (ESMO), the American Society of Clinical Oncology (ASCO), and the National Comprehensive Cancer Network (NCCN) (8–10). We used the highest incidence of a potential irAE to estimate the overall incidence of irAEs if the information was not given in the included trials. The secondary outcome was the incidence of potential irAEs for the FDA-approved doses of anti-CTLA-4 and anti-PD-1 therapies.

### Selection of Studies and Data Extraction

We used the citation management software Endnote® (version X7.3; Clarivate Analytics, Philadelphia, PA, USA) in the selection process. One reviewer (A.A.) removed the duplicates, after which, two reviewers (A.A. and A.M.) independently performed the selection in two stages: (1) screening of the title and abstract of each citation; and (2) reviewing the full text of the retained articles. During each stage, disagreements were resolved by consensus or escalated to a third reviewer (I.A.). One reviewer (A.A.) extracted the data into a Microsoft Excel file, including the trial title, trial phase, the total number of patients, and the number of patients who experienced AEs (all grades and grade ≥3). A second reviewer (A.M.) verified the extraction file.

### Assessment of the Quality of the Included Trials

To assess the risk of bias in the included trials, we used the Cochrane Collaboration risk of bias assessment tool for randomized trials and the Newcastle-Ottawa Scale for non-randomized trials (18, 19). Two investigators (A.A. and A.M.) performed the quality assessment of the trials and resolved any disagreement by internal discussion or escalation to a third investigator (I.A.).

### Statistical Analysis

We performed random effect meta-analyses of binomial data to estimate the incidence of potential irAEs. We employed the Freeman-Tukey double arcsine transformation to stabilize the variance and avoid overestimation bias that might be introduced by continuity correction for zero events (20). The 95% confidence interval (95%CI) was estimated by using the score test owing to its better performance than the Wald and Exact CIs (21). We assessed heterogeneity based on the *I*^2^ statistic and conducted subgroup analyses when heterogeneity of *I*^2^ >75% was observed. We evaluated publication bias using the funnel plot and Egger's test. Meta-analyses were performed using R software version 3.4.3 (“meta” package).

## Results

### Search Results

**Figure 1:**
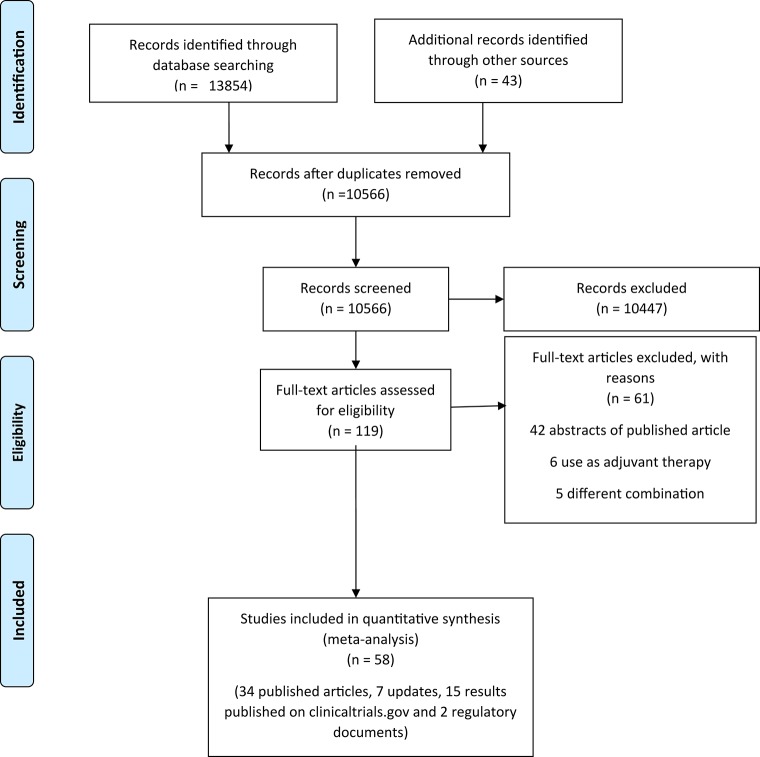
Preferred reporting items for systematic reviews and meta-analyses (PRISMA) flow diagram.

We initially identified 13,897 records through database searching and additional sources. After removing the duplicates, screening the titles and abstracts, and applying the inclusion and exclusion criteria, we retained 58 records (34 published articles, 7 updates, 15 results published on clinicaltrials.gov, and 2 regulatory documents) of 35 unique trials (Figure 1) (22–62).^1^^,^^2^^,^^3^^,^^4^^,^^5^^,^^6^^,^^7^^,^^8^^,^^9^^,^^10^^,^^11^^,^^12^^,^^13^^,^^14^^,^^15^^,^^16^^,^^17^

### Characteristics of the Included Trials

The 35 trials included 6,331 advanced melanoma patients treated with anti-CTLA-4 or anti-PD-1 monotherapy or combination therapy: 2,726 received ipilimumab in 21 studies (23, 29, 39–53, 56)^12^, (61, 62), 1,019 received nivolumab in 7 studies (23, 24, 34, 36–38, 60), 1,632 received pembrolizumab in 5 studies (28–30, 33, 57), 630 received the concomitant combination of nivolumab and ipilimumab in 5 studies (23–25, 54, 56), 103 patients received the sequential combination of ipilimumab followed by nivolumab in 2 studies including 33 patients previously treated with ipilimumab then used nivolumab therapy every 2 weeks for up to 48 doses within 4–12 weeks from the ipilimumab treatment, and 70 patients treated with ipilimumab every 3 weeks for 4 doses followed 3 weeks later by nivolumab therapy every 2 weeks up to progression, unacceptable toxicity or withdrawal from the study (22, 54), 68 patients received the sequential combination of nivolumab followed by ipilimumab in 1 study in which patients treated with nivolumab every 2 weeks for 6 doses then followed 2 weeks later by ipilimumab therapy every 3 weeks for 4 doses, and subsequently followed by nivolumab therapy every 2 weeks up to progression, unacceptable toxicity or withdrawal from study (22), and 153 received the combination of pembrolizumab and ipilimumab in 1 study (26). Approximately 50% of the patients included in these trials had received prior systemic therapy before starting the assigned ICI therapy. Of the trials included, 11 were phase 1, 18 phase 2, 2 phase 1/2, and 6 phase 3 studies (Table 1).

**Table 1:** Characteristics of included studies.

**Trial**	**Phase**	**Treatment arm**	**Control arm**	**Treatment (N)**	**Previous treatment (N)**
**Pembrolizumab and Ipilimumab**					
KEYNOTE-029	1	Pembrolizumab 2 mg/kgQ3wks (up to 2 years) + ipilimumab 1 mg/kgQ3wks for 4 doses	NA	153	20 prior systemic therapy
**Pembrolizumab**					
KEYNOTE-001	1	Pembrolizumab 2 mg/kgQ3wks	NA	89	342 prior ipilimumab therapy 342 prior systemic therapy
		Pembrolizumab 10 mg/kgQ3wks		172	
		Pembrolizumab 10 mg/kgQ2wks		81	
		Pembrolizumab 2 mg/kgQ3wks		73	0 prior ipilimumab therapy 152 prior systemic therapy
		Pembrolizumab 10 mg/kgQ3wks		141	
		Pembrolizumab 10 mg/kgQ2wks		99	
KEYNOTE-041	1	Pembrolizumab 2 mg/kgQ3wks	NA	42	0 prior ipilimumab therapy 30 prior systemic therapy
NCT02085070	2	Pembrolizumab 10 mg/kgQ2wks	NA	23	13 prior ipilimumab therapy 16 prior systemic therapy
KEYNOTE-002	2	Pembrolizumab 2 mg/kgQ3wks	chemotherapy	178	178 prior ipilimumab therapy 178 prior systemic therapy
		Pembrolizumab 10 mg/kgQ3wks		179	179 prior ipilimumab therapy 178 prior systemic therapy
KEYNOTE-006	3	Pembrolizumab 10 mg/kgQ2wks	ipilimumab 3 mg/kgQ3wks for 4 doses	278	0 prior ipilimumab therapy 96 prior systemic therapy
		Pembrolizumab 10 mg/kgQ3wks		277	0 prior ipilimumab therapy 91 prior systemic therapy
**Nivolumab and Ipilimumab (concomitant)**					
CA209–004	1	Nivolumab 0.3 mg/kg+ ipilimumab 3 mg/kgQ3wks for 4 doses then nivolumab 0.3 mg/kgQ3wks for 4 doses then nivolumab 0.3 mg/kg+ ipilimumab 3 mg/kgQ12wks for 8 doses	NA	14	0 prior ipilimumab therapy 42 prior systemic therapy
		Nivolumab 1 mg/kg+ ipilimumab 3 mg/kgQ3wks for 4 doses then nivolumab 1 mg/kgQ3wks for 4 doses then nivolumab 1 mg/kg+ ipilimumab 3 mg/kgQ12wks for 8 doses		17	
		Nivolumab 3 mg/kg+ ipilimumab 1 mg/kgQ3wks for 4 doses then nivolumab 3 mg/kgQ3wks for 4 doses then nivolumab 3 mg/kg+ ipilimumab 1 mg/kgQ12wks for 8 doses		16	
		Nivolumab 3 mg/kg+ ipilimumab 3 mg/kgQ3wks for 4 doses then nivolumab 3 mg/kgQ3wks for 4 doses then nivolumab 3 mg/kg+ ipilimumab 3 mg/kgQ12wks for 8 doses		6	
		Nivolumab 1 mg/kg+ ipilimumab 3 mg/kgQ3wks for 4 doses then nivolumab 1 mg/kgQ3wks for up to 48 doses		41	
CheckMate-069	2	Nivolumab 1 mg/kg+ ipilimumab 3 mg/kgQ3wks for 4 doses then nivolumab 3 mg/kgQ2wks	Ipilimumab 3 mg/kgQ3wks for 4 doses	94	0 prior systemic therapy
CheckMate-204	2	Nivolumab 1 mg/kg+ ipilimumab 3 mg/kgQ3wks for 4 doses then nivolumab 3 mg/kgQ2wks	NA	94	16 prior systemic therapy
NCT02374242	2	Nivolumab 1 mg/kg+ ipilimumab 3 mg/kgQ3wks for 4 doses then nivolumab 3 mg/kgQ2wks	Nivolumab 3 mg/kgQ2wks	35	8 prior systemic therapy
CheckMate-067	3	Nivolumab 1 mg/kg+ ipilimumab 3 mg/kgQ3wks for 4 doses then nivolumab 3 mg/kgQ2wks	Nivolumab 3 mg/kgQ2wks OR ipilimumab 3 mg/kgQ3wks for 4 doses	313	0 prior systemic therapy
**Nivolumab and Ipilimumab (Sequential of ipilimumab then nivolumab)**					
CA209–004	1	Ipilimumab followed by nivolumab 1 mg/kg(within 4–12wks of last dose of ipilimumab	NA	17	33 prior ipilimumab therapy 33 prior systemic therapy
		Ipilimumab followed by nivolumab 3 mg/kg(within 4–12wks of last dose of ipilimumab		16	
CA209–064	2	Induction period 1 (ipilimumab 3 mg/kgQ3wks up to 4 doses during weeks 1–13). Induction period 2 (nivolumab 3 mg /kg Q2wks up to 6 doses during weeks 13–25) Continuation period (nivolumab 3 mg/kgQ2wks from week 25 till completion of 2 years)	Nivolumab followed by ipilimumab	70	0 prior systemic therapy
**Nivolumab and Ipilimumab (Sequential of nivolumab then ipilimumab)**					
CA209–064	2	Induction period 1 (nivolumab 3 mg/kgQ2wks up to 6 doses during weeks 1–13). Induction period 2 (ipilimumab 3 mg /kg Q3wks up to 4 doses during weeks 13–25) Continuation period (nivolumab 3 mg/kgQ2wks from week 25 till completion of 2 years)	Ipilimumab followed by nivolumab	68	0 prior systemic therapy
**Nivolumab**					
CA209–003	1	Nivolumab 3 mg/kgQ2wks	NA	107	69 prior immunotherapy 107 prior systemic therapy
CA209–006	1/2	Nivolumab 3 mg/kgQ2wks for 24 weeks then Q12wks for up to 2 years	NA	61	61 prior ipilimumab therapy 31 prior systemic therapy
JapicCTI-142533	2	Nivolumab 3 mg/kgQ2wks	NA	23	0 prior systemic therapy
NCT02374242	2	Nivolumab 3 mg/kgQ2wks	Nivolumab 1 mg/kg+ Ipilimumab 3 mg/kgQ3wks for 4 doses then Nivolumab 3 mg/kgQ2wks	41	18 prior systemic therapy
CheckMate 066	3	Nivolumab 3 mg/kgQ2wks	Dacarbazine	206	33 prior systemic therapy
CheckMate 067	3	Nivolumab 3 mg/kgQ2wks	Nivolumab +ipilimumab OR ipilimumab 3 mg/kgQ3wks for 4 doses	313	0 prior systemic therapy
CheckMate 037	3	Nivolumab 3 mg/kgQ2wks	Investigator's choice of chemotherapy.	268	268 prior ipilimumab therapy
**Ipilimumab**					
Downey et. al	1	Ipilimumab 3 mg/kg can be escalated to 5 mg/kg then to 9 mg/kg if no objective response	NA	66	57 prior systemic therapy
Maker et.al	1	Ipilimumab 3 mg/kg can be escalated to 5 mg/kg then to 9 mg/kg if no objective response	NA	46	29 prior chemotherapy 2 prior hormonal therapy 38 prior immunotherapy,
CA184–078	1	Ipilimumab 10 mg/kgQ3wks for 4 doses + placebo	Ipilimumab + dacarbazine OR ipilimumab + paclitaxel+carboplatin	20	0 prior systemic therapy
CA184–087	1	Ipilimumab 10 mg/kgQ3wks for 4 doses	NA	75	
CA184–013	2	Ipilimumab 3 mg/kgQ3wks for 4 doses	Ipilimumab + dacarbazine	39	0 prior chemotherapy 19 prior immunotherapy
CA184–169	3	Ipilimumab 3 mg/kgQ3wks for 4 doses	NA	362	205 prior systemic therapy
		Ipilimumab 10 mg/kgQ3wks for 4 doses		364	206 prior systemic therapy
CA184–396	2	Ipilimumab 3 mg/kgQ3wks for 4 doses	NA	20	16 prior systemic therapy
CheckMate 067	3	Ipilimumab 3 mg/kgQ3wks for 4 doses	Nivolumab +ipilimumab OR nivolumab 3 mg/kgQ2wks	311	0 prior systemic therapy
CheckMate-069	2	Ipilimumab 3 mg/kgQ3wks for 4 doses	Nivolumab 1 mg/kg+ ipilimumab 3 mg/kgQ3wks for 4 doses then nivolumab 3 mg/kgQ2wks	46	0 prior systemic therapy
KEYNOTE-006	3	Ipilimumab 3 mg/kgQ3wks for 4 doses	Pembrolizumab 10 mg/kgQ2wks	256	0 prior ipilimumab therapy 97 prior systemic therapy
			pembrolizumab 10 mg/kgQ3wks		
CA184–001 (MDX010–15)	1/2	Ipilimumab 3 mg/kg or 2.8 or 5 mg/kg on Days 1, 57, and 85	NA	34	31 prior systemic therapy
		Single dose of ipilimumab 7.5, 10, 15, or 20 mg/kg		30	21 prior systemic therapy
		Ipilimumab 10 mg/kgQ3wks for 4 doses		24	18 prior systemic therapy
NCT01134614	2	Ipilimumab 10 mg/kgQ3wks for 4 doses then Q12wks	Ipilimumab + sargramostim	120	54 prior systemic therapy
NCT01216696	2	Ipilimumab 10 mg/kgQ3wks for 4 doses then Q12wks	NA	25	19 prior systemic therapy
NCT01355120	2	Ipilimumab 3 mg/kgQ3wks for 4 doses	NA	103	103 prior systemic therapy
NCT01740297	2	Ipilimumab 3 mg/kgQ3wks for 4 doses	Ipilimumab+ talimogene laherparepvec	95	29 prior anticancer therapy
CA184–004	2	Ipilimumab 3 mg/kgQ3wks for 4 doses then Q12	NA	40	29 prior systemic therapy
		Ipilimumab 10 mg/kgQ3wks for 4 doses then Q12wks		42	33 prior systemic therapy
CA184–007	2	Ipilimumab 10 mg/kgQ3wks for 4 doses + placebo	Ipilimumab + budesonide	57	41 prior systemic therapy
CA184–008	2	Ipilimumab 10 mg/kgQ3wks for 4 doses then Q12wks	NA	155	155 prior systemic therapy
CA184–022	2	Ipilimumab 0.3 mg/kgQ3wks for 4 doses then Q12wks	NA	72	72 prior systemic therapy
		Ipilimumab 3 mg/kgQ3wks for 4 doses then Q12wks		71	71 prior systemic therapy
		Ipilimumab 10 mg/kgQ3wks for 4 doses then Q12wks		71	71 prior systemic therapy
MDX010–20	3	Ipilimumab 10 mg/kgQ3wks for 4 doses then Q12wks if patients met the re-induction criteria	Ipilimumab + gp100 or gp100 alone	131	131 prior systemic therapy
CA184–042	2	Ipilimumab 10 mg/kgQ3wks for 4 doses then Q12wks	Ipilimumab+ corticosteroids	51	40 prior systemic therapy

*N, number of patients*.

### Quality of the Included Trials and Publication Bias

Our quality assessment of the 19 randomized trials revealed that 10 trials were not blinded (Figure 2). All the non-randomized trials (16 trials) were open-label, and most of them were single arm (Table S2). Open-label trials are susceptible to detection bias and may lead to over-reporting of irAEs. Results from funnel plots and the Egger's test did not show evidence of publication bias (Figures S1–S16).

**Figure 2:**
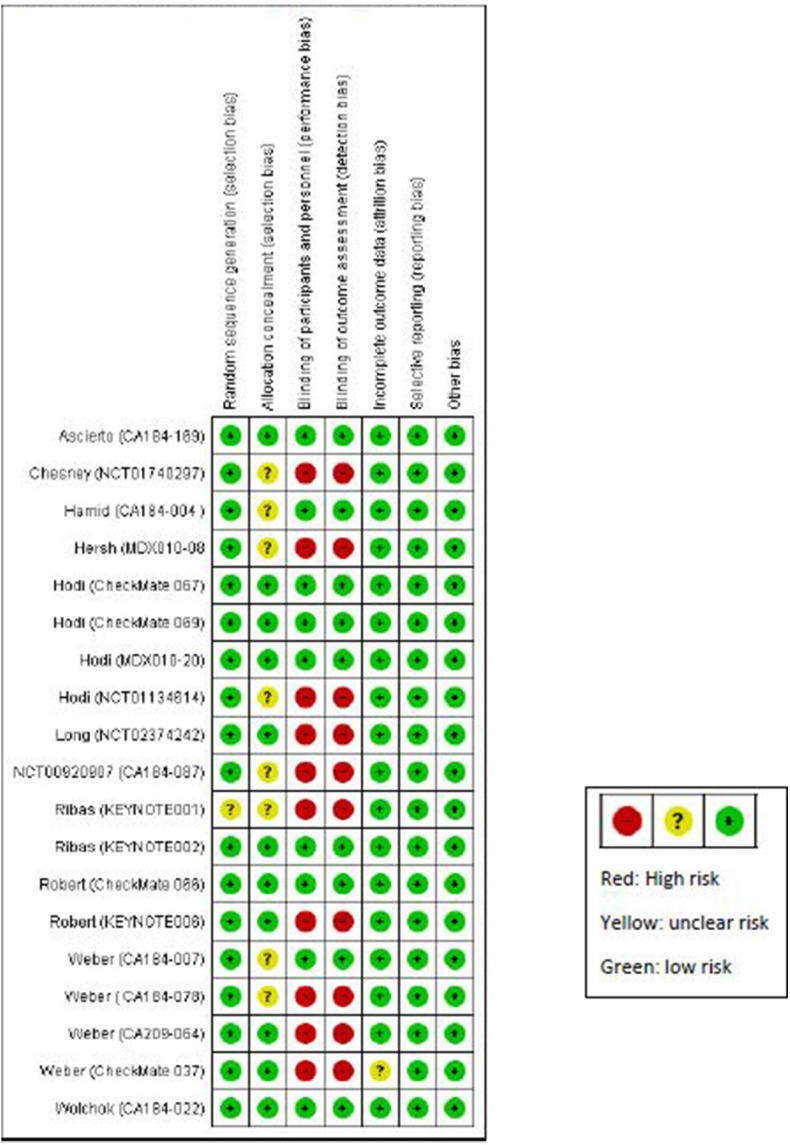
Quality assessment of randomized studies using Cochrane Collaboration risk of bias assessment tool for randomized trials.

### Incidence of Potential irAEs

The incidence of all grade and grade ≥3 potential irAEs owing to all the doses or FDA-approved doses of ICIs based on system disorders is summarized in Tables 2–5.

**Table 2:** Incidence of All-grades Potential Immune-Related Adverse Events (irAEs) in Advanced Melanoma presented as % with 95% confidence interval, and *I*^2^ for the % of heterogeneity (*I*^2^ not reported if the outcome was obtained from one study).

**irAEs**	**Ipilimumab**	**Nivolumab**	**Pembrolizumab**	**Pembrolizumab +Ipilimumab**	**Nivolumab +Ipilimumab**	**Ipilimumab then Nivolumab (Sequential)**	**Nivolumab then Ipilimumab (Sequential)**
Number of studies	21	7	5	1	5	2	1
Overall incidence	62.22 [54.59; 69.58], 93.0%	49.39 [36.17; 62.67], 94.0%	2300 [17.00; 29.00], 81.0%	60.13 [52.22; 67.55]	83.00 [65.00; 95.00], 96.0%	35.00 [17.00; 55.00], 73.9%	47.00 [36.00; 59.00]
**Blood and lymphatic system disorders**							
Lymphopenia	0.98 [0.00; 3.00], 14.3%	4.01 [0.87; 9.02], 66.6%	4.76 [1.32; 15.79]	NR	NR	NR	NR
**Cardiac disorders**							
Pericarditis	0.14 [0.02; 0.78]	0.37 [0.07; 2.08]	0.15 [0.03; 0.86]	NR	NR	NR	NR
Ventricular arrhythmia	0.00 [0.00; 7.71]	0.37 [0.07; 2.08]	NR	NR	1.06 [0.19; 5.78]	NR	NR
**Endocrine disorders**							
Acute adrenocortical insufficiency	0.14 [0.00; 0.53], 0.0%	0.00 [0.00; 1.21]	0.28 [0.05; 1.57]	0.65 [0.12; 3.61]	0.32 [0.06; 1.79]	NR	NR
Adrenal insufficiency	0.67 [0.28; 1.17], 0.00%	1.68 [0.16; 4.34], 68.1%	0.33 [0.03; 0.82], 0.0%	3.27 [1.40; 7.42]	4.21 [2.67; 6.04], 0.0%	3.76 [0.61; 8.71], 0.0%	8.82 [4.11; 17.94]
Adrenocorticotropic hormone deficiency	0.41 [0.14; 1.21]	NR	NR	0.65 [0.12; 3.61]	NR	NR	NR
Autoimmune thyroiditis	0.00 [0.00; 0.16], 0.0%	0.32 [0.06; 1.79]	NR	NR	0.96 [0.01; 2.90], 35.8%	NR	NR
Hyperthyroidism	0.90 [0.14; 2.16], 68.2%	3.01 [1.96; 4.24], 0.0%	3.34 [1.60; 5.61], 73.5%	11.11 [7.05; 17.07]	10.16 [5.94; 15.28], 58.1%	0.00 [0.00; 10.43]	NR
Hypophysitis	4.13 [2.35; 6.31], 74.5%	0.31 [0.00; 1.14], 0.0%	0.66 [0.05; 1.70], 61.2%	10.46 [6.54; 16.31]	10.40 [6.60; 14.88], 56.9%	3.76 [0.61; 8.71], 0.0%	5.88 [2.31; 14.17]
Hypopituitarism	1.46 [0.69; 2.45], 25.7%	0.16 [0.00; 0.88], 4.2%	0.36 [0.10; 0.75], 0.0%	0.65 [0.12; 3.61]	1.25 [0.18; 2.95], 0.0%	NR	NR
Hypothyroidism	2.84 [1.46; 4.57], 64.8%	7.02 [4.37; 10.19], 60.3%	8.34 [7.01; 9.77], 0.0%	16.34 [11.32; 23.01]	16.39 [13.50; 19.49], 1.7%	11.22 [0.03; 33.98], 85.7%	22.06 [13.85; 33.26]
Lymphocytic hypophysitis	0.06 [0.00; 0.39], 0.0%	0.00 [0.00; 01.21]	NR	0.65 [0.12; 3.61]	0.64 [0.18; 2.30]	NR	NR
Thyroiditis	0.65 [0.25; 1.20], 0.0%	1.63 [0.00; 4.96], 42.9%	0.75 [0.13; 1.80], 62.8%	5.23 [2.67; 9.98]	5.83 [1.51; 12.30], 72.2%	0.00 [0.00; 10.43]	NR
Thyrotoxic crisis	0.14 [0.02; 0.78]	NR	NR	NR	NR	NR	NR
**Eye disorders**							
Uveitis	0.93 [0.03; 2.56], 73.2%	0.24 [0.00; 1.33], 0.0%	0.70 [0.28; 1.24], 0.0%	2.61 [1.02; 6.53]	2.03 [0.19; 5.17], 55.3%	0.00 [0.00; 10.43]	NR
**Gastrointestinal disorders**							
Autoimmune colitis	1.78 [0.27; 4.20], 68.0%	0.64 [0.18; 2.30]	0.28 [0.05; 1.57]	1.96 [0.67; 5.61]	1.16 [0.10; 2.97], 38.6%	1.43 [0.25; 7.66]	0.00 [0.00; 5.35]
Autoimmune pancreatitis	0.00 [0.00; 0.01], 0.0%	0.32 [0.06; 1.79]	0.18 [0.03; 1.01]	1.31 [0.36; 4.64]	0.17 [0.00; 2.18], 62.6%	0.00 [0.00; 5.20]	0.00 [0.00; 5.35]
Colitis	7.98 [6.44; 9.66], 42.9%	1.80 [0.42; 3.86], 63.4%	2.05 [1.13; 3.20], 39.3%	9.15 [5.53; 14.77]	13.35 [9.52; 17.68], 46.1%	7.80 [0.00; 42.08], 93.7%	16.18 [9.28; 26.69]
Diarrhea	29.21 [24.27; 34.40], 85.6%	18.18 [13.11; 23.85], 76.2%	14.09 [10.54; 18.02], 68.8%	26.14 [19.83; 33.63]	40.46 [34.70; 46.36], 46.4%	25.22 [0.96; 64.29], 93.2%	47.06 [35.67; 58.76]
Enterocolitis	1.44 [0.00; 4.68], 87.0%	NR	0.00 [0.00; 0.21], 0.0%	NR	0.44 [0.00; 1.39], 0.0%	1.43 [0.25; 7.66]	0.00 [0.00; 5.35]
Frequent bowel movements	0.39 [0.07; 2.18]	0.97 [0.27; 3.47]	0.54 [0.18; 1.58]	0.65 [0.12; 3.61]	NR	NR	NR
Gastrointestinal perforation	0.54 [0.11; 1.20], 31.8%	0.10 [0.00; 0.72], 16.3%	0.15 [0.00; 1.11], 70.1%	NR	0.63 [0.02; 1.79], 0.0%	NR	NR
Gastrointestinal toxicity	NR	8.70 [2.42; 26.80]	NR	NR	NR	NR	NR
Pancreatitis	0.09 [0.00; 0.42], 0.0%	0.62 [0.07; 1.50], 0.0%	0.23 [0.03; 0.57], 0.0%	3.27 [1.40; 7.42]	1.35 [0.43; 2.65], 1.5%	1.45 [0.00; 5.25], 0.0%	0.00 [0.00; 5.35]
Peritonitis	0.04 [0.00; 0.41], 0.0%	0.32 [0.06; 1.79]	NR	NR	0.32 [0.06; 1.79]	NR	NR
Rectal hemorrhage	0.64 [0.00; 2.06], 62.8%	NR	NR	NR	0.36 [0.00; 1.36], 0.0%	NR	NR
Ulcerative colitis	0.28 [0.08; 1.00]	NR	NR	NR	NR	NR	NR
**General disorders**							
Infusion-related reaction	0.44 [0.00; 1.40], 33.0%	2.96 [1.24; 5.23], 59%	0.28 [0.05; 1.57]	3.92 [1.81; 8.29]	2.75 [1.19; 4.82], 0.0%	2.20 [0.00; 9.57], 56.1%	7.35 [3.18; 16.09]
**Hepatobiliary disorders**							
Acute hepatic failure	0.19 [0.00; 1.13], 38.6%	NR	NR	NR	NR	NR	NR
Acute hepatitis	0.06 [0.00; 0.39], 0.0%	NR	NR	NR	0.32 [0.06; 1.79]	NR	NR
Autoimmune hepatitis	0.53 [0.05; 1.33], 35.0%	0.64 [0.18; 2.30]	0.51 [0.12; 1.07], 0.0%	NR	1.62 [0.52; 3.18], 0.0%	NR	NR
Hepatitis	0.35 [0.02; 0.96], 21.8%	3.01 [0.00; 24.87], 94.2%	0.92 [0.48; 1.48], 2.1%	9.80 [6.03; 15.55]	4.85 [0.44; 12.65], 87.3%	0.00 [0.00; 5.20]	4.41 [1.51; 12.19]
Hepatocellular injury	0.51 [0.13; 1.06], 0.0%	0.80 [0.14; 1.88], 18.8%	0.00 [0.00; 0.69]	NR	0.89 [0.11; 2.17], 0.0%	NR	NR
Hepatotoxicity	0.51 [0.16; 1.02], 0.0%	0.78 [0.00; 7.85], 65.1%	0.00 [0.00; 0.69]	NR	3.19 [1.74; 5.78]	0.00 [0.00; 5.20]	1.47 [0.26; 7.87]
Hyperbilirubinemia	0.35 [0.00; 1.59], 59.1%	0.32 [0.06; 1.79]	0.00 [0.00; 1.06]	NR	1.85 [0.68; 3.50], 0.0%	1.43 [0.25; 7.66]	0.00 [0.00; 5.35]
**Immune system disorders**							
Episcleritis	1.21 [0.00; 8.48], 68.5%	NR	NR	NR	NR	NR	NR
Hypersensitivity	0.81 [0.18; 1.74], 37.5%	2.00 [1.09; 3.12], 0.0%	0.87 [0.34; 1.61], 0.0%	0.65 [0.12; 3.61]	2.88 [1.52; 5.37]	NR	NR
**Infections**							
Aseptic meningitis	0.00 [0.00; 0.94], 31.6%	0.00 [0.00; 1.21]	NR	0.65 [0.12; 3.61]	0.32 [0.06; 1.79]	0.00 [0.00; 5.20]	1.47 [0.26; 7.87]
Conjunctivitis	1.71 [0.41; 3.60], 52.2%	NR	0.72 [0.28; 1.84]	0.65 [0.12; 3.61]	NR	NR	NR
Encephalitis	0.10 [0.00; 0.42], 0.0%	NR	0.00 [0.00; 0.69]	NR	0.32 [0.06; 1.79]	0.00 [0.00; 5.20]	1.47 [0.26; 7.87]
**Investigations**							
Alanine aminotransferase (increased)	3.92 [2.50; 5.60], 63.3%	3.02 [1.83; 4.46], 19%	3.84 [1.56; 6.97], 79.0%	11.76 [7.57; 17.83]	22.80 [15.99; 30.40], 74%	12.30 [0.00; 38.78], 88.6%	35.29 [25.00; 47.16]
Amylase (increased)	1.24 [0.00; 4.18], 84.6%	5.81 [3.49; 8.62], 0.0%	NR	16.34 [11.32; 23.01]	11.10 [8.00; 14.61], 31.4%	9.62 [0.37; 26.70], 78.1%	25.00 [16.24; 36.44]
Aspartate aminotransferase (increased)	4.28 [2.70; 6.14], 61.5%	3.13 [1.80; 4.76], 31.3%	4.91 [2.33; 8.27], 78.3%	11.11 [7.05; 17.07]	22.15 [15.20; 29.95], 75.7%	8.27 [0.00; 44.54], 94.1%	30.88 [21.17; 42.64]
Blood alkaline phosphatase (increased)	2.68 [0.98; 4.99], 78.6%	3.81 [0.62; 9.04], 83.2%	2.39 [0.19; 6.56], 89.6%	3.92 [1.81; 8.29]	5.32 [3.27; 7.76]	10.00 [4.93; 19.23]	16.18 [9.28; 26.69]
Blood bilirubin (increased)	0.65 [0.00; 2.09], 69.9%	0.27 [0.00; 1.09], 0.0%	1.63 [0.80; 2.73], 21.6%	0.65 [0.12; 3.61]	2.55 [0.86; 4.92], 34.6%	1.43 [0.25; 7.66]	11.76 [6.08; 21.53]
Blood corticotropin (decreased)	0.47 [0.00; 4.00], 71.5%	NR	0.18 [0.03; 1.01]	NR	NR	NR	NR
Blood creatinine (increased)	0.60 [0.02; 1.66], 63.9%	0.89 [0.15; 2.03], 32.2%	0.99 [0.00; 3.22], 78.2%	2.61 [1.02; 6.53]	3.44 [1.94; 5.27], 0.0%	0.00 [0.00; 1.73], 0.0%	1.47 [0.26; 7.87]
Blood thyroid stimulating hormone (abnormal)	1.68 [0.03; 4.83], 57.2%	2.75 [0.01; 8.82], 81.8%	2.34 [1.12; 3.95], 24.6%	3.27 [1.40; 7.42]	6.66 [0.04; 20.47], 82.8%	2.32 [0.00; 10.59], 61.0%	2.94 [0.81; 10.10]
Blood urea (increased)	NR	NR	NR	NR	1.06 [0.19; 5.78]	NR	NR
Gamma-glutamyl transferase (increased)	0.75 [0.27; 1.38], 0.0%	0.55 [0.00; 3.93], 52.1%	NR	7.19 [4.06; 12.41]	3.29 [1.68; 5.30], 0.0%	0.00 [0.00; 10.43]	NR
Hepatic enzyme (increased)	0.19 [0.00; 0.75], 31.6%	0.34 [0.00; 1.07], 0.0%	0.28 [0.05; 1.57]	0.65 [0.12; 3.61]	1.60 [0.68; 3.68]	0.00 [0.00; 10.43]	1.47 [0.26; 7.87]
Lipase (increased)	2.75 [0.56; 6.12], 86.2%	3.10 [0.00; 11.74], 85.4%	NR	20.92 [15.22; 28.03]	15.78 [12.50; 19.35], 19.2%	21.25 [6.77; 40.55], 75.2%	32.35 [22.44; 44.16]
Liver function test (abnormal)	0.00 [0.00; 0.23], 0.0%	0.64 [0.07; 1.63], 17.8%	0.18 [0.03; 1.01]	0.65 [0.12; 3.61]	0.36 [0.00; 1.36], 0.0%	NR	NR
Thyroxine free (decreased)	NR	1.87 [0.51; 6.56]	2.31 [0.90; 5.79]	1.31 [0.36; 4.64]	NR	NR	NR
Thyroxine (increased)	NR	NR	0.58 [0.10; 3.20]	NR	NR	NR	NR
Transaminases (increased)	0.68 [0.22; 1.32], 0.0%	0.64 [0.18; 2.30]	1.29 [0.00; 4.39], 3.8%	0.65 [0.12; 3.61]	2.48 [1.29; 3.98], 0.0%	NR	NR
**Metabolism and nutrition disorders**							
Diabetes mellitus	0.07 [0.00; 4.52], 57.7%	0.44 [0.00; 1.35], 0.0%	0.09 [0.00; 0.69], 37.5%	0.65 [0.12; 3.61]	0.96 [0.33; 2.78]	NR	NR
Diabetic ketoacidosis	0.00 [0.00; 0.36], 35.8%	0.11 [0.00; 0.94], 35.4%	0.18 [0.03; 1.01]	0.65 [0.12; 3.61]	0.36 [0.00; 1.36], 0.0%	NR	NR
Hyperglycemia	4.42 [0.62; 10.65], 91.8%	0.58 [0.10; 1.31], 0.0%	5.86 [3.65; 8.50], 0.0%	1.31 [0.36; 4.64]	2.60 [1.19; 4.46], 0.0%	2.86 [0.79; 9.83]	7.35 [3.18; 16.09]
Hyperlipasemia	0.14 [0.02; 0.78]	NR	NR	NR	NR	NR	NR
Type 1 diabetes mellitus	0.00 [0.00; 1.48]	NR	0.09 [0.00; 0.69], 37.5%	1.96 [0.67; 5.61]	1.06 [0.19; 5.78]	NR	NR
**Musculoskeletal and connective tissue disorders**							
Arthralgia	6.32 [3.64; 9.60], 84.6%	9.13 [5.81; 13.04], 68%	12.16 [8.54; 16.29], 74.1%	13.07 [8.62; 19.33]	14.82 [12.10; 17.77], 0.0%	9.60 [4.40; 16.31], 0.0%	22.06 [13.85; 33.26]
Arthritis	0.20 [0.00; 1.39], 65.7%	0.10 [0.00; 0.72], 16.3%	1.21 [0.19; 2.83], 48.3%	0.65 [0.12; 3.61]	0.32 [0.06; 1.79]	0.00 [0.00; 5.20]	5.88 [2.31; 14.17]
Arthropathy	0.32 [0.06; 1.80]	0.32 [0.06; 1.79]	0.58 [0.10; 3.20]	NR	0.64 [0.18; 2.30]	NR	NR
Hand-foot-syndrome (Palmar-plantar erythrodysesthesia syndrome)	0.97 [0.17; 5.30]	NR	NR	NR	NR	NR	NR
Joint swelling	NR	NR	0.58 [0.10; 3.20]	0.65 [0.12; 3.61]	NR	NR	NR
Muscle spasms	0.51 [0.02; 1.39], 18.1%	3.35 [0.84; 7.32], 78.7%	0.59 [0.01; 1.73], 64.4%	1.96 [0.67; 5.61]	0.64 [0.18; 2.30]	0.00 [0.00; 5.20]	2.94 [0.81; 10.10]
Myalgia	3.23 [2.02; 4.65], 31.2%	3.68 [2.28; 5.36], 6.5%	5.94 [3.25; 9.36], 84.7%	6.54 [3.59; 11.61]	11.90 [5.22; 20.66], 82.7%	5.71 [2.24; 13.79]	19.12 [11.53; 30.01]
Myopathy	NR	NR	0.15 [0.03; 0.86]	0.65 [0.12; 3.61]	NR	NR	NR
Polymyalgia rheumatica	0.28 [0.08; 1.00]	NR	NR	0.65 [0.12; 3.61]	NR	NR	NR
Polymyositis/ myositis	0.10 [0.00; 0.75], 19.7%	0.32 [0.06; 1.79]	0.40 [0.10; 0.87], 0.0%	0.65 [0.12; 3.61]	2.13 [0.59; 7.43]	NR	NR
Rhabdomyolysis	0.00 [0.00; 3.89]	NR	0.15 [0.03; 0.86]	NR	1.06 [0.19; 5.78]	NR	NR
**Nervous system disorders**							
Guillain-Barre syndrome	0.01 [0.00; 0.23], 0.0%	0.11 [0.00; 0.94], 35.4%	0.18 [0.03; 1.01]	NR	0.36 [0.00; 1.36], 0.0%	NR	NR
Lethargy	0.20 [0.00; 1.25], 57.0%	0.00 [0.00; 1.21]	1.12 [0.01; 3.53], 71.9%	5.88 [3.13; 10.80]	0.32 [0.06; 1.79]	NR	NR
Myasthenia gravis	1.33 [0.24; 7.17]	NR	NR	NR	NR	0.00 [0.00; 5.20]	1.47 [0.26; 7.87]
Myelitis	0.39 [0.07; 2.18]	NR	0.28 [0.05; 1.57]	NR	NR	NR	NR
Neuropathy	0.63 [0.00; 10.45], 71.7%	NR	NR	0.65 [0.12; 3.61]	0.64 [0.18; 2.30]	0.00 [0.00; 5.20]	1.47 [0.26; 7.87]
Peripheral neuropathy	1.75 [0.30; 3.98], 78.4%	2.36 [0.84; 4.47], 38.5%	0.94 [0.03; 2.63], 69.7%	2.61 [1.02; 6.53]	3.53 [1.15; 6.89], 40.4%	NR	NR
**Renal and urinary disorders**							
Acute renal failure	0.13 [0.00; 0.59]. 21.5%	0.04 [0.00; 0.44], 0.0%	0.78 [0.30; 1.44], 0.0%	NR	1.27 [0.37; 2.57], 0.0%	0.00 [0.00; 1.73], 0.0%	1.47 [0.26; 7.87]
Nephritis	0.21 [0.00; 0.60], 0.0%	0.12 [0.00; 0.76], 0.0%	0.16 [0.00; 0.57], 0.0%	1.31 [0.36; 4.64]	1.00 [0.00; 4.20], 62.6%	0.00 [0.00; 10.43]	NR
Nephrotoxicity	NR	0.00 [0.00; 14.31]	NR	NR	0.32 [0.06; 1.79]	NR	NR
Renal failure	0.45 [0.00; 2.34], 70.2%	0.82 [0.27; 1.60], 0.0%	0.74 [0.20; 1.53], 28.6%	NR	0.32 [0.06; 1.79]	0.67 [0.00; 3.87], 0.0%	1.47 [0.26; 7.87]
**Respiratory, thoracic and mediastinal disorders**							
Alveolitis	1.52 [0.27; 8.10]	NR	0.00 [0.00; 1.06]	NR	NR	NR	NR
Lung infiltration	0.25 [0.00; 4.18], 71.1%	1.87 [0.51; 6.56]	NR	NR	0.32 [0.06; 1.79]	NR	NR
Pneumonitis	0.69 [0.17; 1.46], 33.1%	1.42 [0.68; 2.35], 0.0%	1.96 [1.24; 2.80], 0.0%	10.46 [6.54; 16.31]	7.98 [5.91; 10.30], 0.0%	2.78 [0.19; 7.31], 0.0%	11.76 [6.08; 21.53]
Pulmonary toxicity	NR	4.35 [0.77; 20.99]	NR	NR	NR	NR	NR
**Skin and subcutaneous tissue disorders**							
Alopecia	1.86 [0.67; 3.45], 1.8%	1.95 [0.01; 6.02], 77%	1.31 [0.46; 2.56], 44.8%	1.96 [0.67; 5.61]	2.26 [0.00; 9.78], 61.7%	2.86 [0.79; 9.83]	5.88 [2.31; 14.17]
Blister	NR	NR	NR	NR	1.06 [0.19; 5.78]	0.00 [0.00; 10.43]	NR
Dermatitis	9.53 [0.88; 24.54], 96.4%	2.23 [1.14; 3.62], 0.0%	0.72 [0.28; 1.84]	2.61 [1.02; 6.53]	1.62 [0.52; 3.18], 0.0%	0.00 [0.00; 5.20]	1.47 [0.26; 7.87]
Eczema	0.02 [0.00; 0.50], 0.0%	2.10 [0.99; 3.57], 0.0%	3.13 [0.24; 8.34], 80.2%	1.31 [0.36; 4.64]	2.88 [1.52; 5.37]	NR	NR
Erythema	1.95 [0.63; 3.78], 73.3%	3.52 [1.85; 5.65], 34.5%	2.32 [1.04; 4.05], 60.7%	3.27 [0.0140; 0.0742]	3.77 [1.08; 7.78], 66.4%	NR	NR
Neutrophilic dermatosis	NR	NR	0.58 [0.10; 3.20]	NR	NR	NR	NR
Night sweats	2.03 [0.14; 5.28], 0.0%	0.00 [0.00; 8.57]	1.95 [0.05; 5.86], 80.1%	0.65 [0.12; 3.61]	5.28 [2.78; 8.42], 0.0%	5.71 [2.24; 13.79]	5.88 [2.31; 14.17]
Pemphigoid	0.00 [0.00; 0.34], 0.0%	0.00 [0.00; 1.21]	0.18 [0.03; 1.01]	0.65 [0.12; 3.61]	0.32 [0.06; 1.79]	NR	NR
Photosensitivity reaction	NR	2.22 [0.46; 5.00], 37.5%	NR	0.65 [0.12; 3.61]	NR	NR	NR
Pruritus	26.78 [21.65; 32.23], 86.8%	21.43 [13.93; 29.99], 87.9%	22.36 [18.77; 26.16], 56.3%	41.18 [33.69; 49.10]	39.06 [31.68; 46.69], 67.8%	30.62 [9.91; 56.36], 84%	35.29 [25.00; 47.16]
Rash	31.07 [24.37; 38.17], 90.9%	19.62 [12.65; 27.65], 86.8%	17.14 [11.69; 23.35], 85.4%	41.83 [34.31; 49.75]	39.76 [20.91; 60.31], 95.7%	20.38 [2.81; 47.13], 86.6%	39.71 [28.93; 51.58]
Rash erythematous	0.92 [0.00; 3.01], 55.8%	1.12 [0.38; 3.24]	0.72 [0.28; 1.84]	1.96 [0.67; 5.61]	1.06 [0.19; 5.78]	NR	NR
Rash generalized	2.71 [0.03; 7.97], 75.0%	0.88 [0.04; 2.42], 21.5%	1.73 [0.59; 4.97]	1.31 [0.36; 4.64]	2.37 [1.02; 4.16], 0.0%	NR	NR
Rash macular	0.32 [0.06; 1.80]	0.88 [0.04; 2.42], 21.5%	NR	4.58 [2.23; 9.14]	2.47 [1.20; 4.10]. 0.0%	NR	NR
Rash maculo-papular	5.47 [1.60; 11.24], 92.8%	13.14 [2.61; 29.39], 97.2%	3.26 [1.13; 6.30], 80.1%	12.42 [8.10; 18.58]	13.84 [3.73; 28.67], 94.3%	17.14 [10.09; 27.62]	23.53 [15.03; 34.86]
Rash papular	1.15 [0.47; 2.07], 0.0%	1.68 [0.70; 3.03], 0.0%	0.36 [0.01; 1.01], 0.0%	0.65 [0.12; 3.61]	3.19 [0.79; 6.91], 55.2%	NR	NR
Rash pruritic	2.33 [1.21; 3.75], 53.4%	1.09 [0.00; 4.78], 75.3%	1.26 [0.61; 2.58]	5.88 [3.13; 10.80]	2.02 [0.87; 3.54], 0.0%	10.00 [4.93; 19.23]	1.47 [0.26; 7.87]
Skin exfoliation	5.00 [0.89; 23.61]	1.87 [0.51; 6.56]	NR	1.96 [0.67; 5.61]	NR	NR	NR
Skin hypopigmentation	0.23 [0.00; 1.55], 51.6%	2.24 [1.09; 4.54]	1.35 [0.49; 2.53], 7.3%	NR	3.51 [0.34; 9.23], 76.2%	NR	NR
Skin reactions	NR	NR	NR	8.50 [5.03; 13.99]	NR	NR	NR
Toxic skin eruption	0.00 [0.00; 0.31], 35.3%	0.32 [0.06; 1.79]	2.38 [0.42; 12.32]	3.92 [1.81; 8.29]	NR	NR	NR
Urticaria	0.96 [0.02; 2.76], 33.2%	1.87 [0.51; 6.56]	9.52 [3.77; 22.07]	0.65 [0.12; 3.61]	2.13 [0.59; 7.43]	0.00 [0.00; 10.43]	NR
Vitiligo	2.67 [1.05; 4.82], 67.9%	9.05 [5.41; 13.42], 72.9%	9.40 [7.99; 10.90], 0.0%	19.61 [14.09; 26.61]	9.96 [6.80; 13.60], 39.8%	6.41 [0.00; 34.52], 91.7%	14.71 [8.19; 25.00]
**Vascular disorders**							
Thromboembolic event	0.36 [0.00; 1.29], 54.4%	0.49 [0.07; 1.17], 0.0%	0.64 [0.19; 1.31], 0.0%	NR	0.79 [0.00; 3.46], 61.0%	4.29 [1.47; 11.86]	1.47 [0.26; 7.87]

*NR, not reported*.

#### Blood and Lymphatic System Disorders

All-grade lymphopenia was more likely to occur after pembrolizumab, with an incidence of 4.8%; however, the incidence of grade ≥3 lymphopenia was 0.8% in nivolumab users. In the FDA-approved doses, the incidence of all-grade and grade ≥3 lymphopenia reached 6.8 and 2.2% respectively, in nivolumab users.

#### Cardiac Disorders

The combination of ipilimumab and nivolumab resulted in an incidence of 1.1% of all-grade ventricular arrhythmia. Moreover, the incidence of ventricular arrhythmia and pericarditis (all-grade and grade ≥3) was 0.4% in nivolumab users.

#### Endocrine Disorders

The incidence of all-grade and grade ≥3 potential endocrine irAEs frequently occurred after the combination of pembrolizumab and ipilimumab or nivolumab and ipilimumab. Hyperthyroidism, hypothyroidism, hypophysitis, and thyroiditis were the most frequent potential endocrine irAEs. After anti-PD-1 therapy (nivolumab or pembrolizumab), the incidence of all-grade hyperthyroidism and hypothyroidism was 3.0–3.4 and 7.0–8.3%, respectively. After ipilimumab treatment, the observed incidence of all-grade and grade ≥3 hypophysitis and all-grade and grade ≥3 hypopituitarism was 4.1, 2.1, 1.5, and 1.0%, respectively. The estimated incidence of all-grade adrenal insufficiency and thyroiditis was 1.6% after nivolumab. On administering the FDA-approved dose of ipilimumab, the incidence of all-grade hypophysitis was 2.6% and that of grade ≥3 hypophysitis was 1.2%, with only <1% of patients developed hypopituitarism.

#### Eye Disorders

The incidence of all-grade uveitis was 2.6% after the combination of ipilimumab and pembrolizumab and 2.0% after the combination therapy with ipilimumab and nivolumab, but the incidence after monotherapies was <1%.

#### Gastrointestinal Disorders

The ICI combinations (ipilimumab and nivolumab or ipilimumab and pembrolizumab) resulted in the most frequent incidence of colitis (all-grade and grade ≥3), pancreatitis (all-grade and grade ≥3), diarrhea (all-grade), and autoimmune colitis (grade ≥3). Ipilimumab use was associated with developing diarrhea (all-grade in 29.2% and grade ≥3 in 5.9%) and colitis (all-grade in 8.0% and grade ≥3 in 5.4%).

#### General Disorders

All-grade infusion-related reaction was observed in 7.4% of users of the sequential therapy of nivolumab followed by ipilimumab and in 3.9% of users of the concomitant therapy of ipilimumab and pembrolizumab. However, among the monotherapies, nivolumab resulted in an incidence of 3.0% of all-grade infusion-related reactions.

#### Hepatobiliary Disorders

Hepatitis was the most common frequent potential irAE reported after ICI monotherapy or combination therapies. The incidence of all-grade and grade ≥3 hepatitis after the combination of pembrolizumab and ipilimumab was 9.8 and 5.9%, respectively; the corresponding values after the combination of nivolumab and ipilimumab were 4.9 and 3.5%, and those after the sequential therapy of nivolumab followed by ipilimumab were 4.4 and 4.4%. Nivolumab monotherapy resulted in an incidence of 3.0% of all-grade hepatitis.

#### Immune System Disorders

The incidence of all-grade hypersensitivity after nivolumab alone or in combination with ipilimumab including the FDA-approved doses was 2.0 and 2.9%, respectively.

#### Infections

The incidence of all-grade and grade ≥3 aseptic meningitis and encephalitis was 1.5% after sequential therapy of nivolumab followed by ipilimumab. Ipilimumab users had an incidence of 1.7% for all-grade conjunctivitis.

#### Investigations

The incidences of elevated or abnormal levels of most enzymes and hormones (all-grade) were reported at higher frequency after the combination of ICIs. The elevated enzymes and hormones that were reported frequently following the combination therapies included alanine aminotransferase (ALT), aspartate aminotransferase (AST), amylase, lipase, blood alkaline phosphatase, and blood bilirubin. The incidence of all-grade abnormal thyroid stimulating hormone (TSH) was 3.3 and 6.7% after concomitant combinations of ipilimumab and pembrolizumab or ipilimumab and nivolumab, respectively; the incidence of all-grade and grade ≥3 elevated gamma-glutamyl transferase (GGT) was, respectively, 7.2 and 1.3% after concomitant ipilimumab and pembrolizumab and 3.3 and 1.0% after concomitant ipilimumab and nivolumab. Among the monotherapies, after ipilimumab, the incidence of all-grade elevated ALT was 3.9%; after nivolumab, the incidence of all-grade and grade ≥3 elevated amylase was 5.8 and 1.5%, respectively, with an incidence of all-grade elevated blood alkaline phosphatase of 3.8% and an incidence of abnormal TSH of 2.8%. After pembrolizumab, the incidence of all-grade AST was 4.9%, that of all-grade elevated blood bilirubin was 1.6%, and that of all-grade elevated transaminases was 1.2%. For the FDA-approved doses, the combination of nivolumab and ipilimumab had the most frequent incidence of most all-grade and grade ≥3 elevated or abnormal enzymes or hormones. The most frequent enzymes and hormones abnormalities associated with nivolumab use were all-grade and grade ≥3 elevated lipase (3.1 and 2.2%, respectively), all-grade and grade ≥3 elevated amylase (5.8 and 1.5%, respectively), all-grade elevated blood alkaline phosphatase (3.8%), and all-grade elevated blood creatinine (1.1%). The incidence of all-grade elevated blood bilirubin, AST, ALT, and abnormal TSH was high among pembrolizumab users.

#### Metabolism and Nutritional Disorders

Type 1 diabetes mellitus (all-grade and grade ≥3) showed an incidence of 2.0% after the combination of ipilimumab and pembrolizumab and an incidence of 1.0% after the combination of ipilimumab and nivolumab. The incidence of all-grade hyperglycemia was 7.4% after sequential nivolumab followed by ipilimumab and that of grade ≥3 hyperglycemia was 2.9% after sequential ipilimumab followed by nivolumab. After the FDA-approved dose of pembrolizumab, the incidence of both all-grade and grade ≥3 type 1 diabetes mellitus was 2.4% and that of all-grade hyperglycemia was 5.6%. The incidence of all-grade and grade ≥3 hyperglycemia was 2.6 and 1.6%, respectively, after the FDA-approved doses of the combination of nivolumab and ipilimumab.

#### Musculoskeletal and Connective Tissue Disorders

The most common musculoskeletal and connective disorders, i.e., potential irAEs, were arthralgia and myalgia. They were reported frequently after combination therapies. Among monotherapies, pembrolizumab users had the most frequent incidence of all-grade arthralgia (12.2%), all-grade myalgia (5.9%), and all-grade arthritis (1.2%). Moreover, the incidence of all-grade muscle spasm was 3.4% after nivolumab. The most frequent potential irAEs among ipilimumab monotherapy users were all-grade arthralgia (6.3%) and all-grade myalgia (3.2%). Regarding the FDA-approved doses of ICIs, the combination of nivolumab and ipilimumab resulted in the highest incidence of all-grade arthralgia (14.6%), all-grade myalgia (11.9%), all-grade muscle spasm (2.2%), all-grade polymyositis (2.1%), and all-grade rhabdomyolysis (1.1%). After the FDA-approved doses of monotherapies, the incidence of all-grade arthralgia and myalgia was, respectively, 6.2 and 3.2% for ipilimumab; 9.4 and 4.2% for nivolumab; and 7.7 and 4.9% for pembrolizumab. All-grade muscle spasm was reported in 3.4% of nivolumab users.

#### Nervous System Disorders

The combination of nivolumab and ipilimumab resulted in an incidence of 3.5% for all-grade peripheral neuropathy. The incidence of all-grade myasthenia gravis and neuropathy was 1.5% after sequential nivolumab followed by ipilimumab. After pembrolizumab and ipilimumab combination therapy, the incidence of all-grade lethargy was 5.9% and that of all-grade peripheral neuropathy was 2.6%. Among pembrolizumab users, the incidence of all-grade lethargy was 1.1%. The incidence of all-grade peripheral neuropathy was 2.4% after nivolumab monotherapy. The incidence of irAEs was similar after FDA-approved doses of the combination of nivolumab and ipilimumab as well as nivolumab and pembrolizumab therapies, as described above. Moreover, the incidence of myasthenia gravis was 1.3% after ipilimumab.

#### Renal and Urinary Disorders

The incidence of all-grade and grade ≥3 acute renal failure was 1.3 and 1.1%, respectively, after combination nivolumab and ipilimumab. Sequential nivolumab followed by ipilimumab resulted in an incidence of 1.5% for all-grade and grade ≥3 acute renal failure. All-grade nephritis was observed in 1.3% of patients receiving the combination of pembrolizumab and ipilimumab and in 1% of those receiving nivolumab and ipilimumab combination therapy. After ICI monotherapies, the frequency of renal and urinary disorders with potential irAEs was <1%. The incidence of potential irAEs related to the renal system was also <1% after FDA-approved doses of ICI combination or monotherapies.

#### Respiratory, Thoracic, and Mediastinal Disorders

Pneumonitis (all-grade and grade ≥3) was the most common potential respiratory irAE reported in the included trials, especially after combination therapies. The incidence of all-grade pneumonitis was 2.0% after pembrolizumab and 1.4% after nivolumab monotherapies. After ipilimumab, the incidence of all-grade alveolitis was 15%, while after nivolumab, the incidence of all-grade pulmonary toxicity was 4.4%. After the FDA-approved dose of ICIs, the incidence of all-grade pneumonitis was 7.5% after combination nivolumab and ipilimumab, 1.5% after pembrolizumab, and 1.4% after nivolumab.

#### Skin and Subcutaneous Tissue Disorders

Combination therapies resulted in frequent potential skin irAEs, especially all-grade alopecia, pruritus, rash, macular rash, maculopapular rash, toxic skin eruptions, vitiligo, and grade ≥3 pruritus and rash. After nivolumab and ipilimumab combination therapy, the incidence of grade ≥3 rash was 3.6% and that of maculopapular rash was 1.4%. After ipilimumab, all-grade rash was observed in 31.1% of patients, pruritus in 26.8%, dermatitis in 9.5%, skin exfoliation in 5.0%, generalized rash in 2.7%, pruritic rash in 2.3%, and night sweats in 2.0%. After pembrolizumab, the incidence of all-grade eczema was 3.1%, that of toxic skin eruptions was 2.4%, that of urticaria was 9.5%, and that of vitiligo was 9.4%. After nivolumab, all-grade alopecia was observed in 2.0% of patients, erythema in 3.5%, photosensitivity reaction in 2.2%, erythematous rash in 1.1%, maculopapular rash in 13.1%, papular rash in 1.7%, and skin hypopigmentation in 2.2%. After the FDA approved doses of the combination of nivolumab and ipilimumab, the incidence of all-grade pruritus and rash and grade ≥3 rash was 33.7, 30.7, and 3.0%, respectively. The most frequent potential skin-related irAEs after the FDA-approved dose of ipilimumab included all-grade rash (20.5%), pruritus (24.9%), dermatitis (4.7%), generalized rash (1.3%), pruritic rash (2.4%), and night sweats (2.0%). The FDA-approved dose of pembrolizumab was associated with all-grade alopecia (3.4%), eczema (4.5%), erythema (4.7%), toxic skin eruptions (2.4%), and urticaria (9.5%). The FDA-approved dose of nivolumab resulted in all-grade photosensitivity reaction (3.8%), maculopapular rash (16.1%), papular rash (1.7%), skin hypopigmentation (2.2%), and vitiligo (9.1%).

#### Vascular Disorders

The incidence of all-grade and grade ≥3 thromboembolic events was most frequent after the combination therapies. After monotherapies, the incidence of all-grade and grade ≥3 thromboembolic events was <1%; however, the FDA-approved dose of pembrolizumab resulted in an incidence of 1.1% for all-grade thromboembolic events.

### Heterogeneity

A high heterogeneity (*I*^2^ > 75%) was observed in 60 outcomes (Tables 2 and 3). Restricting the doses to the FDA-approved doses of ICIs decreased the heterogeneity in 21 outcomes to *I*^2^ <75% (Tables 4 and 5). Subgroup analyses were not feasible because the data were presented from two trials only in 10 outcomes of sequential ipilimumab followed by nivolumab (Figures S17–S26), 4 outcomes of nivolumab monotherapy (Figures S27–S30), and 1 outcome of ipilimumab and nivolumab combination therapy (Figure S31). For the remaining outcomes, subgroup analysis based on the prior systemic anticancer therapy use showed a reduction in the heterogeneity in most studies that included previously untreated patients (Figures S32–S55). There were 7 more outcomes in the FDA-approved doses of ICIs with high heterogeneity (Tables 4 and 5). Four outcomes were based on 2 studies, and subgrouping was not applicable (Figures S56–S59). In the remaining 3 outcomes, subgrouping based on prior systemic anticancer therapy showed a low heterogeneity in previously untreated patients (Figures S60–S62).

**Table 3:** Incidence of Grades ≥ 3 Potential Immune-Related Adverse Events (irAEs) in Advanced Melanoma presented as % with 95% confidence interval, and *I*^2^ for the % of heterogeneity (*I*^2^ not reported if the outcome was obtained from one study).

**irAEs**	**Ipilimumab**	**Nivolumab**	**Pembrolizumab**	**Pembrolizumab +Ipilimumab**	**Nivolumab +Ipilimumab**	**Ipilimumab then Nivolumab (Sequential)**	**Nivolumab then Ipilimumab (Sequential)**
Number of studies	21	7	5	1	5	2	1
Overall incidence	19.50 [14.52; 24.99], 90.0%	4.00 [1.00; 6.00], 66.0%	4.78 [2.65; 7.41], 69.0%	27.45 [21.00; 35.01]	36.00 [21.00; 52.00], 93.0%	13.00 [3.00; 29.00], 71.2%	15.00 [8.00; 25.00]
**Blood and lymphatic system disorders**							
Lymphopenia	0.00 [0.00; 3.89], 0.0%	0.76 [0.00; 5.65], 83.4%	0.00 [0.00; 8.38]	NR	NR	NR	NR
**Cardiac disorders**							
Pericarditis	0.14 [0.02; 0.78]	0.37 [0.07; 2.08]	0.15 [0.03; 0.86]	NR	NR	NR	NR
Ventricular arrhythmia	0.00 [0.00; 7.71]	0.37 [0.07; 2.08]	NR	NR	0.00 [0.00; 3.93]	NR	NR
**Endocrine disorders**							
Acute adrenocortical insufficiency	0.14 [0.00; 0.53], 0.0%	0.00 [0.00; 1.21]	0.28 [0.05; 1.57]	0.65 [0.12; 3.61]	0.32 [0.06; 1.79]	NR	NR
Adrenal insufficiency	0.06 [0.00; 0.41], 11.7%	0.77 [0.07; 1.97], 25.2%	0.33 [0.03; 0.82], 0.0%	0.65 [0.12; 3.61]	1.57 [0.63; 2.84], 0.0%	0.58 [0.00; 5.93], 50.5%	4.41 [1.51; 12.19]
Adrenocorticotropic hormone deficiency	0.14 [0.02; 0.78]	NR	NR	0.00 [0.00; 2.45]	NR	NR	NR
Autoimmune thyroiditis	0.00 [0.00; 0.16], 0.0%	0.00 [0.00; 1.21]	NR	NR	0.36 [0.00; 1.36], 0.0%	NR	NR
Hyperthyroidism	0.10 [0.00; 0.43], 1.0%	0.00 [0.00; 0.09], 0.0%	0.00 [0.00; 0.13], 0.0%	1.31 [0.36; 4.64]	0.66 [0.00; 2.08], 25.1%	0.00 [0.00; 10.43]	NR
Hypophysitis	2.06 [0.87; 3.60], 69.0%	0.15 [0.00; 0.87], 0.0%	0.15 [0.00; 0.58], 19.8%	1.96 [0.67; 5.61]	2.36 [1.01; 4.14], 20.6%	2.78 [0.19; 7.31], 0.0%	0.00 [0.00; 5.35]
Hypopituitarism	0.96 [0.27; 1.95], 38.8%	0.08 [0.00; 0.71], 0.0%	0.14 [0.00; 0.59], 48.4%	0.00 [0.00; 2.45]	0.43 [0.00; 1.71], 0.0%	NR	NR
Hypothyroidism	0.00 [0.00; 0.05], 0.0%	0.00 [0.00; 0.07], 0.0%	0.00 [0.00; 0.18], 0.0%	0.00 [0.00; 2.45]	0.08 [0.00; 0.69], 0.0%	0.00 [0.00; 1.73], 0.0%	0.00 [0.00; 5.35]
Lymphocytic hypophysitis	0.01 [0.00; 0.27], 0.0%	0.00 [0.00; 1.21]	NR	0.00 [0.00; 2.45]	0.32 [0.06; 1.79]	NR	NR
Thyroiditis	0.02 [0.00; 0.25], 0.0%	0.00 [0.00; 0.15], 0%	0.00 [0.00; 0.16], 0.0%	0.00 [0.00; 2.45]	0.02 [0.00; 0.67], 0.0%	0.00 [0.00; 10.43]	NR
Thyrotoxic crisis	0.14 [0.02; 0.78]	NR	NR	NR	NR	NR	NR
**Eye disorders**							
Uveitis	0.09 [0.00; 0.79], 47.1%	0.00 [0.00; 0.22], 0.0%	0.00 [0.00; 0.05], 0.0%	0.00 [0.00; 2.45]	0.42 [0.00; 2.79], 61.9%	0.00 [0.00; 10.43]	NR
**Gastrointestinal disorders**							
Autoimmune colitis	0.71 [0.17; 1.49], 11.7%	0.32 [0.06; 1.79]	0.28 [0.05; 1.57]	1.96 [0.67; 5.61]	1.04 [0.00; 3.33], 57.7%	1.43 [0.25; 7.66]	0.00 [0.00; 5.35]
Autoimmune pancreatitis	0.00 [0.00; 0.01], 0.0%	0.32 [0.06; 1.79]	0.18 [0.03; 1.01]	0.65 [0.12; 3.61]	0.17 [0.00; 2.18], 62.6%	0.00 [0.00; 5.20]	0.00 [0.00; 5.35]
Colitis	5.41 [4.09; 6.89], 44.9%	0.39 [0.02; 1.04], 0.0%	1.10 [0.57; 1.75], 3.7%	7.19 [4.06; 12.41]	9.42 [6.51; 12.77], 33%	6.87 [0.00; 37.07], 92.5%	14.71 [8.19; 25.00]
Diarrhea	5.91 [4.35; 7.67], 57.6%	0.92 [0.19; 2.03], 34.9%	0.70 [0.26; 1.28], 0.0%	0.65 [0.12; 3.61]	7.20 [4.31; 10.70], 47.3%	2.76 [0.00; 13.30], 71.2%	11.76 [6.08; 21.53]
Enterocolitis	1.25 [0.00; 4.02], 84.2%	NR	0.00 [0.00; 0.21], 0.0%	NR	0.44 [0.00; 1.39], 0.0%	1.43 [0.25; 7.66]	0.00 [0.00; 5.35]
Frequent bowel movements	0.00 [0.00; 1.48]	0.00 [0.00; 1.83]	0.00 [0.00; 0.69]	0.00 [0.00; 2.45]	NR	NR	NR
Gastrointestinal perforation	0.28 [0.03; 0.69], 0.0%	0.10 [0.00; 0.72], 16.3%	0.15 [0.00; 1.11], 70.1%	NR	0.63 [0.02; 1.79], 0.0%	NR	NR
Gastrointestinal toxicity	NR	4.35 [0.77; 20.99]	NR	NR	NR	NR	NR
Pancreatitis	0.02 [0.00; 0.37], 21.0%	0.46 [0.02; 1.28], 0.0%	0.18 [0.01; 0.50], 0.0%	0.65 [0.12; 3.61]	0.75 [0.03; 2.09], 21.8%	1.45 [0.00; 5.25]	0.00 [0.00; 5.35]
Peritonitis	0.04 [0.00; 0.41], 0.0%	0.32 [0.06; 1.79]	NR	NR	0.32 [0.06; 1.79]	NR	NR
Rectal hemorrhage	0.16 [0.00; 0.55], 0.0%	NR	NR	NR	0.14 [0.00; 0.95], 0.0%	NR	NR
Ulcerative colitis	0.28 [0.08; 1.00]	NR	NR	NR	NR	NR	NR
**General disorders**							
Infusion-related reaction	0.00 [0.00; 0.24], 0.0%	0.00 [0.00; 0.32], 0.0%	0.00 [0.00; 1.06]	0.65 [0.12; 3.61]	0.00 [0.00; 0.28], 0.0%	0.00 [0.00; 1.58], 0.0%	0.00 [0.00; 5.35]
**Hepatobiliary disorders**							
Acute hepatic failure	0.19 [0.00; 1.13], 38.6%	NR	NR	NR	NR	NR	NR
Acute hepatitis	0.06 [0.00; 0.39], 0.0%	NR	NR	NR	0.32 [0.06; 1.79]	NR	NR
Autoimmune hepatitis	0.45 [0.01; 1.29], 41.4%	0.64 [0.18; 2.30]	0.51 [0.12; 1.07], 0.0%	NR	1.62 [0.52; 3.18], 0.0%	NR	NR
Hepatitis	0.16 [0.00; 0.63], 17.1%	0.95 [0.00; 10.35], 85.5%	0.43 [0.14; 0.85], 0.0%	5.88 [3.13; 10.80]	3.45 [0.32; 8.92], 80.5%	0.00 [0.00; 5.20]	4.41 [1.51; 12.19]
Hepatocellular injury	0.19 [0.00; 0.63], 6.3%	0.51 [0.04; 1.33], 0.0%	0.00 [0.00; 0.69]	NR	0.63 [0.02; 1.79], 0.0%	NR	NR
Hepatotoxicity	0.33 [0.01; 0.92], 34.0%	0.00 [0.00; 0.61], 0.0%	0.00 [0.00; 0.69]	NR	2.56 [1.30; 4.96]	0.00 [0.00; 5.20]	1.47 [0.26; 7.87]
Hyperbilirubinemia	0.00 [0.00; 0.30], 13.7%	0.00 [0.00; 1.21]	0.00 [0.00; 1.06]	NR	0.00 [0.00; 1.21]	1.43 [0.25; 7.66]	0.00 [0.00; 5.35]
**Immune system disorders**							
Episcleritis	0.00 [0.00; 1.69], 0.0%	NR	NR	NR	NR	NR	NR
Hypersensitivity	0.00 [0.00; 0.10], 0.0%	0.11 [0.00; 0.59], 0.0%	0.00 [0.00; 0.21], 0.0%	0.00 [0.00; 2.45]	0.00 [0.00; 1.21]	NR	NR
**Infections**							
Aseptic meningitis	0.00 [0.00; 0.94], 31.6%	0.00 [0.00; 1.21]	NR	0.65 [0.12; 3.61]	0.32 [0.06; 1.79]	0.00 [0.00; 5.20]	1.47 [0.26; 7.87]
Conjunctivitis	0.06 [0.00; 1.14], 34.9%	NR	0.00 [0.00; 2.77]	0.00 [0.00; 2.45]	NR	NR	NR
Encephalitis	0.10 [0.00; 0.42], 0.0%	NR	0.00 [0.00; 0.69]	NR	0.32 [0.06; 1.79]	0.00 [0.00; 5.20]	1.47 [0.26; 7.87]
**Investigations**							
Alanine aminotransferase (increased)	0.82 [0.24; 1.62], 46.1%	0.53 [0.09; 1.21], 0.0%	0.02 [0.00; 0.30], 0.0%	1.96 [0.67; 5.61]	10.21 [7.58; 13.16], 15.6%	1.45 [0.00; 5.25], 0.0%	10.29 [05.08; 19.76]
Amylase (increased)	0.02 [0.00; 0.38], 5.9%	1.51 [0.34; 3.26], 0.0%	NR	3.92 [1.81; 8.29]	3.54 [2.00; 5.42], 10.7%	4.69 [1.12; 10.00], 0.0%	4.41 [1.51; 12.19]
Aspartate aminotransferase (increased)	0.76 [0.10; 1.79], 60.1%	0.27 [0.00; 0.83], 0.0%	0.09 [0.00; 0.46], 0.0%	0.00 [0.00; 2.45]	8.52 [5.41; 12.21], 46.6%	0.67 [0.00; 3.87], 0.0%	8.82 [4.11; 17.94]
Blood alkaline phosphatase (increased)	0.07 [0.00; 0.51], 28.3%	0.00 [0.00; 0.32]	0.00 [0.00; 0.15], 0.0%	0.00 [0.00; 2.45]	0.41 [0.00; 1.44], 17.3%	0.00 [0.00; 5.20]	2.94 [0.81; 10.10]
Blood bilirubin (increased)	0.01 [0.00; 0.56], 55.7%	0.00 [0.00; 0.16], 0.0%	0.00 [0.00; 0.21], 0.0%	0.00 [0.00; 2.45]	0.01 [0.00; 0.45], 0.0%	0.00 [0.00; 5.20]	0.00 [0.00; 5.35]
Blood corticotropin (decreased)	0.00 [0.00; 0.45], 6.2%	NR	0.18 [0.03; 1.01]	NR	NR	NR	NR
Blood creatinine (increased)	0.00 [0.00; 0.00], 0.0%	0.00 [0.00; 0.25], 0.0%	0.00 [0.00; 0.04], 0.0%	0.00 [0.00; 2.45]	0.68 [0.00; 2.32], 37.1%	0.00 [0.00; 1.73], 0.0%	1.47 [0.26; 7.87]
Blood thyroid stimulating hormone (abnormal)	0.00 [0.00; 0.52], 0.0%	0.16 [0.00; 1.88], 55.9%	0.00 [0.00; 0.21], 0.0%	0.00 [0.00; 2.45]	0.00 [0.00; 1.25], 0.0%	0.00 [0.00; 1.73], 0.0%	0.00 [0.00; 5.35]
Blood urea (increased)	NR	NR	NR	NR	0.0000 [0.0000; 0.0393]	NR	NR
Gamma-glutamyl transferase (increased)	0.14 [0.00; 0.54], 0.0%	0.00 [0.00; 0.22], 0.0%	NR	1.31 [0.36; 4.64]	1.01 [0.14; 2.37], 0.0%	0.00 [0.00; 10.43]	NR
Hepatic enzyme (increased)	0.06 [0.00; 0.39], 0.0%	0.10 [0.00; 0.72], 16.3%	0.28 [0.05; 1.57]	0.65 [0.12; 3.61]	0.96 [0.33; 2.78]	0.00 [0.00; 5.20]	1.47 [0.26; 7.87]
Lipase (increased)	0.98 [0.00; 3.12], 78.5%	2.22 [0.01; 6.83], 65.6%	NR	16.34 [11.32; 23.01]	10.36 [8.03; 12.94], 0.0%	11.96 [3.43; 24.13], 56.8%	14.71 [8.19; 25.00]
Liver function test (abnormal)	0.00 [0.00; 0.23], 0.0%	0.64 [0.07; 1.63], 17.8%	0.18 [0.03; 1.01]	0.00 [0.00; 2.45]	0.36 [0.00; 1.36], 0.0%	NR	NR
Thyroxine free (decreased)	NR	0.00 [0.00; 3.47]	0.00 [0.00; 2.17]	0.00 [0.00; 2.45]	NR	NR	NR
Thyroxine (increased)	NR	NR	0.00 [0.00; 2.17]	NR	NR	NR	NR
Transaminases (increased)	0.13 [0.00; 0.67], 19.1%	0.32 [0.06; 1.79]	0.57 [0.00; 9.07], 70.7%	0.00 [0.00; 2.45]	1.53 [0.29; 3.45], 44.6%	NR	NR
**Metabolism and nutrition disorders**							
Diabetes mellitus	0.07 [0.00; 4.52], 57.7%	0.08 [0.00; 0.73], 0.0%	0.05 [0.00; 0.96], 67.2%	0.65 [0.12; 3.61]	0.64 [0.18; 2.30]	NR	NR
Diabetic ketoacidosis	0.00 [0.00; 0.36], 35.8%	0.11 [0.00; 0.94], 35.4%	0.18 [0.03; 1.01]	0.65 [0.12; 3.61]	0.36 [0.00; 1.36], 0.0%	NR	NR
Hyperglycemia	0.00 [0.00; 0.16], 14.6%	0.44 [0.04; 1.12], 0.0%	0.51 [0.00; 3.95], 55.3%	0.00 [0.00; 2.45]	1.61 [0.52; 3.18], 0.0%	2.86 [0.79; 9.83]	1.47 [0.26; 7.87]
Hyperlipasemia	0.14 [0.02; 0.78]	NR	NR	NR	NR	NR	NR
Type 1 diabetes mellitus	0.00 [0.00; 1.48]	NR	0.05 [0.00; 0.96], 67.2%	1.96 [0.67; 5.61]	1.06 [0.19; 5.78]	NR	NR
**Musculoskeletal and connective tissue disorders**							
Arthralgia	0.16 [0.00; 0.52], 7.4%	0.00 [0.00; 0.12], 0.0%	0.00 [0.00; 0.06], 7.8%	0.65 [0.12; 3.61]	0.08 [0.00; 0.68], 0.0%	0.00 [0.00; 1.73], 0.0%	1.47 [0.26; 7.87]
Arthritis	0.03 [0.00; 0.51], 24.8%	0.10 [0.00; 0.72], 16.3%	0.00 [0.00; 0.06], 0.0%	0.00 [0.00; 2.45]	0.32 [0.06; 1.79]	0.00 [0.00; 5.20]	0.00 [0.00; 5.35]
Arthropathy	0.00 [0.00; 1.22]	0.00 [0.00; 1.21]	0.00 [0.00; 2.17]	NR	0.32 [0.06; 1.79]	NR	NR
Hand-foot-syndrome (Palmar-plantar erythrodysesthesia syndrome)	0.00 [0.00; 3.60]	NR	NR	NR	NR	NR	NR
Joint swelling	NR	NR	0.00 [0.00; 2.17]	0.00 [0.00; 2.45]	NR	NR	NR
Muscle spasms	0.00 [0.00; 0.25], 0.0%	0.00 [0.00; 0.33], 0.0%	0.00 [0.00; 0.17]	0.00 [0.00; 2.45]	0.64 [0.18; 2.30]	0.00 [0.00; 5.20]	1.47 [0.26; 7.87]
Myalgia	0.00 [0.00; 0.05], 0.0%	0.00 [0.00; 0.23], 0.0%	0.14 [0.00; 0.59], 48.4%	0.00 [0.00; 2.45]	0.01 [0.00; 0.56], 0.0%	0.00 [0.00; 5.20]	0.00 [0.00; 5.35]
Myopathy	NR	NR	0.00 [0.00; 0.58]	0.00 [0.00; 2.45]	NR	NR	NR
Polymyalgia rheumatica	0.06 [0.00; 0.64], 19.1%	NR	NR	0.00 [0.00; 2.45]	NR	NR	NR
Polymyositis/ myositis	0.00 [0.00; 0.34], 0.0%	0.32 [0.06; 1.79]	0.00 [0.00; 0.16], 0.0%	0.00 [0.00; 2.45]	1.06 [0.19; 5.78]	NR	NR
Rhabdomyolysis	0.00 [0.00; 3.89]	NR	0.15 [0.03; 0.86]	NR	1.06 [0.19; 5.78]	NR	NR
**Nervous system disorders**							
Guillain-Barre syndrome	0.01 [0.00; 0.23], 0.0%	0.11 [0.00; 0.94], 35.4%	0.18 [0.03; 1.01]	NR	0.36 [0.00; 1.36], 0.0%	NR	NR
Lethargy	0.04 [0.00; 0.67], 35.9%	0.00 [0.00; 1.21]	0.00 [0.00; 0.21], 0.0%	0.00 [0.00; 2.45]	0.32 [0.06; 1.79]	NR	NR
Myasthenia gravis	1.33 [0.24; 7.17]	NR	NR	NR	NR	0.00 [0.00; 5.20]	1.47 [0.26; 7.87]
Myelitis	0.39 [0.07; 2.18]	NR	0.00 [0.00; 0.69]	NR	NR	NR	NR
Neuropathy	0.00 [0.00; 0.63], 0.0%	NR	NR	0.00 [0.00; 2.45]	0.32 [0.06; 1.79]	0.00 [0.00; 5.20]	1.47 [0.26; 7.87]
Peripheral neuropathy	0.00 [0.00; 0.10], 7.8%	0.00 [0.00; 0.41], 0.0%	0.00 [0.00; 0.07], 0.0%	0.00 [0.00; 2.45]	0.02 [0.00; 0.67], 0.0%	NR	NR
**Renal and urinary disorders**							
Acute renal failure	0.05 [0.00; 0.46], 29.5%	0.04 [0.00; 0.44], 0.0%	0.78 [0.30; 1.44], 0.0%	NR	1.06 [0.24; 2.27], 0.0%	0.00 [0.00; 1.73], 0.0%	1.47 [0.26; 7.87]
Nephritis	0.14 [0.00; 0.50], 0.0	0.12 [0.00; 0.76], 0.0%	0.00 [0.00; 0.26], 19.5%	0.65 [0.12; 3.61]	0.13 [0.00; 1.31], 22.6%	0.00 [0.00; 10.43]	NR
Nephrotoxicity	NR	0.00 [0.00; 14.31]	NR	NR	0.32 [0.06; 1.79]	NR	NR
Renal failure	0.23 [0.00; 1.03], 53.2%	0.17 [0.00; 0.89], 42.1%	0.56 [0.17; 1.12], 0.0%	NR	0.32 [0.06; 1.79]	0.67 [0.00; 3.87], 0.0%	1.47 [0.26; 7.87]
**Respiratory, thoracic, and mediastinal disorders**							
Alveolitis	1.52 [0.27; 08.10]	NR	0.00 [0.00; 1.06]	NR	NR	NR	NR
Lung infiltration	0.25 [0.00; 4.18], 71.1%	0.00 [0.00; 0.38], 0.0%	NR	NR	0.32 [0.06; 1.79]	NR	NR
Pneumonitis	0.25 [0.02; 0.64], 0.0%	0.00 [0.00; 0.23], 0.0%	0.11 [0.00; 0.47], 0.0%	1.96 [0.67; 5.61]	1.29 [0.43; 2.48], 0.0%	0.00 [0.00; 1.73], 0.0%	2.94 [0.81; 10.10]
Pulmonary toxicity	NR	0.00 [0.00; 14.31]	NR	NR	NR	NR	NR
**Skin and subcutaneous tissue disorders**							
Alopecia	0.00 [0.00; 0.05], 0.0%	0.00 [0.00; 0.20], 0.0%	0.00 [0.00; 0.21], 0.0%	0.00 [0.00; 2.45]	0.00 [0.00; 1.29], 0.0%	0.00 [0.00; 5.20]	0.00 [0.00; 5.35]
Blister	NR	NR	NR	NR	0.00 [0.00; 3.93]	0.00 [0.00; 10.43]	NR
Dermatitis	0.00 [0.00; 0.42], 26.3%	0.00 [0.00; 0.33], 0.0%	0.00 [0.00; 0.69]	0.00 [0.00; 2.45]	0.00 [0.00; 0.38], 0.0%	0.00 [0.00; 5.20]	0.00 [0.00; 5.35]
Eczema	0.00 [0.00; 0.02], 0.0%	0.00 [0.00; 0.36], 0.0%	0.00 [0.00; 0.05], 0.0%	0.00 [0.00; 2.45]	0.00 [0.00; 1.21]	NR	NR
Erythema	0.00 [0.00; 0.02], 0.0%	0.00 [0.00; 0.21], 0.0%	0.00 [0.00; 0.13], 0.0%	0.00 [0.00; 2.45]	0.09 [0.00; 0.75], 0.0%	NR	NR
Neutrophilic dermatosis	NR	NR	0.00 [0.00; 2.17]	NR	NR	NR	NR
Night sweats	0.00 [0.00; 0.90], 0.0%	0.00 [0.00; 8.57]	0.00 [0.00; 2.17]	0.00 [0.00; 2.45]	0.00 [0.00; 1.00], 0.0%	0.00 [0.00; 10.43]	0.00 [0.00; 5.35]
Pemphigoid	0.00 [0.00; 0.34], 0.0%	0.00 [0.00; 1.21]	0.18 [0.03; 1.01]	0.65 [0.12; 3.61]	0.32 [0.06; 1.79]	NR	NR
Photosensitivity reaction	NR	0.00 [0.00; 0.58], 0.0%	NR	0.00 [0.00; 2.45]	NR	NR	NR
Pruritus	0.13 [0.00; 0.45], 5.9%	0.01 [0.00; 0.34], 0.0%	0.00 [0.00; 0.01], 0.0%	0.00 [0.00; 2.45]	0.63 [0.04; 1.65], 5.8%	0.00 [0.00; 1.73], 0.0%	1.47 [0.26; 7.87]
Rash	0.51 [0.18; 0.96], 0.0%	0.18 [0.00; 0.73], 3.8%	0.02 [0.00; 0.78], 67.1%	2.61 [1.02; 6.53]	3.57 [1.96; 5.57], 16.9%	1.45 [0.00; 5.25], 0.0%	2.94 [0.81; 10.10]
Rash erythematous	0.00 [0.00; 0.12], 0.0%	0.00 [0.00; 1.41]	0.00 [0.00; 0.69]	0.00 [0.00; 2.45]	0.00 [0.00; 3.93]	NR	NR
Rash generalized	0.00 [0.00; 0.26], 0.0%	0.00 [0.00; 0.38], 0.0%	0.00 [0.00; 2.17]	0.00 [0.00; 2.45]	0.79 [0.00; 3.46], 61.0%	NR	NR
Rash macular	0.32 [0.06; 1.80]	0.00 [0.00; 0.38], 0.0%	NR	0.65 [0.12; 3.61]	0.00 [0.00; 0.38], 0.0%	NR	NR
Rash maculo-papular	0.90 [0.00; 2.92], 83.7%	0.29 [0.00; 1.48], 59.4%	0.12 [0.00; 0.51], 0.0%	0.65 [0.12; 3.61]	1.38 [0.00; 4.68], 78.3%	0.00 [0.00; 5.20]	1.47 [0.26; 7.87]
Rash papular	0.00 [0.00; 0.34], 0.0%	0.00 [0.00; 0.36], 0.0%	0.00 [0.00; 0.21]	0.00 [0.00; 2.45]	0.17 [0.00; 2.18], 62.6%	NR	NR
Rash pruritic	0.00 [0.00; 0.06], 0.0%	0.00 [0.00; 0.38], 0.0%	0.00 [0.00; 0.69]	1.31 [0.36; 4.64]	0.00 [0.00; 0.31], 0.0%	10.00 [4.93; 19.23]	0.00 [0.00; 5.35]
Skin exfoliation	0.00 [0.00; 16.11]	0.00 [0.00; 3.47]	NR	0.00 [0.00; 2.45]	NR	NR	NR
Skin hypopigmentation	0.00 [0.00; 0.16], 0.0%	0.00 [0.00; 1.21]	0.00 [0.00; 0.05], 0.0%	NR	0.00 [0.00; 0.38], 0.0%	NR	NR
Skin reactions	NR	NR	NR	7.84 [4.54; 13.21]	NR	NR	NR
Toxic skin eruption	0.00 [0.00; 0.18], 31.5%	0.32 [0.06; 1.79]	0.00 [0.00; 8.38]	1.31 [0.36; 4.64]	NR	NR	NR
Urticaria	0.21 [0.00; 2.44], 0.0%	0.00 [0.00; 3.47]	0.00 [0.00; 8.38]	0.00 [0.00; 2.45]	0.00 [0.00; 3.93]	0.00 [0.00; 10.43]	NR
Vitiligo	0.00 [0.00; 0.05], 0.0%	0.00 [0.00; 0.11], 0.0%	0.00 [0.00; 0.01], 0.0%	0.00 [0.00; 2.45]	0.00 [0.00; 0.18], 0.0%	0.00 [0.00; 1.73], 0.0%	0.00 [0.00; 5.35]
**Vascular disorders**							
Thromboembolic event	0.16 [0.00; 0.71], 22.1%	0.49 [0.07; 1.17], 0.0%	0.64 [0.19; 1.31], 0.0%	NR	0.79 [0.00; 3.46], 61.0%	4.29 [1.47; 11.86]	1.47 [0.26; 7.87]

*NR, not reported*.

**Table 4:** Incidence of All-grades Potential Immune-Related Adverse Events (irAEs) in Advanced Melanoma presented as % with 95% confidence interval, and *I*^2^ for the % of heterogeneity for the FDA approved doses (*I*^2^ not reported if the outcome was obtained from one study).

**irAEs**	**Ipilimumab**	**Nivolumab**	**Pembrolizumab**	**Nivolumab +Ipilimumab**
Number of studies	10	7	4	5
Overall incidence	53.96 [42.21; 64.97], 94.0%	44.00 [31.00; 57.00], 93.0%	20.28 [14.23; 27.06], 41.0%	80.00 [61.00; 94.00], 95.0%
**Blood and lymphatic system disorders**				
Lymphopenia	2.38 [0.42; 5.49], 0.0%	6.76 [0.00; 28.48], 81.0%	4.76 [1.32; 15.79]	NR
**Cardiac disorders**				
Pericarditis	0.00 [0.00; 1.05]	0.37 [0.07; 2.08]	NR	NR
Ventricular arrhythmia	0.00 [0.00; 7.71]	0.37 [0.0007; 2.08]	NR	1.06 [0.19; 5.78]
**Endocrine disorders**				
Acute adrenocortical insufficiency	0.10 [0.00; 0.58], 0.0%	0.00 [0.00; 1.21]	0.00 [0.00; 2.11]	0.32 [0.06; 1.79]
Adrenal insufficiency	0.67 [0.20; 1.32], 0.0%	1.68 [0.16; 4.34], 68.1%	0.68 [0.00; 2.24], 0.0%	3.79 [2.14; 5.79], 0.0%
Adrenocorticotropic hormone deficiency	0.28 [0.05; 1.55]	NR	NR	NR
Autoimmune thyroiditis	0.00 [0.00; 0.18], 0.0%	0.32 [0.06; 1.79]	NR	0.96 [0.01; 2.90], 35.8%
Hyperthyroidism	0.88 [0.03; 2.54], 75.5%	3.17 [1.98; 4.59], 7.4%	3.83 [1.50; 6.97], 0.0%	10.59 [6.18; 15.87], 40.9%
Hypophysitis	2.58 [1.73; 3.57], 0.0%	0.31 [0.00; 1.14], 0.0%	1.64 [0.00; 8.01], 68.5%	10.86 [6.64; 15.87], 62.8%
Hypopituitarism	0.96 [0.31; 1.88], 23.4%	0.16 [0.00; 0.88], 4.2%	0.00 [0.00; 2.11]	1.25 [0.18; 2.95], 0.0%
Hypothyroidism	2.05 [0.53; 4.28], 74.7%	7.09 [4.16; 10.64], 59.4%	8.15 [4.81; 12.17], 0.0%	16.37 [13.07; 19.95], 6.4%
Lymphocytic hypophysitis	0.04 [0.00; 0.44], 0.0%	0.00 [0.00; 01.21]	NR	0.64 [0.18; 2.30]
Thyroiditis	0.42 [0.06; 1.01], 0.0%	1.63 [0.00; 4.96], 42.9%	NR	5.39 [0.04; 15.93], 73.6%
Thyrotoxic crisis	0.28 [0.05; 1.55]	NR	NR	NR
**Eye disorders**				
Uveitis	0.14 [0.00; 0.97], 52.8%	0.24 [0.00; 1.33], 0.0%	0.04 [0.00; 2.26], 0.0%	2.69 [0.01; 8.19], 72.1%
**Gastrointestinal disorders**				
Autoimmune colitis	1.38 [0.31; 2.99], 44.6%	0.64 [0.18; 2.30]	0.56 [0.10; 3.11]	1.41 [0.00; 4.98], 68.0%
Autoimmune pancreatitis	0.00 [0.00; 0.22], 0.0%	0.32 [0.06; 1.79]	NR	0.17 [0.00; 2.18], 62.6%
Colitis	6.87 [5.31; 8.58], 20.2%	1.88 [0.28; 4.41], 69.4%	1.64 [0.00; 8.01], 68.5%	11.80 [6.57; 18.16], 66.3%
Diarrhea	27.74 [24.28; 31.33], 46.0%	17.68 [12.07; 24.05], 76.4%	11.14 [6.56; 16.63], 32.9%	35.69 [26.65; 45.26], 72.7%
Enterocolitis	0.03 [0.00; 0.51], 0.0%	NR	0.00 [0.00; 2.11]	0.36 [0.00; 1.36], 0.0%
Frequent bowel movements	0.39 [0.07; 2.18]	0.97 [0.27; 3.47]	NR	NR
Gastrointestinal perforation	0.32 [0.02; 0.83], 0.0%	0.10 [0.00; 0.72], 16.3%	1.12 [0.31; 4.00]	0.63 [0.02; 1.79], 0.0%
Gastrointestinal toxicity	NR	8.70 [2.42; 26.80]	NR	NR
Pancreatitis	0.01 [0.00; 0.35], 6.0%	0.62 [0.07; 1.50], 0.0%	0.26 [0.00; 1.50], 0.0%	0.75 [0.05; 1.95], 0.0%
Peritonitis	0.13 [0.00; 0.80], 0.0%	0.32 [0.06; 1.79]	NR	0.32 [0.06; 1.79]
Rectal hemorrhage	0.86 [0.00; 3.19], 71.7%	NR	0.00 [0.00; 2.11]	0.36 [0.00; 1.36], 0.0%
Ulcerative colitis	0.00 [0.00; 1.05]	NR	NR	NR
**General disorders**				
Infusion-related reaction	0.75 [0.00; 2.51], 59.1%	2.95 [0.82; 6.00], 67.7%	0.00 [0.00; 2.11]	1.94 [0.45; 4.11], 0.0%
**Hepatobiliary disorders**				
Acute hepatic failure	0.13 [0.00; 1.55], 58.5%	NR	NR	NR
Acute hepatitis	0.00 [0.00; 0.29], 0.0%	NR	NR	0.32 [0.06; 1.79]
Autoimmune hepatitis	0.32 [0.02; 0.85], 0.0%	0.64 [0.18; 2.30]	0.22 [0.00; 2.22], 48.5%	1.62 [0.52; 3.18], 0.0%
Hepatitis	0.29 [0.00; 0.91], 20.6%	3.01 [0.00; 24.87], 94.2%	0.68 [0.00; 2.24], 0.0%	4.85 [0.44; 12.65], 87.3%
Hepatocellular injury	0.15 [0.00; 0.80], 32.1%	0.80 [0.14; 1.88], 18.8%	NR	0.89 [0.11; 2.17], 0.0%
Hepatotoxicity	0.42 [0.06; 1.01], 0.0%	0.78 [0.00; 7.85], 65.1%	NR	3.19 [1.74; 5.78]
Hyperbilirubinemia	0.13 [0.00; 0.80], 0.0%	0.32 [0.06; 1.79]	NR	2.24 [1.09; 4.54]
**Immune system disorders**				
Episcleritis	NR	NR	NR	NR
Hypersensitivity	0.43 [0.00; 1.58], 57.5%	2.00 [1.09; 3.12], 0.0%	0.56 [0.10; 3.11]	2.88 [1.52; 5.37]
**Infections**				
Aseptic meningitis	1.05 [0.19; 0.05.72]	0.00 [0.00; 1.21]	0.00 [0.00; 2.11]	0.32 [0.06; 1.79]
Conjunctivitis	0.40 [0.00; 2.37], 51.4%	NR	NR	NR
Encephalitis	0.04 [0.00; 0.38], 0.0%	NR	NR	0.32 [0.06; 1.79]
**Investigations**				
Alanine aminotransferase (increased)	2.40 [1.01; 4.23], 63.1%	2.58 [1.34; 4.12], 18.8%	3.98 [1.92; 6.59], 0.0%	18.65 [9.78; 29.43], 83.9%
Amylase (increased)	0.67 [0.00; 4.30], 86.7%	5.81 [3.49; 8.62], 0.0%	1.12 [0.20; 6.09]	9.31 [6.89; 12.01], 0.0%
Aspartate aminotransferase (increased)	2.54 [0.97; 4.66], 68.8%	2.84 [1.35; 4.73], 36.4%	4.49 [2.31; 7.21], 0.0%	17.82 [9.09; 28.51], 84.1%
Blood alkaline phosphatase (increased)	0.97 [0.26; 1.99], 30.5%	3.81 [0.62; 9.04], 83.2%	0.00 [0.00; 14.87]	5.53 [2.86; 8.91], 41.9%
Blood bilirubin (increased)	0.17 [0.00; 1.28], 65.1%	0.27 [0.00; 1.09], 0.0%	3.37 [1.55; 7.16]	3.21 [0.89; 6.59], 43.5%
Blood corticotropin (decreased)	0.38 [0.00; 3.07], 74.5%	NR	NR	NR
Blood creatinine (increased)	0.27 [0.00; 1.24], 57.0%	1.11 [0.04; 3.10], 48.7%	0.45 [0.00; 5.27], 65.7%	3.16 [1.53; 5.20], 0.0%
Blood thyroid stimulating hormone (abnormal)	0.94 [0.12; 2.26], 0.0%	0.23 [0.00; 1.66], 0.0%	1.12 [0.20; 6.09]	5.62 [0.00; 23.04], 73.4%
Blood urea (increased)	NR	NR	NR	NR
Gamma-glutamyl transferase (increased)	0.58 [0.00; 2.03], 50.4%	0.55 [0.00; 3.93], 52.1%	NR	2.62 [0.95; 4.85], 0.0%
Hepatic enzyme (increased)	0.00 [0.00; 0.29], 0.0%	0.34 [0.00; 1.07], 0.0%	0.00 [0.00; 2.11]	1.60 [0.68; 3.68]
Lipase (increased)	1.60 [0.00; 5.44], 88.4%	3.10 [0.00; 11.74], 85.4%	NR	14.31 [11.40; 17.46], 0.0%
Liver function test (abnormal)	0.00 [0.00; 0.09], 0.0%	0.64 [0.07; 1.63], 17.8%	NR	0.36 [0.00; 1.36], 0.0%
Thyroxine free (decreased)	NR	NR	1.12 [0.20; 6.09]	NR
Thyroxine (increased)	NR	NR	1.12 [0.20; 6.09]	NR
Transaminases (increased)	0.59 [0.07; 1.43], 0.0%	0.64 [0.18; 2.30]	0.00 [0.00; 14.87]	2.81 [1.45; 4.52], 0.0%
**Metabolism and nutrition disorders**				
Diabetes mellitus	0.51 [0.00; 10.95], 77.2%	0.44 [0.00; 1.35], 0.0%	2.38 [0.42; 12.32]	0.96 [0.33; 2.78]
Diabetic ketoacidosis	0.00 [0.00; 0.16], 0.0%	0.11 [0.00; 0.94], 35.4%	NR	0.36 [0.00; 1.36], 0.0%
Hyperglycemia	0.75 [0.00; 3.53], 82.5%	0.58 [0.10; 1.31], 0.0%	5.57 [2.77; 9.09], 0.0%	2.60 [1.19; 4.46], 0.0%
Hyperlipasemia	0.00 [0.00; 1.05]	NR	NR	NR
Type 1 diabetes mellitus	0.00 [0.00; 1.48]	NR	2.38 [0.42; 12.32]	1.06 [0.19; 5.78]
**Musculoskeletal and connective tissue disorders**				
Arthralgia	6.23 [2.95; 10.50], 85.0%	9.35 [5.66; 13.75], 67.3%	7.74 [4.47; 11.68], 0.0%	14.64 [11.70; 17.82], 0.0%
Arthritis	0.00 [0.00; 0.34], 0.0%	0.10 [0.00; 0.72], 16.3%	1.90 [0.00; 6.85], 41.4%	0.32 [0.06; 1.79]
Arthropathy	0.00 [0.00; 1.22]	0.32 [0.06; 1.79]	0.00 [0.00; 4.14]	0.64 [0.18; 2.30]
Hand-foot-syndrome (Palmar-plantar erythrodysesthesia syndrome)	0.97 [0.17; 5.30]	NR	NR	NR
Joint swelling	NR	NR	0.00 [0.00; 4.14]	NR
Muscle spasms	0.46 [0.03; 1.21], 0.0%	3.35 [0.84; 7.32], 78.7%	0.67 [0.00; 9.22], 65.9%	2.24 [1.09; 4.54]
Myalgia	3.24 [1.03; 6.36], 81.4%	4.19 [1.85; 7.24], 63.4%	4.85 [2.01; 8.59], 0.0%	11.90 [5.22; 20.66], 82.7%
Myopathy	NR	NR	NR	NR
Polymyalgia rheumatica	0.30 [0.00; 1.11], 0.0%	NR	NR	NR
Polymyositis/ myositis	0.10 [0.00; 0.75], 19.7%	0.32 [0.06; 1.79]	NR	2.13 [0.59; 7.43]
Rhabdomyolysis	0.00 [0.00; 3.89]	NR	NR	1.06 [0.19; 5.78]
**Nervous system disorders**				
Guillain-Barre syndrome	0.00 [0.00; 0.09], 0.0%	0.11 [0.00; 0.94], 35.4%	NR	0.36 [0.00; 1.36], 0.0%
Lethargy	0.20 [0.00; 1.25], 57.0%	NR	1.12 [0.20; 6.09]	0.32 [0.06; 1.79]
Myasthenia gravis	NR	NR	NR	NR
Myelitis	0.39 [0.07; 2.18]	NR	NR	NR
Neuropathy	0.63 [0.00; 10.45], 71.7%	NR	NR	0.64 [0.18; 2.30]
Peripheral neuropathy	0.43 [0.00; 1.80], 68.8%	2.36 [0.84; 4.47], 38.5%	0.40 [0.00; 2.33], 0.0%	3.53 [1.15; 6.89], 40.4%
**Renal and urinary disorders**				
Acute renal failure	0.31 [0.02; 0.80], 0.0%	0.04 [0.00; 0.44], 0.0%	0.26 [0.00; 1.50], 0.0%	0.52 [0.00; 1.79], 0.0%
Nephritis	0.15 [0.00; 0.59], 0.0%	0.12 [0.00; 0.76], 0.0%	0.56 [0.10; 3.11]	0.69 [0.00; 5.76], 60.2%
Nephrotoxicity	NR	0.00 [0.00; 7.35]	NR	0.32 [0.06; 1.79]
Renal failure	0.10 [0.00; 1.02], 60.9%	0.75 [0.21; 1.54], 0.0%	0.45 [0.00; 3.39], 0.0%	0.00 [0.00; 0.42], 0.0%
**Respiratory, thoracic and mediastinal disorders**				
Alveolitis	NR	NR	0.00 [0.00; 2.11]	NR
Lung infiltration	0.00 [0.00; 1.22]	NR	NR	0.32 [0.06; 1.79]
Pneumonitis	0.32 [0.00; 1.34], 60.4%	1.36 [0.61; 2.34], 0.0%	1.46 [0.10; 3.77], 0.0%	7.50 [5.06; 10.32], 10.1%
Pulmonary toxicity	NR	4.35 [0.77; 20.99]	NR	NR
**Skin and subcutaneous tissue disorders**				
Alopecia	1.67 [0.17; 4.18], 43.3%	1.95 [0.01; 6.02], 77%	3.37 [1.55; 7.16]	0.00 [0.00; 9.89]
Blister	NR	NR	NR	0.00 [0.00; 18.43]
Dermatitis	4.68 [0.00; 18.84], 97.0%	2.23 [1.14; 3.62], 0.0%	NR	1.92 [0.88; 4.12]
Eczema	0.11 [0.00; 0.85], 18.6%	2.10 [0.99; 3.57], 0.0%	4.48 [0.00; 22.04], 74.9%	2.88 [1.52; 5.37]
Erythema	1.63 [0.64; 2.96], 47.9%	4.00 [1.94; 6.68], 42.5%	4.74 [2.38; 7.72], 0.0%	4.79 [0.55; 12.34], 82.0%
Neutrophilic dermatosis	NR	NR	1.12 [0.20; 6.09]	NR
Night sweats	2.04 [0.14; 5.37], 0.0%	0.00 [0.00; 8.57]	1.12 [0.20; 6.09]	5.26 [2.51; 8.74], 0.0%
Pemphigoid	0.00 [0.00; 0.34], 0.0%	0.00 [0.00; 1.21]	NR	0.32 [0.06; 1.79]
Photosensitivity reaction	NR	3.84 [0.00; 19.48], 75.2%	NR	NR
Pruritus	24.88 [19.61; 30.53], 79.3%	22.53 [14.42; 31.78], 87.0%	21.40 [17.03; 26.10], 0.0%	33.73 [27.64; 40.10], 42.6%
Rash	20.54 [16.04; 25.42], 75.0%	18.10 [10.83; 26.65], 86.0%	13.47 [9.86; 17.50], 0.0%	30.68 [15.64; 48.07], 92.3%
Rash erythematous	1.13 [0.00; 4.24], 69.3%	1.12 [0.38; 3.24]	NR	1.06 [0.19; 5.78]
Rash generalized	1.26 [0.00; 4.73], 72.3%	0.64 [0.18; 2.30]	1.12 [0.20; 6.09]	2.56 [1.30; 4.96]
Rash macular	0.32 [0.06; 1.80]	0.64 [0.18; 2.30]	NR	2.34 [1.01; 4.13], 0.0%
Rash maculo-papular	5.26 [1.03; 12.10], 93.1%	16.08 [2.73; 36.78], 97.7%	3.89 [0.65; 8.97], 61.2%	20.29 [8.42; 35.55], 91.8%
Rash papular	1.26 [0.46; 2.37], 0.0%	1.68 [0.70; 3.03], 0.0%	1.12 [0.20; 6.09]	2.24 [1.09; 4.54]
Rash pruritic	2.41 [1.05; 4.21], 58.3%	0.32 [0.06; 1.79]	NR	1.84 [0.62; 3.55], 4.5%
Skin exfoliation	NR	NR	NR	NR
Skin hypopigmentation	0.22 [0.00; 1.23], 47.1%	2.24 [1.09; 4.54]	1.18 [0.00; 9.48], 0.0%	3.51 [0.34; 9.23], 76.2%
Skin reactions	NR	NR	NR	NR
Toxic skin eruption	0.00 [0.00; 0.42], 36.3%	0.32 [0.06; 1.79]	2.38 [0.42; 12.32]	NR
Urticaria	0.35 [0.00; 1.98], 0.0%	NR	9.52 [3.77; 22.07]	0.00 [0.00; 18.43]
Vitiligo	2.39 [0.79; 4.65], 63.7%	9.13 [5.06; 14.12], 73.3%	7.69 [4.89; 10.99], 0.0%	8.61 [6.06; 11.50], 8.0%
**Vascular disorders**				
Thromboembolic event	0.34 [0.00; 1.50], 63.0%	0.49 [0.07; 1.17], 0.0%	1.12 [0.31; 4.00]	0.79 [0.00; 3.46], 61.0%

*NR, not reported*.

**Table 5:** Incidence of grade ≥3 Potential Immune-Related Adverse Events (irAEs) in Advanced Melanoma presented as % with 95% confidence interval, and *I*^2^ for the % of heterogeneity for the FDA approved doses (*I*^2^ not reported if the outcome was obtained from one study).

**irAEs**	**Ipilimumab**	**Nivolumab**	**Pembrolizumab**	**Nivolumab +Ipilimumab**
Number of studies	10	7	4	5
Overall incidence	11.95 [10.17; 13.85], 7.0%	4.00 [1.00; 7.00], 70.0%	1.65 [0.33; 3.59], 0.0%	32.00 [16.00; 49.00], 93.0%
**Blood and lymphatic system disorders**				
Lymphopenia	0.00 [0.00; 1.80]	2.20 [0.00; 24.35], 87%	0.00 [0.0000; 0.0838]	NR
**Cardiac disorders**				
Pericarditis	0.00 [0.00; 1.05]	0.37 [0.07; 2.08]	NR	NR
Ventricular arrhythmia	0.00 [0.00; 7.71]	0.37 [0.07; 2.08]	NR	0.00 [0.00; 3.93]
**Endocrine disorders**				
Acute adrenocortical insufficiency	0.10 [0.00; 0.58], 0.0%	0.00 [0.00; 1.21]	0.00 [0.00; 2.11]	0.32 [0.06; 1.79]
Adrenal insufficiency	0.00 [0.00; 0.16], 0.0%	0.77 [0.07; 1.97], 25.2%	0.68 [0.00; 2.24], 0.0%	1.13 [0.22; 2.48], 0.0%
Adrenocorticotropic hormone deficiency	0.00 [0.00; 1.05]	NR	NR	NR
Autoimmune thyroiditis	0.00 [0.00; 0.18], 0.0%	0.00 [0.00; 1.21]	NR	0.36 [0.00; 1.36], 0.0%
Hyperthyroidism	0.04 [0.00; 0.38], 0.0%	0.00 [0.00; 0.22], 0.0%	0.00 [0.00; 0.61], 0.0%	0.52 [0.00; 1.73], 0.0%
Hypophysitis	1.21 [0.61; 1.97], 0.0%	0.15 [0.00; 0.87], 0.0%	0.68 [0.00; 3.10], 19.1%	2.48 [0.72; 4.97], 34.0%
Hypopituitarism	0.63 [0.07; 1.55], 36.0%	0.08 [0.00; 0.71], 0.0%	0.00 [0.00; 2.11]	0.43 [0.00; 1.71], 0.0%
Hypothyroidism	0.00 [0.00; 0.00], 0.0%	0.00 [0.00; 0.00], 0.0%	0.00 [0.00; 0.36], 0.0%	0.00 [0.00; 0.54], 0.0%
Lymphocytic hypophysitis	0.00 [0.00; 0.20], 0.0%	0.00 [0.00; 01.21]	NR	0.32 [0.06; 1.79]
Thyroiditis	0.00 [0.00; 0.22], 0.0%	0.00 [0.00; 0.15], 0%	NR	0.00 [0.00; 0.41], 0.0%
Thyrotoxic crisis	0.28 [0.05; 1.55]	NR	NR	NR
**Eye disorders**				
Uveitis	0.02 [0.00; 0.70], 49.4%	0.00 [0.00; 0.22], 0.0%	0.00 [0.00; 0.36], 0.0%	0.42 [0.00; 3.95], 70.1%
**Gastrointestinal disorders**				
Autoimmune colitis	0.81 [0.17; 1.75], 10.2%	0.32 [0.06; 1.79]	0.56 [0.10; 3.11]	1.19 [0.00; 5.54], 77.8%
Autoimmune pancreatitis	0.00 [0.00; 0.22], 0.0%	0.32 [0.06; 1.79]	NR	0.17 [0.00; 2.18], 62.6%
Colitis	4.69 [3.06; 6.59], 43.8%	0.33 [0.00; 1.00], 0.0%	0.34 [0.00; 4.92], 66.7%	9.02 [5.12; 13.76], 51.8%
Diarrhea	4.50 [3.03; 6.21], 37.5%	0.52 [0.00; 1.60], 30.6%	0.00 [0.00; 0.30], 0.0%	5.52 [2.24; 9.89], 58.4%
Enterocolitis	0.03 [0.00; 0.51], 0.0%	NR	0.00 [0.00; 2.11]	0.36 [0.00; 1.36], 0.0%
Frequent bowel movements	0.00 [0.00; 1.48]	0.00 [0.00; 1.83]	NR	NR
Gastrointestinal perforation	0.32 [0.02; 0.83], 0.0%	0.10 [0.00; 0.72], 16.3%	1.12 [0.31; 4.00]	0.63 [0.02; 1.79], 0.0%
Gastrointestinal toxicity	NR	4.35 [0.77; 20.99]	NR	NR
Pancreatitis	0.01 [0.00; 0.35], 6.0%	0.46 [0.02; 1.28], 0.0%	0.00 [0.00; 0.67], 0.0%	0.20 [0.00; 1.08], 0.0%
Peritonitis	0.13 [0.00; 0.80], 0.0%	0.32 [0.06; 1.79]	NR	0.32 [0.06; 1.79]
Rectal hemorrhage	0.10 [0.00; 0.65], 0.0%	NR	0.00 [0.00; 2.11]	0.14 [0.00; 0.95], 0.0%
Ulcerative colitis	0.00 [0.00; 1.05]	NR	NR	NR
**General disorders**				
Infusion-related reaction	0.00 [0.00; 0.33], 0.0%	0.00 [0.00; 0.17], 0.0%	0.00 [0.00; 2.11]	0.00 [0.00; 0.00], 0.0%
**Hepatobiliary disorders**				
Acute hepatic failure	0.13 [0.00; 1.55], 58.5%	NR	NR	NR
Acute hepatitis	0.00 [0.00; 0.29], 0.0%	NR	NR	0.32 [0.06; 1.79]
Autoimmune hepatitis	0.23 [0.00; 0.71], 0.0%	0.64 [0.18; 2.30]	0.22 [0.00; 2.22], 48.5%	1.62 [0.52; 3.18], 0.0%
Hepatitis	0.08 [0.00; 0.47], 0.0%	0.95 [0.00; 10.35], 85.5%	0.00 [0.00; 0.67], 0.0%	3.45 [0.32; 8.92], 80.5%
Hepatocellular injury	0.01 [0.00; 0.41], 22.7%	0.51 [0.04; 1.33], 0.0%	NR	0.63 [0.02; 1.79], 0.0%
Hepatotoxicity	0.24 [0.00; 0.77], 9.7%	0.00 [0.00; 0.61], 0.0%	NR	2.56 [1.30; 4.96]
Hyperbilirubinemia	0.13 [0.00; 0.80], 0.0%	0.00 [0.00; 1.21]	NR	0.00 [0.00; 1.21]
**Immune system disorders**				
Episcleritis	NR	NR	NR	NR
Hypersensitivity	0.00 [0.00; 0.07], 0.0%	0.11 [0.00; 0.59], 0.0%	0.00 [0.00; 2.11]	0.00 [0.00; 1.21]
**Infections**				
Aseptic meningitis	1.05 [0.19; 5.72]	0.00 [0.00; 1.21]	0.00 [0.00; 2.11]	0.32 [0.06; 1.79]
Conjunctivitis	0.00 [0.00; 0.11], 0.0%	NR	NR	NR
Encephalitis	0.04 [0.00; 0.38], 0.0%	NR	NR	0.32 [0.06; 1.79]
**Investigations**				
Alanine aminotransferase (increased)	0.27 [0.01; 0.75], 0.0%	0.38 [0.01; 1.09], 0.0%	0.00 [0.00; 0.30], 0.0%	8.94 [5.29; 13.32], 45.4%
Amylase (increased)	0.01 [0.00; 0.67], 35.3%	1.51 [0.34; 3.26], 0.0%	0.00 [0.00; 4.14]	3.12 [1.31; 5.48], 22.4%
Aspartate aminotransferase (increased)	0.11 [0.00; 0.58], 14.9%	0.12 [0.00; 0.67], 0.0%	0.00 [0.00; 0.30], 0.0%	7.01 [3.53; 11.38], 52.4%
Blood alkaline phosphatase (increased)	0.04 [0.00; 0.48], 17.9%	0.00 [0.00; 0.32]	0.00 [0.00; 14.87]	0.19 [0.00; 0.99], 0.0%
Blood bilirubin (increased)	0.00 [0.00; 0.01], 0.0%	0.00 [0.00; 0.16], 0.0%	0.00 [0.00; 2.11]	0.01 [0.00; 1.00], 29.1%
Blood corticotropin (decreased)	0.15 [0.00; 1.51], 47.6%	NR	NR	NR
Blood creatinine (increased)	0.00 [0.00; 0.01], 0.0%	0.00 [0.00; 0.25], 0.0%	0.00 [0.00; 0.36], 0.0%	0.13 [0.00; 1.45], 0.0%
Blood thyroid stimulating hormone (abnormal)	0.06 [0.00; 0.78], 0.0%	0.00 [0.00; 0.02], 0.0%	0.00 [0.00; 4.14]	0.00 [0.00; 1.02], 0.0%
Blood urea (increased)	NR	NR	NR	NR
Gamma-glutamyl transferase (increased)	0.03 [0.00; 0.48], 0.0%	0.00 [0.00; 0.22], 0.0%	NR	0.50 [0.00; 1.92], 0.0%
Hepatic enzyme (increased)	0.00 [0.00; 0.29], 0.0%	0.10 [0.00; 072], 16.3%	0.00 [0.00; 2.11]	0.96 [0.33; 2.78]
Lipase (increased)	0.29 [0.00; 2.29], 82.7%	2.22 [0.01; 6.83], 65.6%	NR	9.22 [6.81; 11.91], 0.0%
Liver function test (abnormal)	0.00 [0.00; 0.09], 0.0%	0.64 [0.07; 1.63], 17.8%	NR	0.36 [0.00; 1.36], 0.0%
Thyroxine free (decreased)	NR	NR	0.00 [0.00; 4.14]	NR
Thyroxine (increased)	NR	NR	0.00 [0.00; 4.14]	NR
Transaminases (increased)	0.00 [0.00; 0.34], 0.0%	0.32 [0.06; 1.79]	0.00 [0.00; 14.87]	2.44 [1.18; 4.08], 0.0%
**Metabolism and nutrition disorders**				
Diabetes mellitus	0.51 [0.00; 10.95], 77.2%	0.08 [0.00; 0.73], 0.0%	2.38 [0.42; 12.32]	0.64 [0.18; 2.30]
Diabetic ketoacidosis	0.00 [0.00; 0.16], 0.0%	0.11 [0.00; 0.94], 35.4%	NR	0.36 [0.00; 1.36], 0.0%
Hyperglycemia	0.00 [0.00; 0.29], 0.0%	0.44 [0.04; 1.12], 0.0%	0.99 [0.00; 4.29], 32.9%	1.61 [0.52; 3.18], 0.0%
Hyperlipasemia	0.00 [0.00; 1.05]	NR	NR	NR
Type 1 diabetes mellitus	0.00 [0.00; 1.48]	NR	2.38 [0.42; 12.32]	1.06 [0.19; 5.78]
**Musculoskeletal and connective tissue disorders**				
Arthralgia	0.29 [0.01; 0.82], 9.4%	0.00 [0.00; 0.04], 0.0%	0.04 [0.00; 1.27], 0.0%	0.00 [0.00; 0.52], 0.0%
Arthritis	0.00 [0.00; 0.34], 0.0%	0.10 [0.00; 0.72], 16.3%	0.00 [0.00; 1.03], 0.0%	0.32 [0.06; 1.79]
Arthropathy	0.00 [0.00; 1.22]	0.00 [0.00; 1.21]	0.00 [0.00; 4.14]	0.32 [0.06; 1.79]
Hand-foot-syndrome (Palmar-plantar erythrodysesthesia syndrome)	0.00 [0.00; 3.60]	NR	NR	NR
Joint swelling	NR	NR	0.00 [0.00; 4.14]	NR
Muscle spasms	0.00 [0.00; 0.19], 0.0%	0.00 [0.00; 0.33], 0.0%	0.00 [0.00; 1.26], 0.0%	0.64 [0.18; 2.30]
Myalgia	0.00 [0.00; 0.05], 0.0%	0.00 [0.00; 0.08], 0.0%	0.40 [0.00; 2.33], 0.0%	0.01 [0.00; 0.56], 0.0%
Myopathy	NR	NR	NR	NR
Polymyalgia rheumatica	0.13 [0.00; 0.80], 0.0%	NR	NR	NR
Polymyositis/ myositis	0.00 [0.00; 0.34], 0.0%	0.32 [0.06; 1.79]	NR	1.06 [0.19; 5.78]
Rhabdomyolysis	0.00 [0.00; 3.89]	NR	NR	1.06 [0.19; 5.78]
**Nervous system disorders**				
Guillain-Barre syndrome	0.00 [0.00; 0.09], 0.0%	0.11 [0.00; 0.94], 35.4%	NR	0.36 [0.00; 1.36], 0.0%
Lethargy	0.00 [0.00; 0.27], 0.0%	NR	0.00 [0.00; 4.14]	0.32 [0.06; 1.79]
Myasthenia gravis	NR	NR	NR	NR
Myelitis	0.39 [0.07; 2.18]	NR	NR	NR
Neuropathy	0.00 [0.00; 0.63], 0.0%	NR	NR	0.32 [0.06; 1.79]
Peripheral neuropathy	0.00 [0.00; 0.06], 0.0%	0.00 [0.00; 0.41], 0.0%	0.00 [0.00; 0.36], 0.0%	0.02 [0.00; 0.67], 0.0%
**Renal and urinary disorders**				
Acute renal failure	0.31 [0.02; 0.80], 0.0%	0.04 [0.00; 0.44], 0.0%	0.26 [0.00; 1.50], 0.0%	0.32 [0.00; 1.46], 0.0%
Nephritis	0.15 [0.00; 0.59], 0.0%	0.12 [0.00; 0.76], 0.0%	0.00 [0.00; 2.11]	0.03 [0.00; 1.57], 16.7%
Nephrotoxicity	NR	0.00 [0.00; 7.35]	NR	0.32 [0.06; 1.79]
Renal failure	0.05 [0.00; 0.76], 50.9%	0.30 [0.00; 0.89], 0.0%	0.00 [0.00; 1.26], 0.0%	0.00 [0.00; 0.42], 0.0%
**Respiratory, thoracic, and mediastinal disorders**				
Alveolitis	NR	NR	0.00 [0.00; 0.0211]	NR
Lung infiltration	0.00 [0.00; 1.22]	NR	NR	0.32 [0.06; 1.79]
Pneumonitis	0.07 [0.00; 0.44], 0.0%	0.00 [0.00; 0.25], 0.0%	0.27 [0.00; 1.85], 0.0%	0.81 [0.08; 2.01], 0.0%
Pulmonary toxicity	NR	0.00 [0.00; 14.31]	NR	NR
**Skin and subcutaneous tissue disorders**				
Alopecia	0.00 [0.00; 0.09], 0.0%	0.00 [0.00; 0.20], 0.0%	0.00 [0.00; 2.11]	0.00 [0.00; 9.89]
Blister	NR	NR	NR	0.00 [0.00; 18.43]
Dermatitis	0.00 [0.00; 0.26], 0.0%	0.00 [0.00; 0.33]. 0.0%	NR	0.00 [0.00; 1.21]
Eczema	0.00 [0.00; 0.02], 0.0%	0.00 [0.00; 0.36], 0.0%	0.00 [0.00; 2.84], 0.0%	0.00 [0.00; 121]
Erythema	0.00 [0.00; 0.17], 0.0%	0.00 [0.00; 0.23], 0.0%	0.00 [0.00; 0.32], 0.0%	0.14 [0.00; 0.95], 0.0%
Neutrophilic dermatosis	NR	NR	0.00 [0.00; 4.14]	NR
Night sweats	0.00 [0.00; 1.27], 0.0%	0.00 [0.00; 8.57]	0.00 [0.00; 4.14]	0.00 [0.00; 0.50],0.0%
Pemphigoid	0.00 [0.00; 0.34], 0.0%	0.00 [0.00; 1.21]	NR	0.32 [0.06; 1.79]
Photosensitivity reaction	NR	0.00 [0.00; 0.12], 0.0%	NR	NR
Pruritus	0.05 [0.00; 0.39], 0.0%	0.00 [0.00; 0.22], 0.0%	0.00 [0.00; 0.88], 21.9%	0.57 [0.01; 1.65], 0.0%
Rash	0.49 [0.10; 1.05], 0.0%	0.06 [0.00; 0.55], 0.0%	0.22 [0.00; 2.52], 51.3%	2.98 [1.22; 5.28], 21.5%
Rash erythematous	0.00 [0.00; 0.29], 0.0%	0.00 [0.00; 1.41]	NR	0.00 [0.00; 3.93]
Rash generalized	0.04 [0.00; 0.66], 0.0%	0.00 [0.00; 1.21]	0.00 [0.00; 4.14]	0.32 [0.06; 1.79]
Rash macular	0.32 [0.06; 1.80]	0.00 [0.00; 1.21]	NR	0.17 [0.00; 2.18], 0.0%
Rash maculo-papular	0.03 [0.00; 0.36], 0.0%	0.43 [0.00; 2.05], 66.3%	0.02 [0.00; 0.88], 0.0%	2.14 [0.00; 6.83], 81.0%
Rash papular	0.00 [0.00; 0.34], 0.0%	0.00 [0.00; 0.36],0.0%	0.00 [0.00; 4.14]	0.00 [0.00; 1.21]
Rash pruritic	0.00 [0.00; 0.22], 0.0%	0.00 [0.00; 1.21]	NR	0.00 [0.00; 0.38], 0.0%
Skin exfoliation	NR	NR	NR	NR
Skin hypopigmentation	0.00 [0.00; 0.34], 0.0%	0.00 [0.00; 1.21]	0.00 [0.00; 1.36], 0.0%	0.00 [0.00; 0.38], 0.0%
Skin reactions	NR	NR	NR	NR
Toxic skin eruption	0.00 [0.00; 0.42], 36.3%	0.32 [0.06; 1.79]	0.00 [0.00; 8.38]	NR
Urticaria	0.00 [0.00; 5.13]	NR	0.00 [0.00; 8.38]	0.00 [0.00; 18.43]
Vitiligo	0.00 [0.00; 0.14], 0.0%	0.00 [0.00; 0.03], 0.0%	0.00 [0.00; 0.30], 0.0%	0.00 [0.00; 0.04], 0.0%
**Vascular disorders**				
Thromboembolic event	0.19 [0.00; 0.91], 0.0%	0.49 [0.07; 1.17], 0.0%	0.00 [0.00; 2.11]	0.79 [0.00; 3.46], 61.0%

*NR, not reported*.

## Discussion

This systematic review and meta-analysis summarized the final evidence on irAEs reported in clinical trials of mono- and combination therapy of ipilimumab, nivolumab, and pembrolizumab in the treatment of advanced melanoma, and profiled the potential irAEs rates across these trials. The beneficial effects of using a combination of anti-CTLA-4 or anti-PD-1 agents were associated with an elevated incidence of potential irAEs. Anti-CTLA-4 therapy was mostly associated with an elevated incidence of potential irAEs of the gastrointestinal tract (colitis, diarrhea, enterocolitis, and gastrointestinal perforation), renal system (nephritis), skin (rash and pruritus), and endocrine system (hypophysitis and hypopituitarism). In contrast, the most frequent potential irAEs of anti-PD-1 therapies involved the endocrine system (hypothyroidism, hyperthyroidism, thyroiditis, and adrenal insufficiency), gastrointestinal tract (pancreatitis and peritonitis), hepatobiliary system (hepatitis, and hepatocellular injury), endocrine system (diabetes mellitus, diabetic ketoacidosis, hyperglycemia, and type 1 diabetes mellitus), musculoskeletal system (arthritis, myalgia, and myositis), nervous system (Guillain-Barre syndrome), renal system (acute renal failure, and renal failure), and respiratory system (pneumonitis).

Our finding that the combination of anti-CTLA-4 and anti-PD-1 therapies resulted in a frequent incidence of potential irAEs is consistent with the findings of three published reviews (63–65). The point estimates of the incidence of the potential irAEs in our study were slightly different from those obtained in previous meta-analyses because of different inclusion criteria; specifically, including phase1-3 ICI trials in advanced melanoma. Therefore, our analyses included a larger number of ICI studies and patients, and thus had broader evidence base and more statistical power. Furthermore, our-analysis took a step further by comprehensively profiling potential irAEs while previously reported meta-analyses were restricted to certain irAEs.

Furthermore, our findings of the most frequent incidence of potential irAEs after anti-CTLA-4 or anti-PD-1 monotherapies—especially the higher frequency of diarrhea, colitis, rash, pruritus, hypophysitis, hypothyroidism, hyperthyroidism, and pneumonitis—are similar to the findings of previous reviews and meta-analyses (11, 63, 64, 66–68). Previous studies showed that the irAE profile might vary between nivolumab and pembrolizumab. Hypothyroidism, colitis, and pneumonitis were more common after pembrolizumab (64, 69–71). We also found a similar pattern among pembrolizumab users.

In our analysis, we observed a trend of elevated incidence of diabetes among nivolumab users versus pembrolizumab users. After applying a pre-specified restriction of our analysis to the FDA approved doses, the incidence of diabetes was higher in pembrolizumab users. A prior meta-analysis of endocrine adverse events of ICIs across different types of cancers showed an elevated incidence of diabetes among nivolumab users versus among pembrolizumab users (70). Although nivolumab and pembrolizumab share a similar mechanism of action, the incidence of diabetes can be triggered by other factors such as the cancer types and the presence of genetic factors or autoimmune bodies. A recent meta-analysis by Lu et al. showed that the incidence of diabetes due to ICIs differs from one cancer type to another (72). Several studies have concluded that patients with an underlying genetic predisposition to diabetes or having autoimmune bodies such as antiglutamic acid decarboxylase 65 (GAD) antibody, are at risk of developing diabetes following treatment with anti-PD1 therapies (73–75). Moreover, subgroup analysis for the FDA doses reduced the number of studies as well as the sample of patients treated with ICIs, which may have affected the estimation of the risk of the incidence of diabetes (76).

Although a previous meta-analysis showed a trend of a high incidence of elevated levels of ALT and AST in melanoma patients treated with nivolumab versus pembrolizumab (69), our analyses revealed a higher incidence of these potential irAEs in patients treated with pembrolizumab. Note that our estimates of the incidence of elevated levels of ALT and AST was based on five pembrolizumab studies (1262 patients) and six nivolumab studies (996 patients), whereas the prior meta-analysis involved only two pembrolizumab studies (173 patients) and five nivolumab studies (955 patients). This differences in the number of studies and the higher aggregate of patients observed may explain the variation in the estimated incidence of these potential irAEs in our analysis compared to the previous meta-analysis (76).

Our findings are important for clinicians as they provide valuable information about the expected frequencies of potential irAEs in patients with advanced melanoma treated with anti-CTLA-4 or anti-PD-1 monotherapies or combination therapies. In the current study, the overall incidence of each potential irAE was presented across different doses as well as for the FDA-approved doses of ICIs. Moreover, the findings of our study could help clinicians including non-oncologists such as primary care providers be vigilant about serious potential irAEs and to raise their awareness about the expected incidence rates of potential irAEs. Also, our findings provide a safety reference of ICIs in advanced melanoma against which new data such as real-world data can be compared.

Our study has some strengths. To the best of our knowledge, the current meta-analysis is the first that was limited to advanced melanoma, included different combination of ICIs, and comprehensively presented the frequencies of potential irAEs for FDA-approved doses of ICIs. We used data from the regulatory bodies and clinicaltrials.gov to obtain more comprehensive data about potential irAEs, especially for trials that were not published as journal articles. Moreover, we included patients treated with ICI monotherapy or a combination of ICIs (anti-CTLA-4 and anti-PD-1) and excluded any combination of other active treatments to accurately estimate the incidence of potential irAEs.

This study nevertheless has some limitations. Classifying an adverse event as a potential irAE is very challenging and may vary across trials or observers, which may lead to detection bias because we included open-label trials as well as double-blind trials. These variations may lead to overestimating the incidence of potential irAEs. However, we included only patients treated with anti-CTLA-4 or anti-PD-1 monotherapies or combination therapies to avoid including AEs that could be related to other therapies or other immunotherapies. Moreover, we utilized the random-effect model for the meta-analyses as well as subgroup analyses for high heterogeneity to account for possible variation in the outcomes of the included studies (77, 78). In addition, the use of trial-level data rather than patient-level data may have introduced bias in estimating the incidence of potential irAEs owing to the variation in patient characteristics, which may be associated with the occurrence of potential irAEs. While we agree that identifying modifiers from patient-level data tends to be more robust than using trial-level data, we performed subgroup analyses to explore heterogeneity based on the available information on possible modifiers reported at the trial level (79).

## Conclusions

Overall, we found that the incidence of potential irAEs was high after using a combination of anti-CTLA-4 and anti-PD-1 therapies. Ipilimumab users tended to be at a higher risk of developing diarrhea, colitis, rash, pruritus, and hypophysitis than anti-PD-1 users. Pembrolizumab users were at a high risk of potential irAEs including thyroid disorders (hypothyroidism and hypothyroidism), endocrine disorders (hyperglycemia, and type 1 diabetes mellitus), respiratory disorders (pneumonitis), skin disorders (eczema, toxic skin eruptions, urticaria, and vitiligo), and musculoskeletal disorders (myalgia and arthralgia). Nivolumab users were likely to have a high incidence of maculopapular rash, erythema, hepatitis, infusion-related reaction, hypersensitivity, thyroiditis, elevated levels of amylase and lipase, muscle spasms, peripheral neuropathy, and renal failure. Accordingly, clinicians should be cognizant of variations in the potential irAE profiles of ICIs and weigh the benefits and risks of irAEs associated with these agents as mono or combination therapies. Nevertheless, further studies are needed to identify key predictors associated with the occurrence of irAEs as well as to compare their incidences in the real-world setting to help clinicians personalize treatment for patients and effectively manage any potential irAEs.

## Data Availability Statement

All the analyzed data in this study were included in the published article.

## Author Contributions

All authors: study concept and design, critical revision of the manuscript, and interpretation of data. AA: data acquisition and management and statistical analysis. AA and IA: drafting of the manuscript. IA: study supervision.

### Conflict of Interest

The authors declare that the research was conducted in the absence of any commercial or financial relationships that could be construed as a potential conflict of interest.

## Footnotes

^1^Study of Nivolumab Given Sequentially With Ipilimumab in Subjects With Advanced or Metastatic Melanoma (CheckMate 064). Available online at: https://ClinicalTrials.gov/show/NCT01783938

^2^Study of Nivolumab (BMS-936558) Plus Ipilimumab Compared With Ipilimumab Alone in the Treatment of Previously Untreated, Unresectable, or Metastatic Melanoma. Available online at: https://ClinicalTrials.gov/show/NCT01927419.

^3^Study of Pembrolizumab (MK-3475) in Participants With Advanced Melanoma (MK-3475–041/KEYNOTE-041). Available online at: https://ClinicalTrials.gov/show/NCT02180061

^4^Study to Evaluate the Safety and Efficacy of Two Different Dosing Schedules of Pembrolizumab (MK-3475) Compared to Ipilimumab in Participants With Advanced Melanoma (MK-3475–006/KEYNOTE-006). Available online at: https://ClinicalTrials.gov/show/NCT01866319

^5^Study of Pembrolizumab (MK-3475) Versus Chemotherapy in Participants With Advanced Melanoma (P08719/KEYNOTE-002). Available online at: https://ClinicalTrials.gov/show/NCT01704287

^6^A Study to Compare BMS-936558 to the Physician's Choice of Either Dacarbazine or Carboplatin and Paclitaxel in Advanced Melanoma Patients That Have Progressed Following Anti-CTLA-4 Therapy (CheckMate 037). Available online at: https://ClinicalTrials.gov/show/NCT01721746

^7^Study of Nivolumab (BMS-936558) Compared With Dacarbazine in Untreated, Unresectable, or Metastatic Melanoma. Available online at: https://ClinicalTrials.gov/show/NCT01721772

^8^Evaluation of Tumor Response to Ipilimumab in the Treatment of Melanoma With Brain Metastases. Available online at: https://ClinicalTrials.gov/show/NCT00623766

^9^Ipilimumab With or Without Talimogene Laherparepvec in Unresected Melanoma. Available online at: https://ClinicalTrials.gov/show/NCT01740297

^10^Phase 3 Trial in Subjects With Metastatic Melanoma Comparing 3 mg/kg Ipilimumab Versus 10 mg/kg Ipilimumab. Available online at: https://ClinicalTrials.gov/show/NCT01515189

^11^Drug-Drug Interaction - 3 Arm – Carboplatin/Paclitaxel, Dacarbazine. Available online at: https://ClinicalTrials.gov/show/NCT00796991

^12^Comparison of Ipilimumab Manufactured by 2 Different Processes in Participants With Advanced Melanoma. Available online at: https://ClinicalTrials.gov/show/NCT00920907

^13^MDX-010 Antibody, MDX-1379 Melanoma Vaccine, or MDX-010/MDX-1379 Combination Treatment for Patients With Unresectable or Metastatic Melanoma. Available online at: https://ClinicalTrials.gov/show/NCT00094653

^14^Phase 2 Study of Ipilimumab in Japanese Advanced Melanoma Patients. Available online at: https://ClinicalTrials.gov/show/NCT01990859

^15^Ipilimumab With or Without Sargramostim in Treating Patients With Stage III or Stage IV Melanoma That Cannot Be Removed by Surgery. Available online at: https://ClinicalTrials.gov/show/NCT01134614

^16^Goods Therapeutic Administration. Australian Public Assessment Report for Ipilimumab 2011. Available online at: https://www.tga.gov.au/sites/default/files/auspar-yervoy.pdf.

^17^Pharmaceutical and Mdical Devices Agency. Report on Deliberation Results for Ipilimumab 2015. Available online at: https://www.pmda.go.jp/files/000215223.pdf
