# Supplementary material for: Potential Immune-Related Adverse Events Associated With Monotherapy and Combination Therapy of Ipilimumab, Nivolumab, and Pembrolizumab for Advanced Melanoma: A Systematic Review and Meta-Analysis
Source: Front Oncol. 2020 Feb 11;10:91. doi: 10.3389/fonc.2020.00091 (PMC7033582; doi:10.3389/fonc.2020.00091)

**Supplementary Material**

# **Table S1: Search Strategies**

| **Medline-PubMed** |
| --- |
| ((("pembrolizumab"[ALL] OR "pembrolizumab"[Supplementary Concept] OR "keytruda "[ALL] OR "lambrolizumab"[ALL] OR "MK-3475"[ALL] OR "MK3475"[ALL] OR "MK 3475"[ALL] OR "SCH-900475"[ALL] OR "SCH900475"[ALL] OR "SCH 900475"[ALL] OR "nivolumab"[ALL] OR "nivolumab"[Supplementary Concept] OR "opdivo"[ALL] OR "BMS-936558"[ALL] OR "BMS936558"[ALL] OR "BMS 936558"[ALL] OR "ONO-4538"[ALL] OR "ONO4538"[ALL] OR "ONO 4538"[ALL] OR "MDX-1106"[ALL] OR "MDX1106"[ALL] OR "MDX 1106"[ALL] OR "immune checkpoint inhibitor*"[ALL] OR "anti-PD-1"[ALL] OR "anti-PD1"[ALL] OR "anti-PD 1"[ALL] OR "pd1 inhibitor*"[ALL] OR "pd 1 inhibitor*"[ALL] OR "pd 1 inhibitor nivolumab"[ALL] OR "programmed cell death 1 receptor/antagonists and inhibitors"[MeSH Terms] OR "programmed cell death 1 receptor/antagonists and inhibitors"[ALL] OR "programmed cell death 1 receptor antagonist*"[ALL] OR "programmed cell death 1 receptor inhibitor*"[ALL] OR "Ipilimumab"[ALL] OR "Ipilimumab "[Supplementary Concept] OR "MDX-010"[ALL] OR "MDX010"[ALL] OR "MDX 010"[ALL] OR "MDX-CTLA-4"[ALL] OR "MDX-CTLA4"[ALL] OR "BMS-734016"[ALL] OR "BMS734016"[ALL] OR "BMS 734016"[ALL] OR "Yervoy"[ALL] OR "anti CTLA4"[ALL] OR "anti CTLA-4"[ALL] OR "cytotoxic T-lymphocyte-associated protein-4"[ALL] OR "anti-cytotoxic T-lymphocyte-associated antigen-4"[ALL] OR "MOAB CTLA-4"[ALL] OR "monoclonal antibody CTLA-4"[ALL] OR "monoclonal antibody CTLA4"[ALL]))) AND (("melanoma"[MeSH]) OR "malignant melanoma*"[ALL] OR "melanoma*"[ALL] OR "advanced melanoma*"[ALL] OR "unresectable melanoma*"[ALL])) |
| **Cochrane Library-Trial** |
| #1 MeSH descriptor: [Melanoma] explode all trees  #2 Melanoma  #3 malignant melanoma  #4 advanced melanoma  #5 unresectable melanoma  #6 #1 OR #2 OR #3 OR #4 OR #5  #7 MeSH descriptor: [Ipilimumab] explode all trees  #8 Ipilimumab  #9 Anti-CTLA-4 MAb  #10 Anti CTLA 4 MAb  #11 MDX-CTLA-4  #12 MDX CTLA 4  #13 MDX-010  #14 MDX 010  #15 MDX010  #16 Yervoy  #17 BMS-734016  #18 BMS 734016  #19 anti CTLA4  #20 anti CTLA-4  #21 cytotoxic T-lymphocyte-associated protein-4  #22 anti-cytotoxic T-lymphocyte-associated antigen-4  #23 monoclonal antibody CTLA-4  #24 monoclonal antibody CTLA 4  #25 monoclonal antibody CTLA4  #26 #7 OR #8 OR #9 OR #10 OR #11 OR #12 OR #13 OR #14 OR #15 OR #16 OR #17 OR #18 OR #19 OR #20 OR #21 OR #22 OR #23 OR #24 OR #25  #27 pembrolizumab  #28 keytruda  #29 lambrolizumab  #30 MK-3475  #31 MK3475  #32 MK 3475  #33 SCH-900475  #34 SCH900475  #35 SCH 900475  #36 nivolumab  #37 opdivo  #38 BMS-936558  #39 BMS936558  #40 BMS 936558  #41 ONO-4538  #42 ONO4538  #43 ONO 4538  #44 MDX-1106  #45 MDX1106  #46 MDX 1106  #47 immune checkpoint inhibitor*  #48 anti-PD-1  #49 anti-PD1  #50 anti-PD 1  #51 programmed cell death 1 receptor antagonists  #52 programmed cell death 1 receptor inhibitors  #53 programmed cell death-1 receptor antagonists  #54 programmed cell death-1 receptor inhibitors  #55 #27 OR #28 OR #29 OR #30 OR #31 OR #32 OR #33 OR #34 OR #35 OR #36 OR #37 OR #38 OR #39 OR #40 OR #41 OR #42 OR #43 OR #44 OR #45 OR #46 OR #47 OR #48 OR #49 OR #50 OR #51 OR #52 OR #53 OR #54  #56 #26 OR # 55  #57 #6 AND #56  #7 OR #8 OR #9 OR #10 OR #11 OR #12 OR #13 OR #14 OR #15 OR #16 OR #17 OR #18 OR #19 OR #20 OR #21 OR #22 OR #23 OR #24 OR #25 OR #26 OR #27 OR #28 OR #29 OR #30 OR #31 OR #32 OR #33 OR #34 OR #35 OR #36 OR #37 OR #38 OR #39 OR #40 OR #41 OR #42 OR #43 OR #44 OR #45 OR #46 OR #47 OR #48 OR #49 OR #50 OR #51 OR #52 OR #53 OR #54 OR #55 OR #56 OR #57 |
| **Embase** |
| #1 'melanoma'/exp  #2 'advanced melanoma'/exp  #3 'advanced melanoma'  #4 'melanoma'  #5 'malignant melanoma*' OR 'unresectable melanoma*'  #6 #1 OR #2 OR #3 OR #4 OR #5  #7 'pembrolizumab'/exp  #8 pembrolizumab  #9 keytruda  #10 'lambrolizumab'/exp  #11 'lambrolizumab'  #12 'mk-3475' OR 'mk3475' OR 'mk 3475' OR 'sch-900475' OR 'sch900475' OR 'sch 900475'  #13 'nivolumab'/exp  #14 'nivolumab'  #15 opdivo  #16 'bms-936558' OR 'bms936558' OR 'bms 936558' OR 'ono-4538' OR 'ono4538' OR 'ono 4538' OR 'mdx-1106' OR 'mdx1106' OR 'mdx 1106'  #17 'immune checkpoint inhibitor*'  #18 'anti-pd-1' OR 'anti-pd1' OR 'anti-pd 1' OR 'pd1 inhibitor*' OR 'pd 1 inhibitor*' OR 'pd-1 inhibitor'  #19 'programmed cell death 1 receptor antagonist*'  #20 'programmed cell death-1 receptor antagonist*'  #21 'programmed cell death-1 receptor inhibitor*'  #22 'programmed cell death 1 receptor inhibitor*'  #23 'ipilimumab'/exp  #24 'ipilimumab'  #25 yervoy  #26 'mdx-010' OR 'mdx010' OR 'mdx 010' OR 'mdx-ctla-4' OR 'mdx-ctla4' OR 'bms-734016' OR 'bms734016' OR 'bms 734016'  #27 'anti ctla4' OR 'anti ctla-4' OR 'cytotoxic t-lymphocyte-associated protein-4' OR 'anti-cytotoxic t-lymphocyte-associated antigen-4'OR 'moab ctla-4' OR 'monoclonal antibody ctla-4' OR 'monoclonal antibody ctla4'  #28 #7 OR #8 OR #9 OR #10 OR #11 OR #12 OR #13 OR #14 OR #15 OR #16 OR #17 OR #18 OR #19 OR #20 OR #21 OR #22 OR #23 OR #24 OR #25 OR #26 OR #27  #29 #6 AND #28 |

# **Table S 2.**Quality assessment of non-randomized studies using Newcastle‐Ottawa quality scale for cohort studies

| **Study** | **Selection** | | | | **Comparability** | | **Outcome assessment** | | | **Total NOS assessment** |
| --- | --- | --- | --- | --- | --- | --- | --- | --- | --- | --- |
|  | **Representativeness of the exposed cohort** | **Selection of the non-exposed cohort** | **Ascertainment of exposure** | **Outcome of interest was not present at start of study** | **Controls for the most important factors** | **Controls for additional factor** | **Assessment of outcome** | **Enough follow-up for outcomes to occur** | **Adequacy of follow up of cohorts** |  |
| Callahan, 2017 | * | NA | * | * | NA | NA | Open-label | * | * | 5 |
| Day, 2010 | * | NA | * | * | NA | NA | Open-label | * | * | 5 |
| Downey, 2007 | * | * | * | * | NA | NA | Open-label | * | * | 6 |
| Goldberg,2016    **Goldberg,** | * | NA | * | * | NA | NA | Open-label | * | * | 5 |
| Haag, 2018 | * | NA | * | * | NA | NA | Open-label | * | * | 5 |
| Long, 2017 | * | NA | * | * | NA | NA | Open-label | * | * | 5 |
| Margolin, 2012 | * | NA | * | * | NA | NA | Open-label | * | * | 5 |
| Marker, 2006 | * | NA | * | * | NA | NA | Open-label | * | * | 5 |
| Tawbi, 2018 | * | NA | * | * | NA | NA | Open-label | * | * | 5 |
| Topalian, 2014 | * | NA | * | * | NA | NA | Open-label | * | * | 5 |
| Weber, 2008 | * | NA | * | * | NA | NA | Open-label | * | * | 5 |
| Weber, 2013 | * | NA | * | * | NA | NA | Open-label | * | * | 5 |
| Yamazaki, 2015 | * | NA | * | * | NA | NA | Open-label | * | * | 5 |
| Yamazaki, 2017 (JapicCTI-142533) | * | NA | * | * | NA | NA | Open-label | * | * | 5 |
| Yamazaki, 2017 (KEYNOTE-041) | * | NA | * | * | NA | NA | Open-label | * | * | 5 |
| Zimmer, 2015 | * | NA | * | * | NA | NA | Open-label | * | * | 5 |

NOS, Newcastle‐Ottawa quality scale; one “*” means one point

**Figure S 1:** Funnel plot of incidence of all-grade overall potential irAEs in ipilimumab
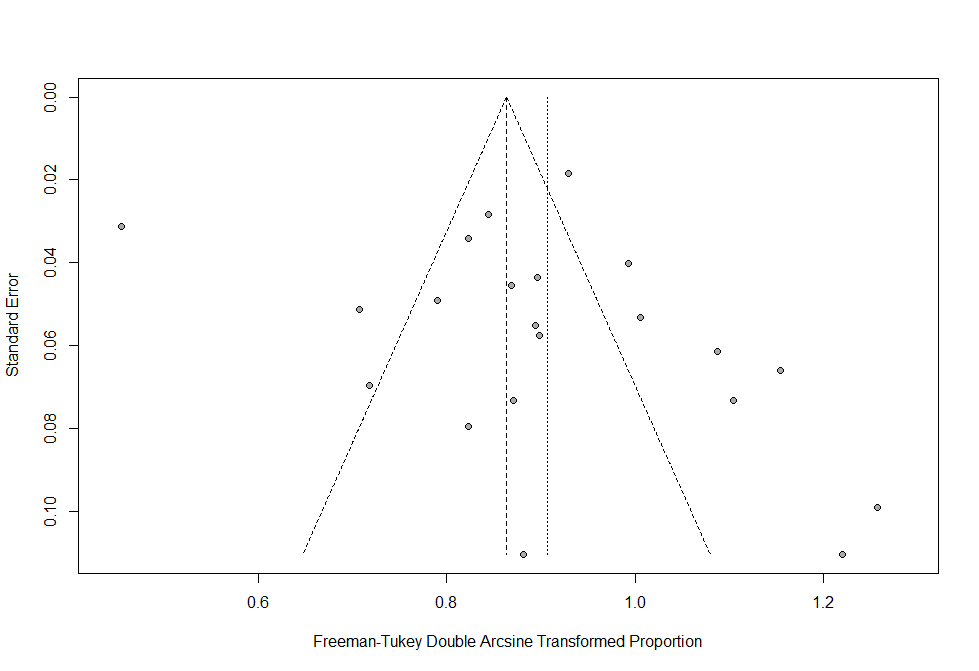


**Egger’s test:** p-value 0.31

**Figure S 2:** Funnel plot of incidence of grade≥3 overall potential irAEs in ipilimumab


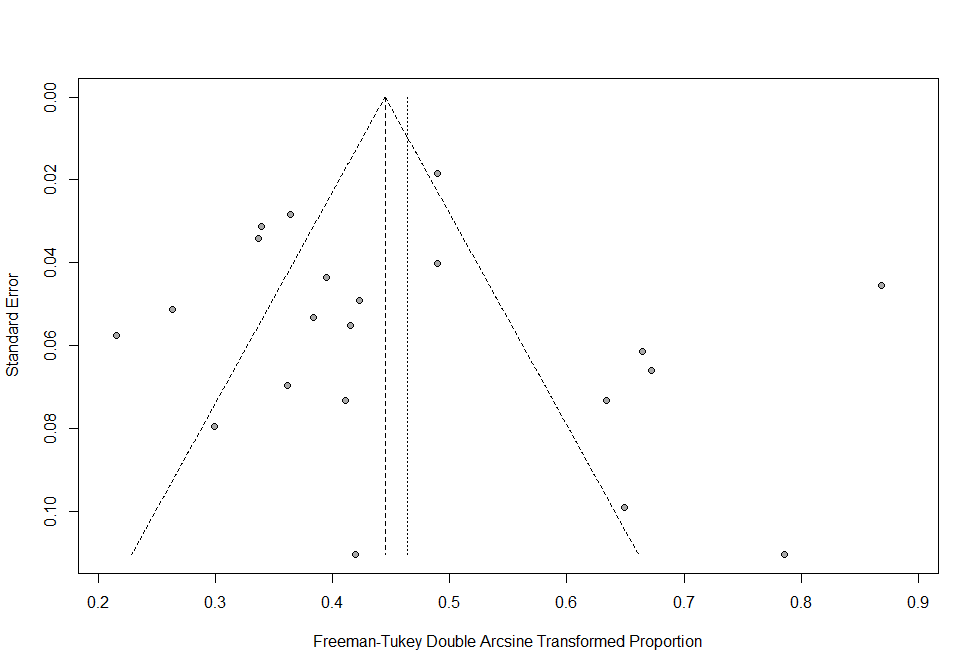


**Egger’s test:** p-value 0.59

**Figure S 3:** Funnel plot of incidence of all-grade overall potential irAEs in ipilimumab (FDA approved dose)


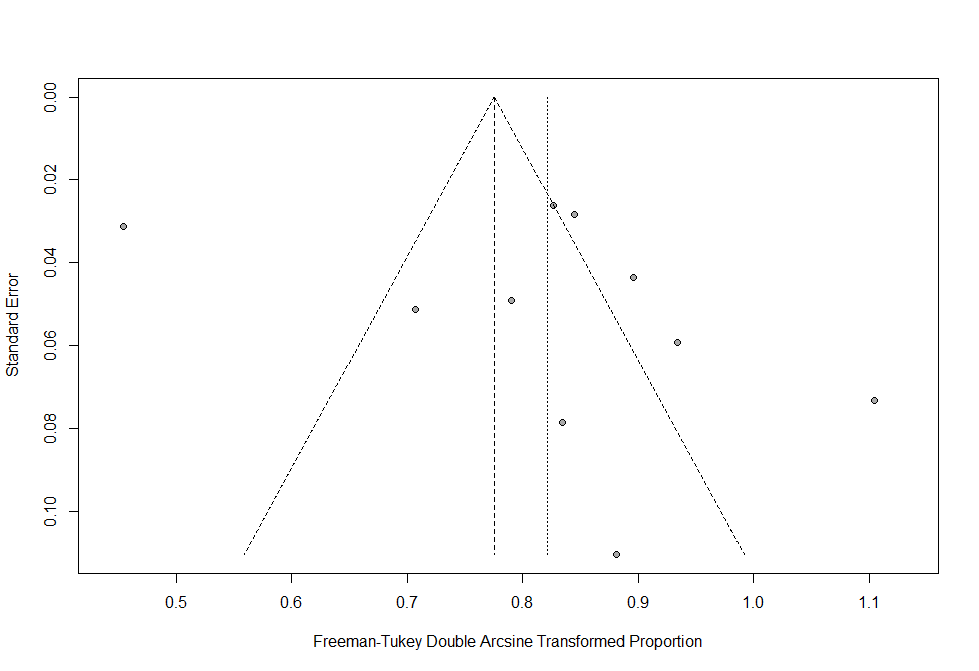


**Egger’s test:** p-value 0.39

**Figure S 4:** Funnel plot of incidence of grade≥3 overall potential irAEs in ipilimumab (FDA approved dose)


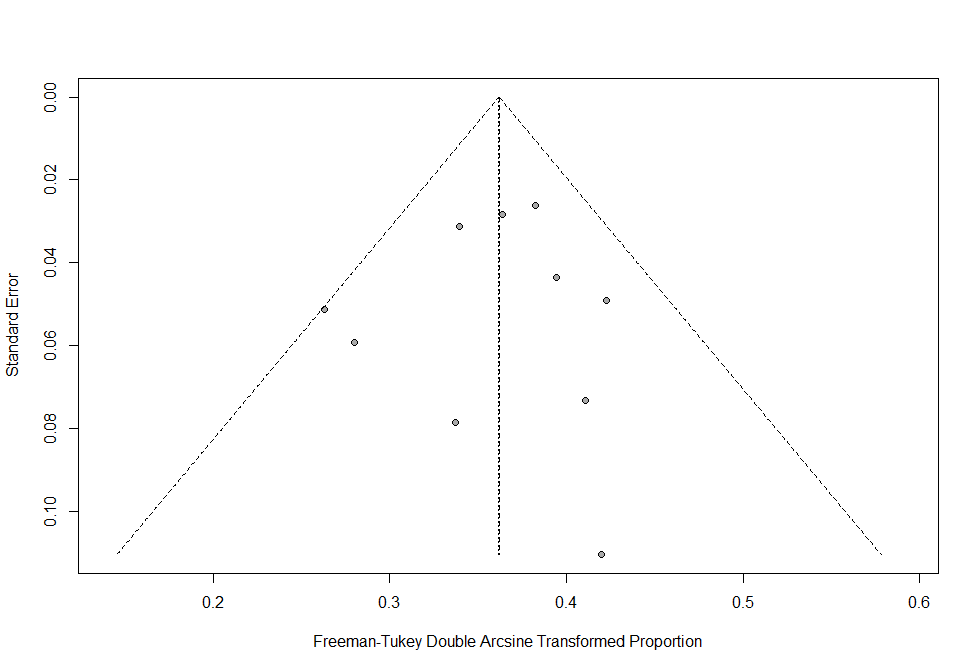


**Egger’s test:** p-value 0.82

**Figure S 5:** Funnel plot of incidence of all-grade overall potential irAEs in nivolumab
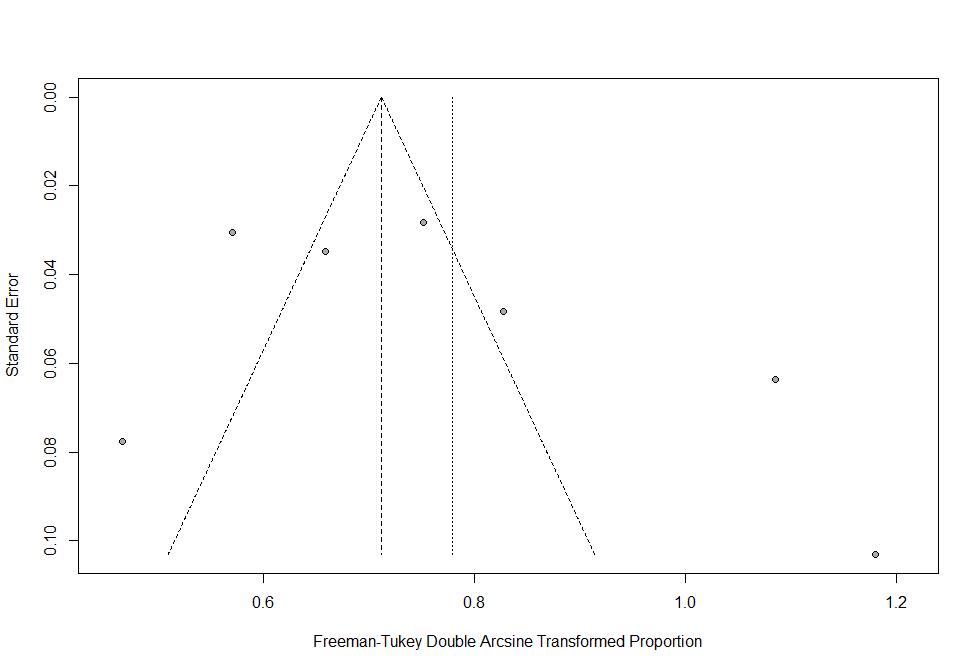


**Egger’s test:** p-value 0.27

**Figure S 6:** Funnel plot of incidence of grade≥3 overall potential irAEs in nivolumab


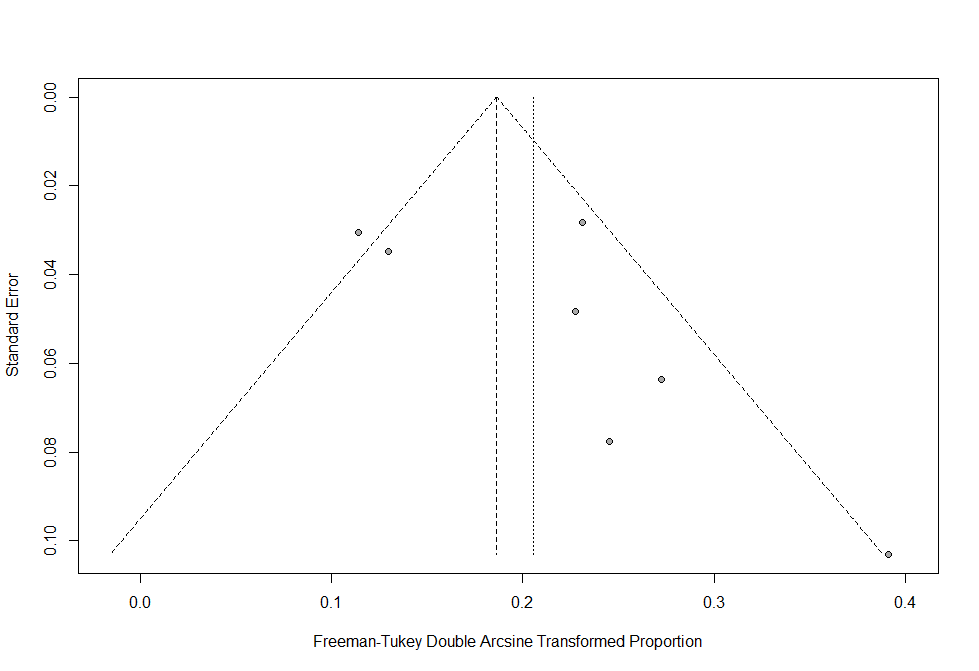


**Egger’s test:** p-value 0.16

**Figure S 7:** Funnel plot of incidence of all-grade overall potential irAEs in nivolumab (FDA approved dose)


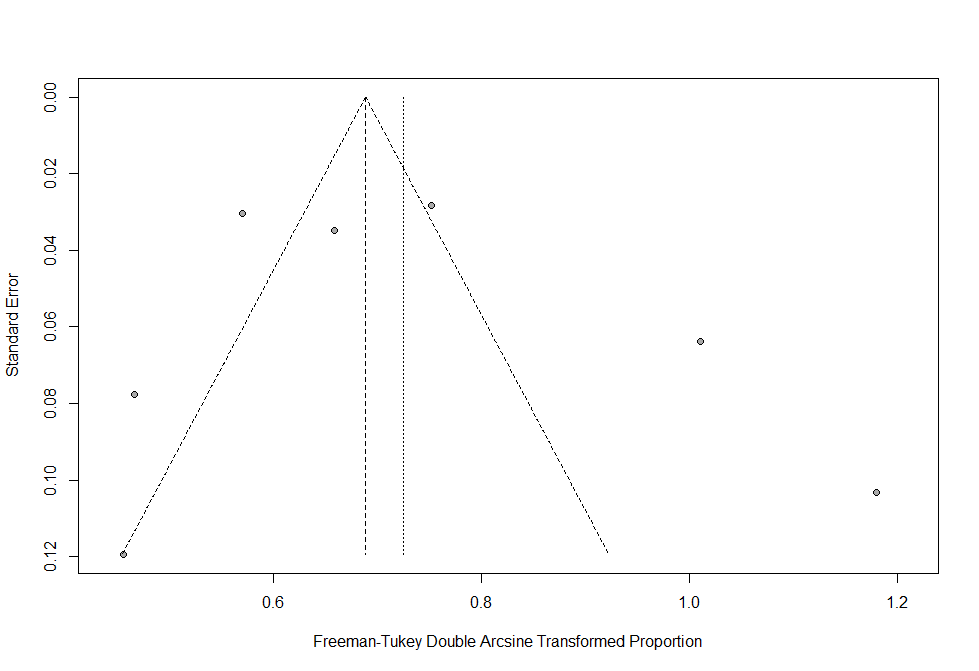


**Egger’s test:** p-value 0.62

**Figure S 8:** Funnel plot of incidence of grade≥3 overall potential irAEs in nivolumab (FDA approved dose)


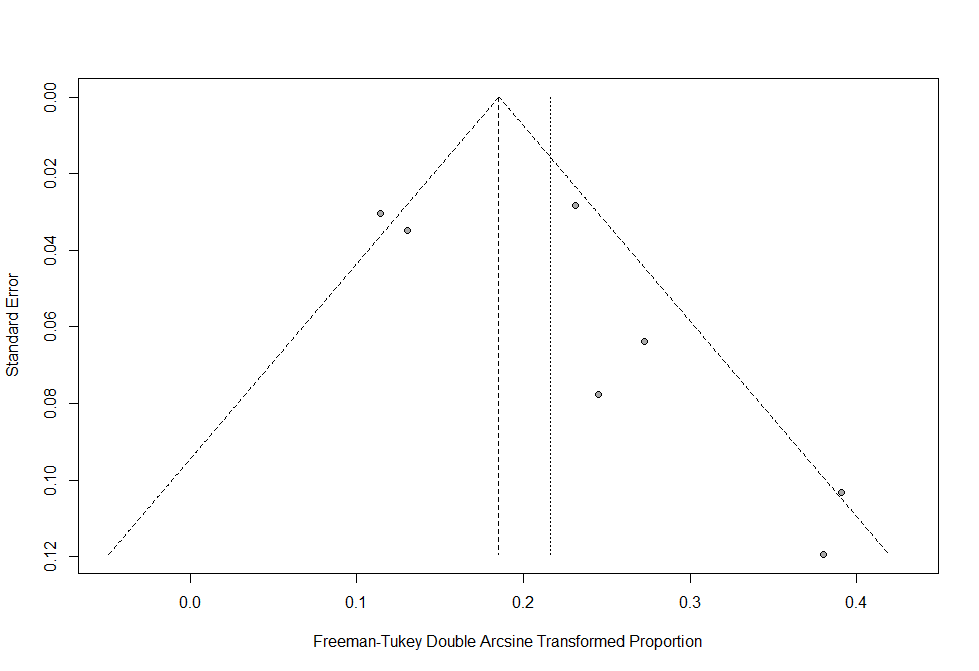


**Egger’s test:** p-value 0.12

**Figure S 9:** Funnel plot of incidence of all-grade overall potential irAEs in pembrolizumab


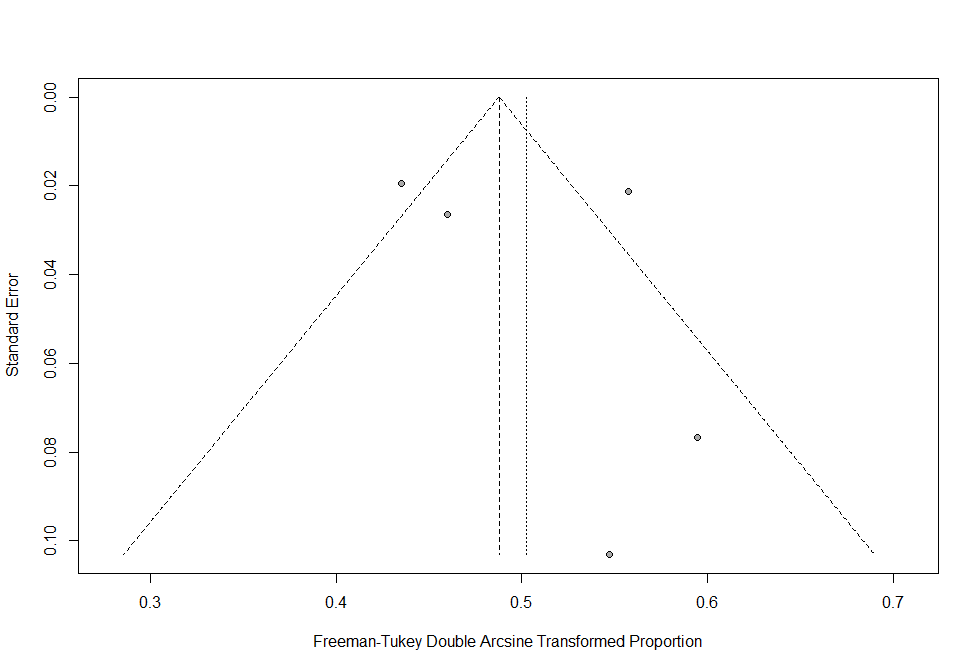


**Egger’s test:** p-value 0.62

**Figure S 10:** Funnel plot of incidence of grade≥3 overall potential irAEs in Pembrolizumab


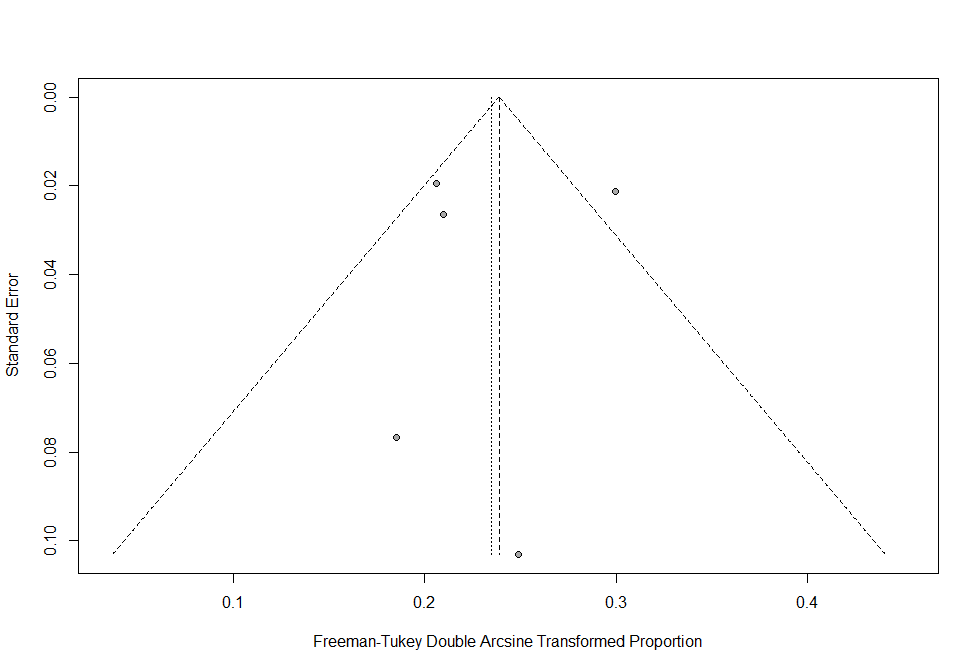


**Egger’s test:** p-value 0.83

**Figure S 11:** Funnel plot of incidence of all-grade overall potential irAEs in pembrolizumab (FDA approved dose)


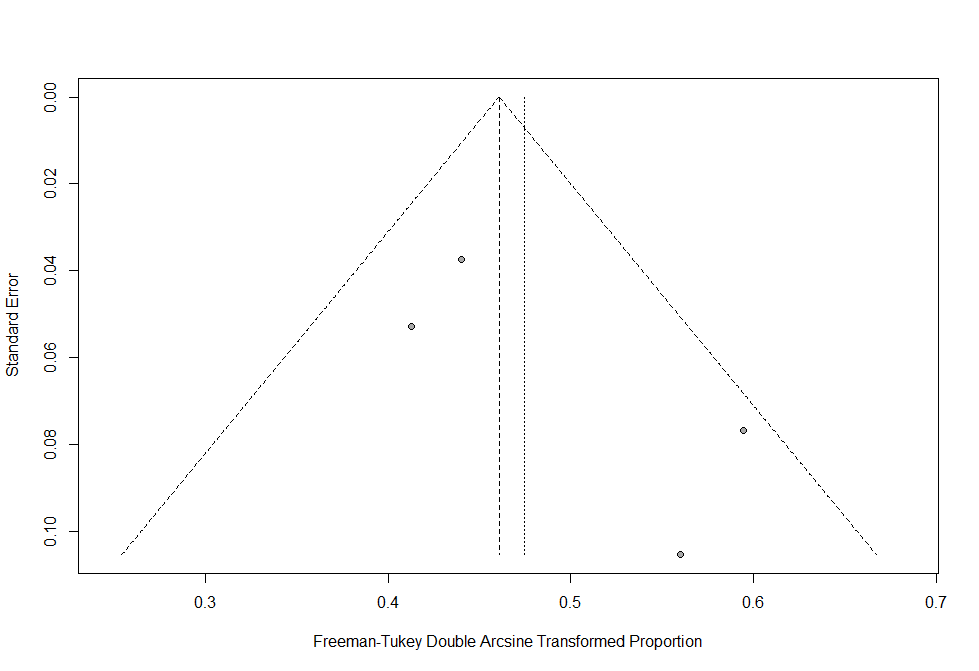


**Egger’s test:** p-value 0.23

**Figure S 12:** Funnel plot of incidence of grade≥3 overall potential irAEs in pembrolizumab (FDA approved dose)


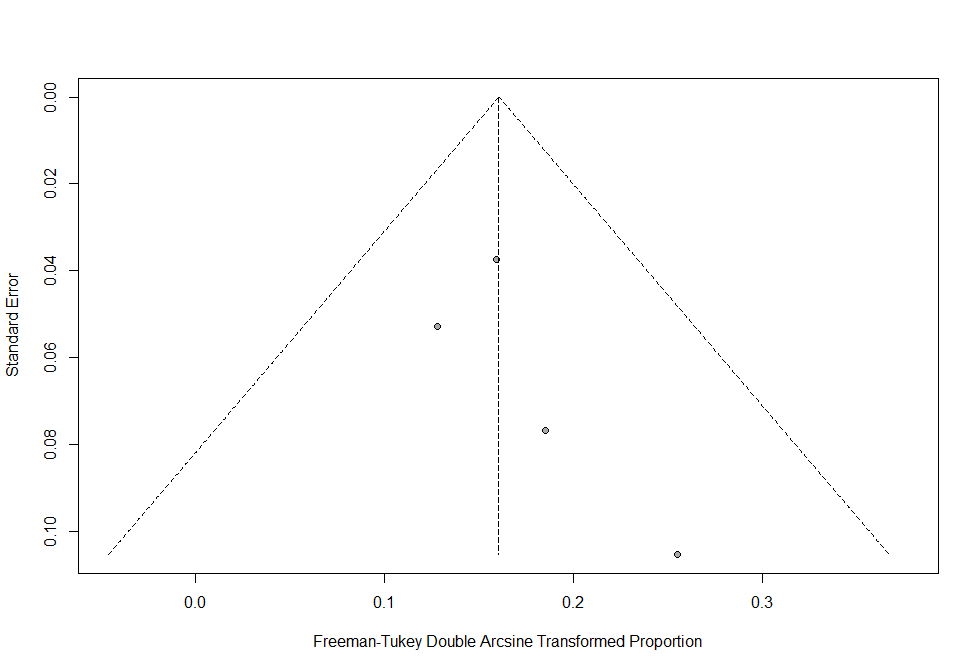


**Egger’s test:** p-value 0.31

**Figure S 13:** Funnel plot of incidence of all-grade overall potential irAEs in nivolumab and ipilimumab combination
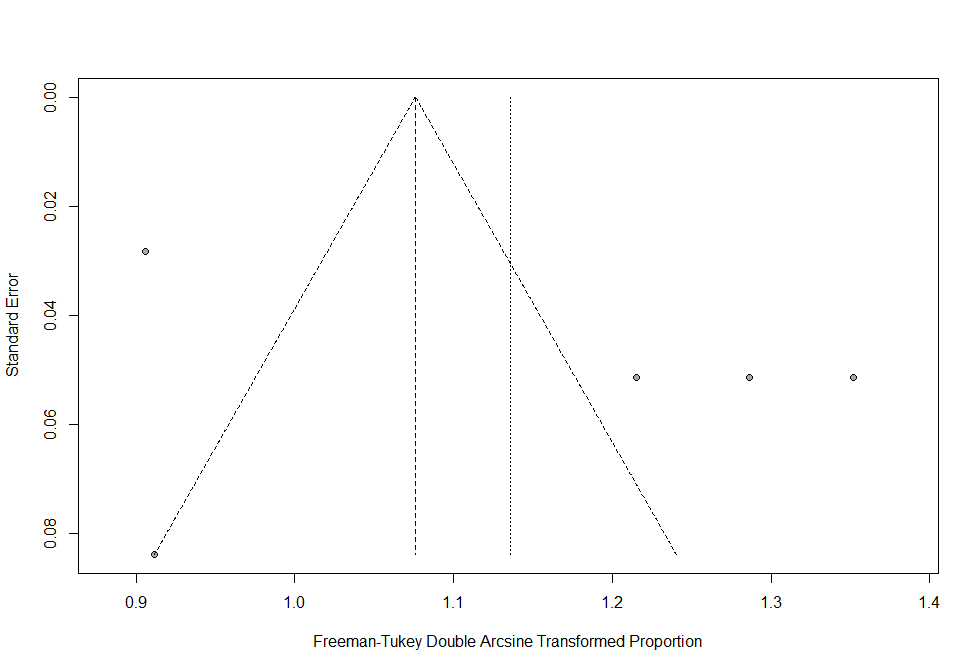


**Egger’s test:** p-value 0.32

**Figure S 14:** Funnel plot of incidence of grade≥3 overall potential irAEs in Overall nivolumab and ipilimumab combination
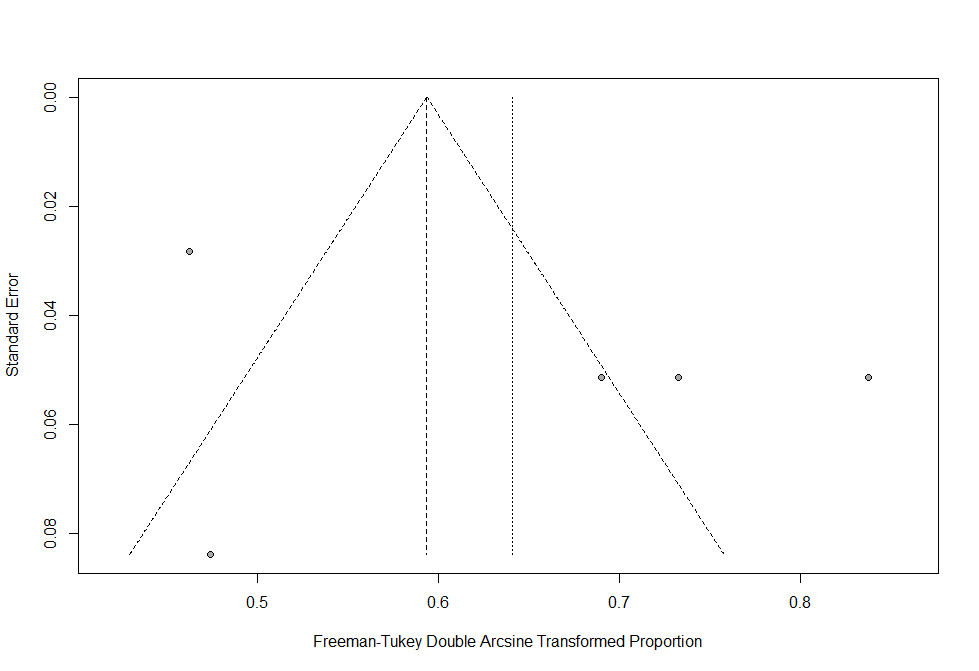


**Egger’s test:** p-value 0.32

**Figure S 15:** Funnel plot of incidence of all-grade overall potential irAEs in nivolumab and ipilimumab combination (FDA approved dose)


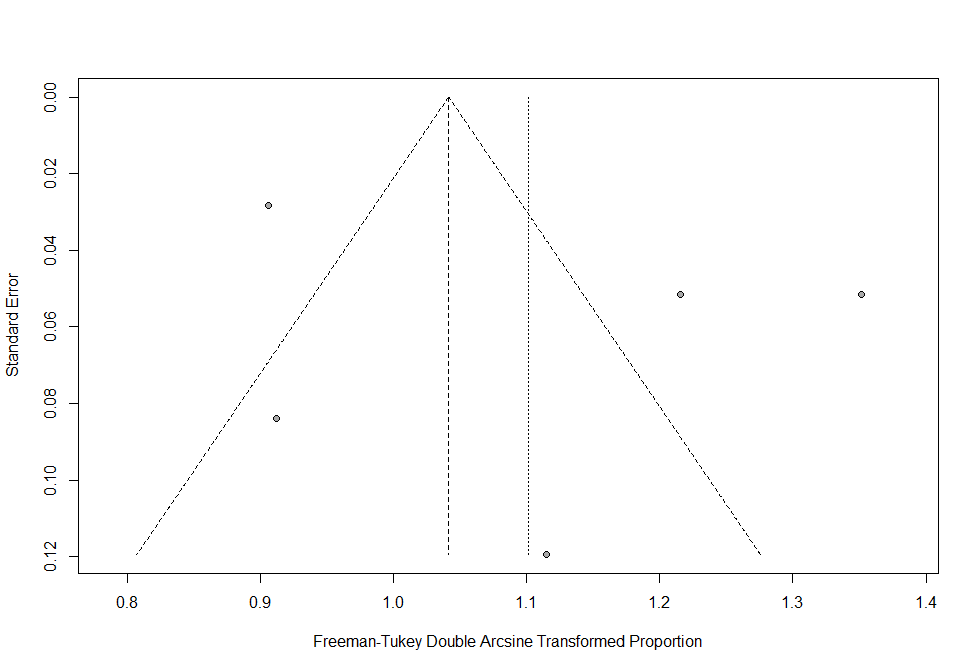


**Egger’s test:** p-value 0.46

**Figure S 16:** Funnel plot of incidence of grade≥3 overall potential irAEs in Overall nivolumab and ipilimumab combination (FDA approved dose)
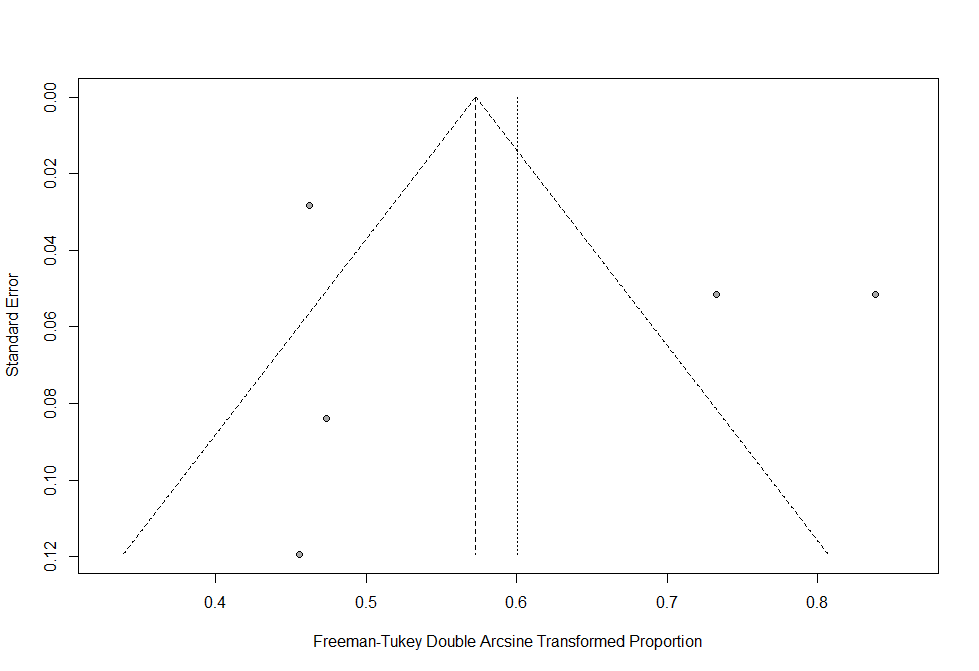


**Egger’s test:** p-value 0.62

# **Figure S 17:** Forest plot of the incidence of all-grade colitis in the sequential of ipilimumab followed by nivolumab therapy


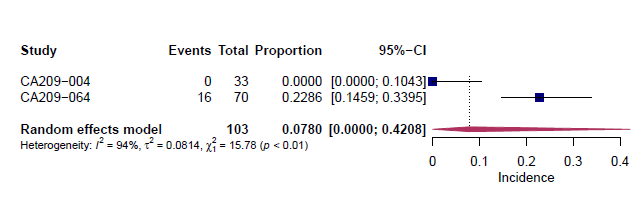


# **Figure S 18:** Forest plot of the incidence of all-grade elevated alanine aminotransferase (ALT) in the sequential of ipilimumab followed by nivolumab therapy


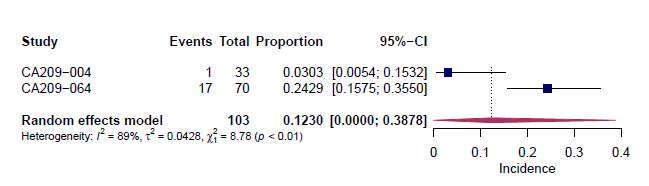


# **Figure S 19:** Forest plot of the incidence of all-grade elevated amylase in the sequential of ipilimumab followed by nivolumab therapy


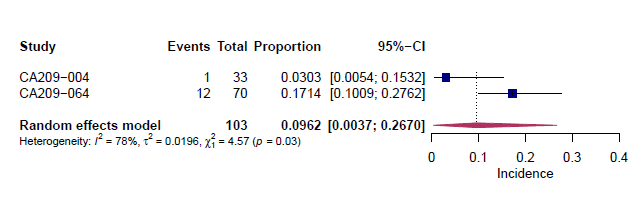


# **Figure S 20:** Forest plot of the incidence of all-grade elevated aspartate aminotransferase (AST) in the sequential of ipilimumab followed by nivolumab therapy


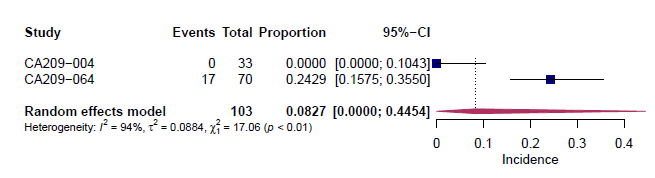


# **Figure S 21:** Forest plot of the incidence of all-grade elevated lipase in the sequential of ipilimumab followed by nivolumab therapy


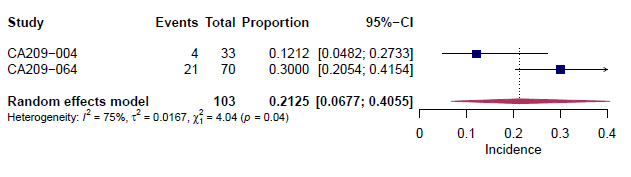


# **Figure S 22:** Forest plot of the incidence of all-grade pruritus in the sequential of ipilimumab followed by nivolumab therapy


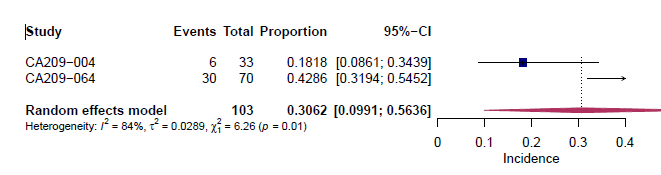


# **Figure S 23:** Forest plot of the incidence of all-grade rash in the sequential of ipilimumab followed by nivolumab therapy


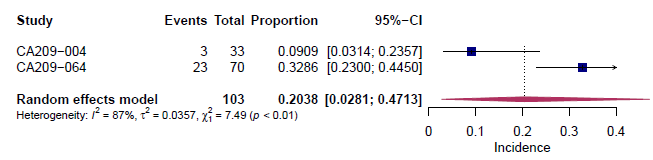


# **Figure S 24:** Forest plot of the incidence of all-grade vitiligo in the sequential of ipilimumab followed by nivolumab therapy


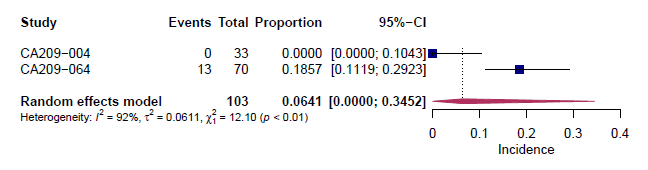


# **Figure S 25:** Forest plot of the incidence of all-grade diarrhea in the sequential of ipilimumab followed by nivolumab therapy


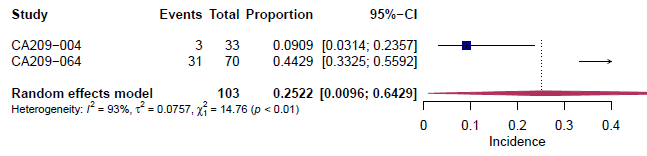


# **Figure S 26:** Forest plot of the incidence of all-grade hypothyrodism in the sequential of ipilimumab followed by nivolumab therapy


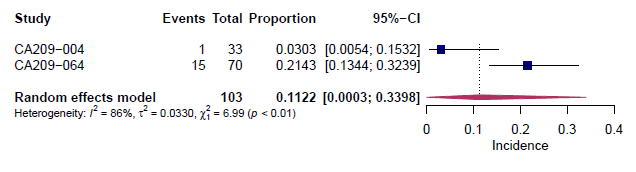


# **Figure S 27:** Forest plot of the incidence of all-grade hepatitis in nivolumab monotherapy


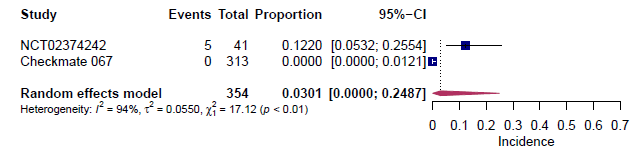


# **Figure S 28:** Forest plot of the incidence of grade≥3 hepatitis in nivolumab monotherapy


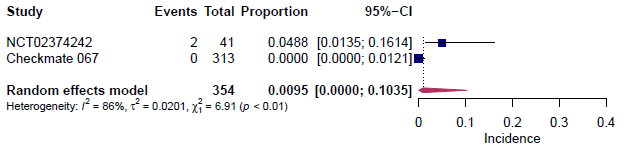


# **Figure S 29:** Forest plot of the incidence of grade≥3 lymphopenia in nivolumab monotherapy

| A: main analysis  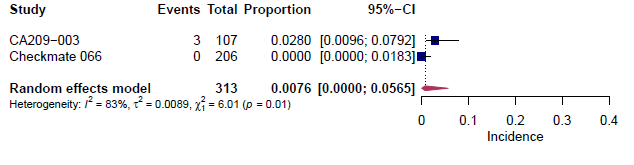 |
| --- |
| B: FDA approved dose analysis |


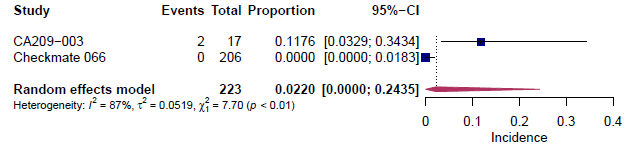


# **Figure S 30:** Forest plot of the incidence of all-grade muscle spasms in nivolumab monotherapy


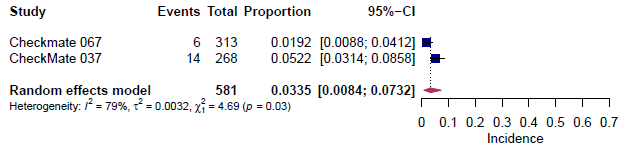


# **Figure S 31:** Forest plot of the incidence of all-grade skin hypopigmentation in ipilimumab and nivolumab concomitant therapy


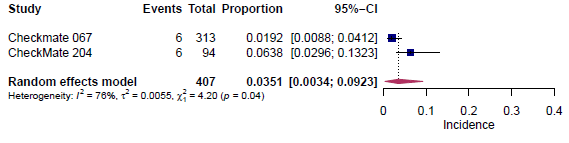


# **Figure S 32:** Forest plot of the incidence of all-grade overall incidence of irAEs in ipilimumab monotherapy by prior treatment experience

A: main analysis


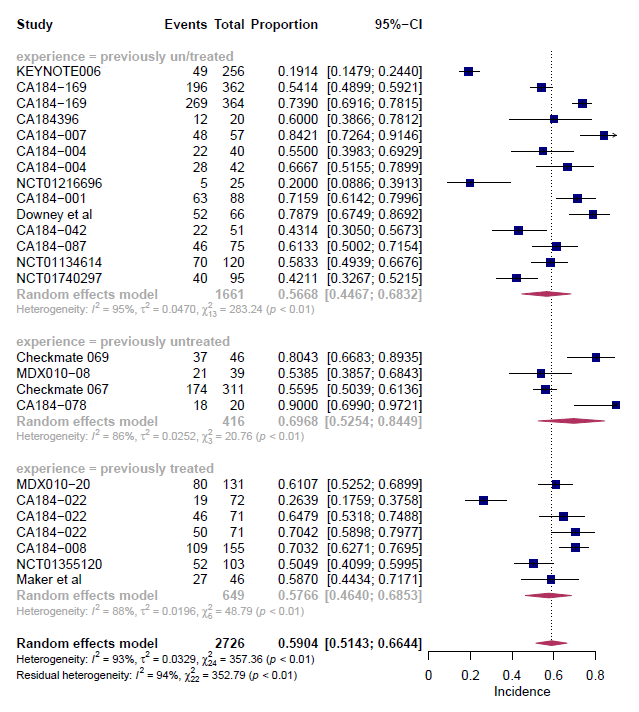


B: FDA approved dose analysis


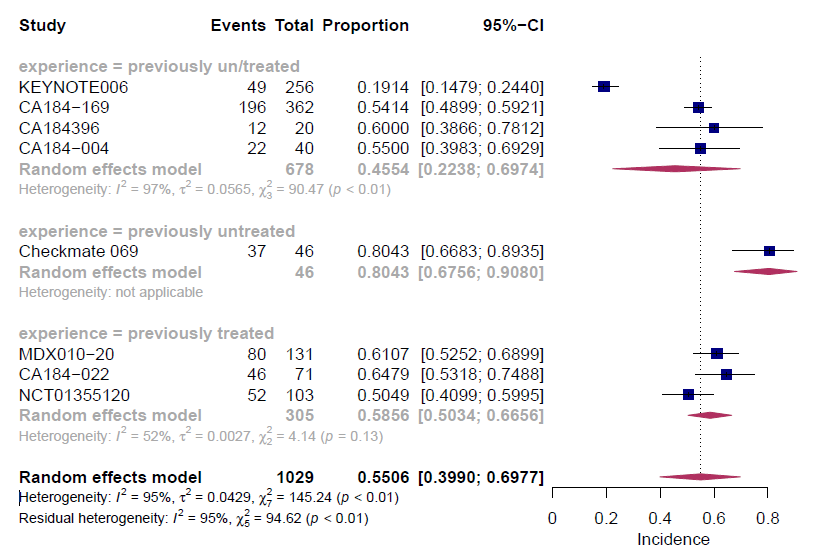


# **Figure S 33:** Forest plot of the incidence of all-grade overall incidence of irAEs in nivolumab monotherapy by prior treatment experience

A: main analysis


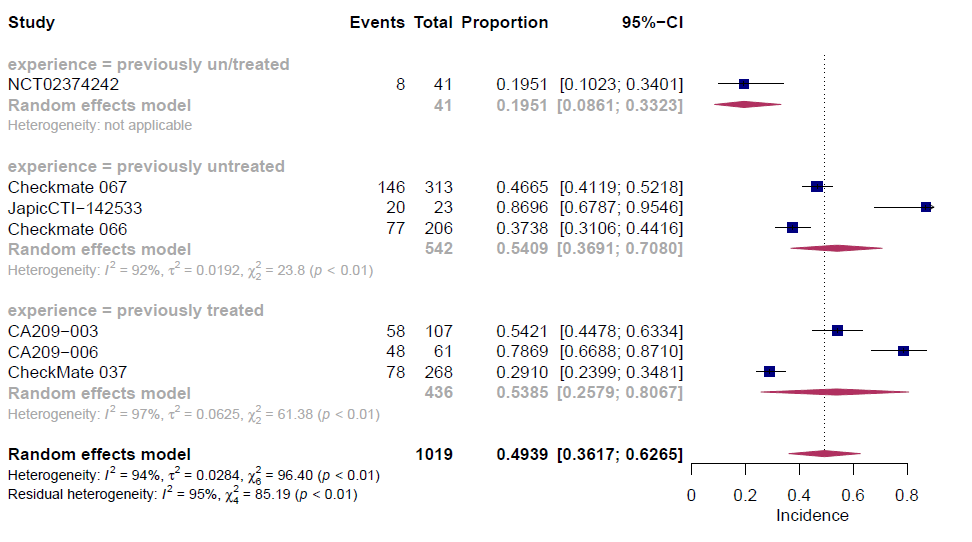


B: FDA approved dose analysis


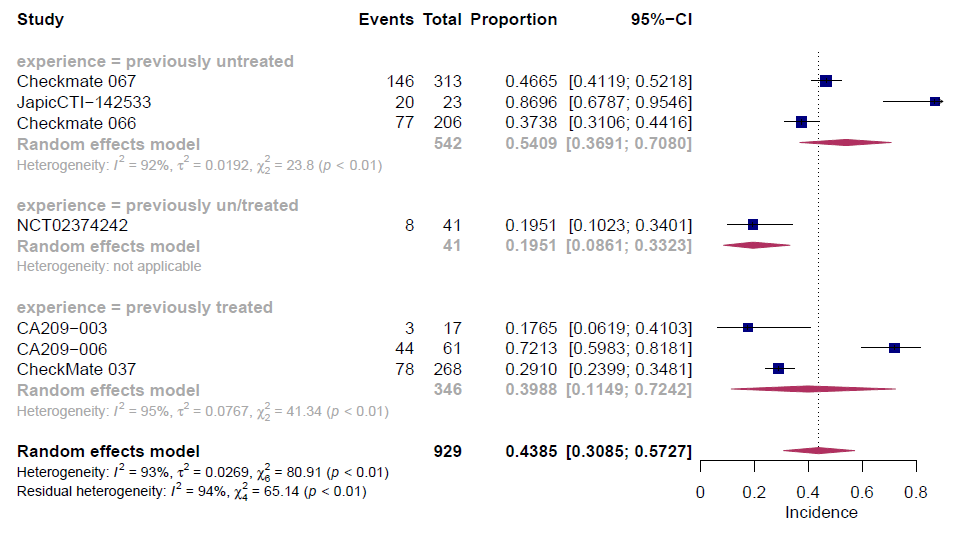


# **Figure S 34:** Forest plot of the incidence of all-grade overall incidence of irAEs in ipilimumab and nivolumab combination therapy by prior treatment experience

A: main analysis


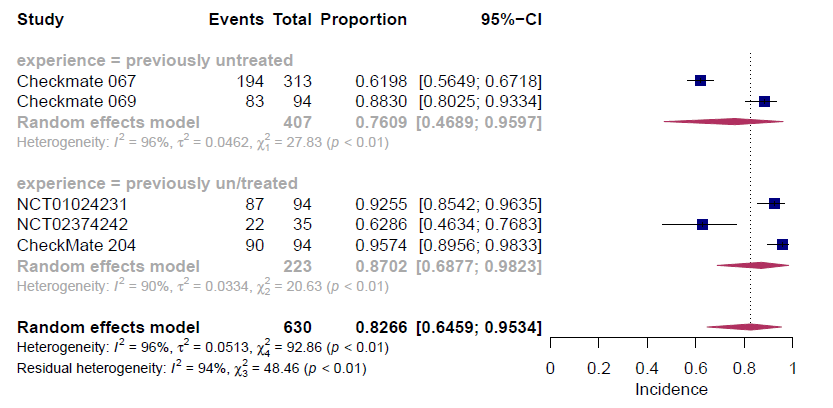


B: FDA approved dose analysis


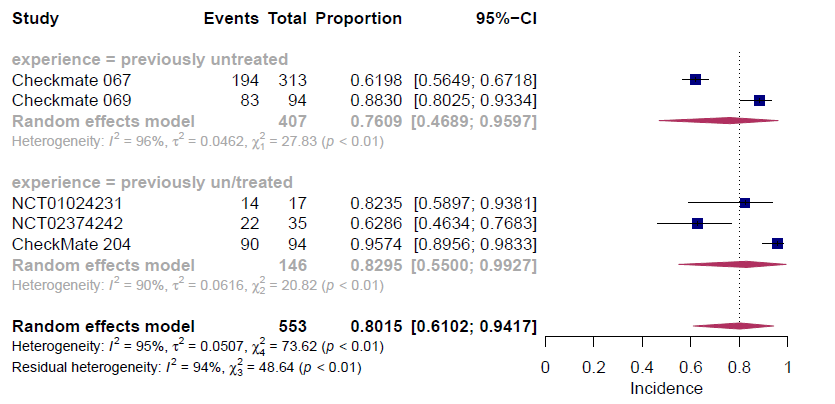


# **Figure S 35:** Forest plot of the incidence of grade≥3 overall incidence of irAEs in ipilimumab and nivolumab combination therapy by prior treatment experience

A: main analysis


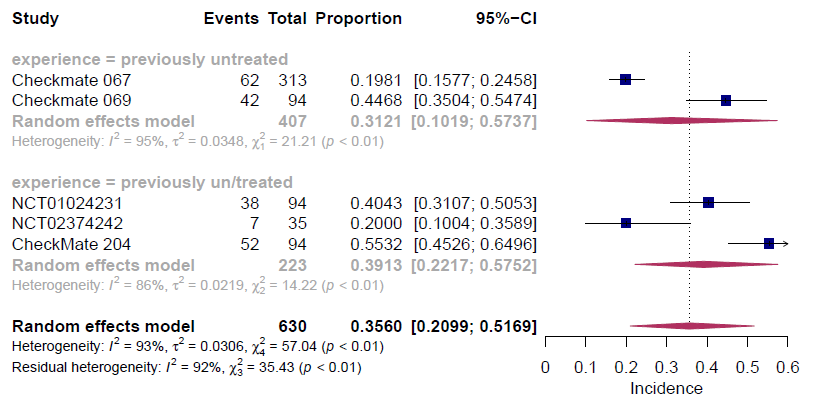


B: FDA approved dose analysis


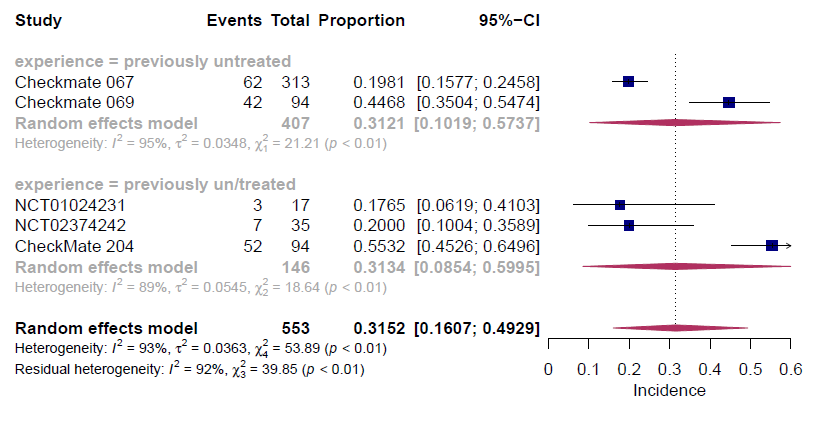


# **Figure S 36:** Forest plot of the incidence of all-grade elevated amylase in ipilimumab monotherapy by prior treatment experience

| A: main analysis  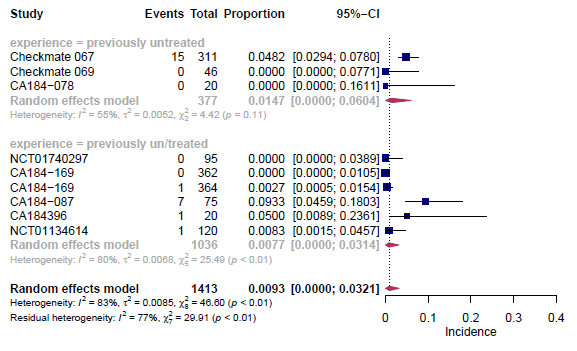 |
| --- |
| B: FDA approved dose analysis  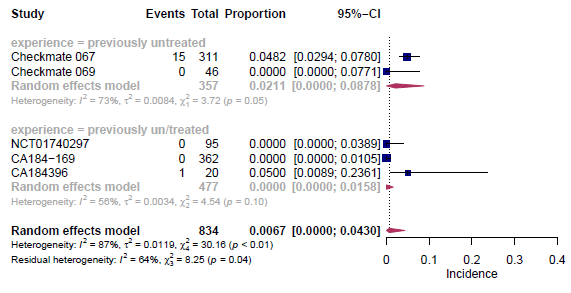 |

# **Figure S 37:** Forest plot of the incidence of all-grade arthralgia in ipilimumab monotherapy by prior treatment experience

| A: main analysis  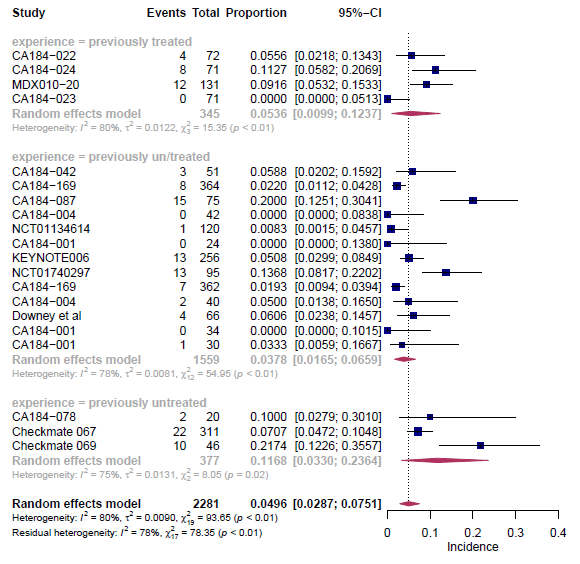 |
| --- |
| B: FDA approved dose analysis  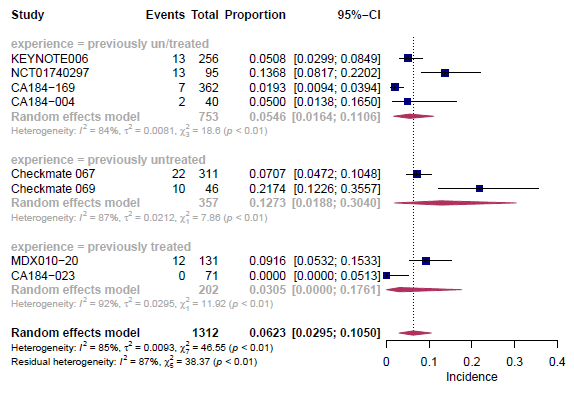 |

# **Figure S 38:** Forest plot of the incidence of all-grade dermatitis in ipilimumab monotherapy by prior treatment experience

| A: main analysis  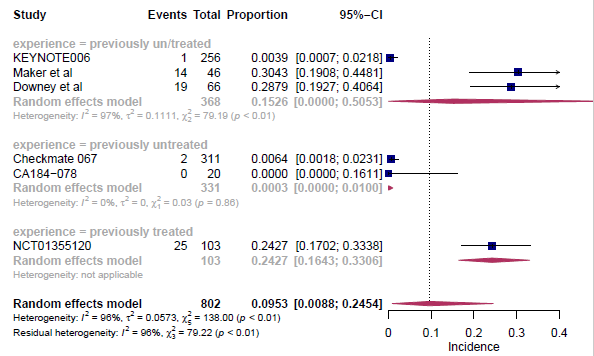 |
| --- |
| B: FDA approved dose analysis  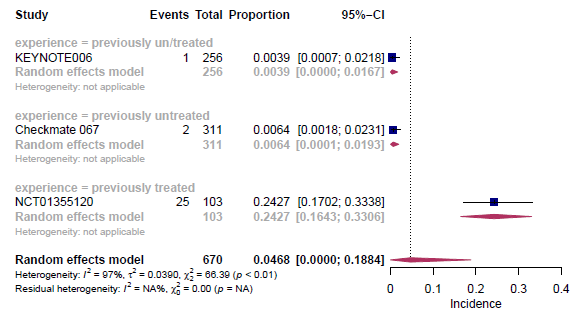 |

# **Figure S 39:** Forest plot of the incidence of all-grade hyperglycemia in ipilimumab monotherapy by prior treatment experience

| A: main analysis  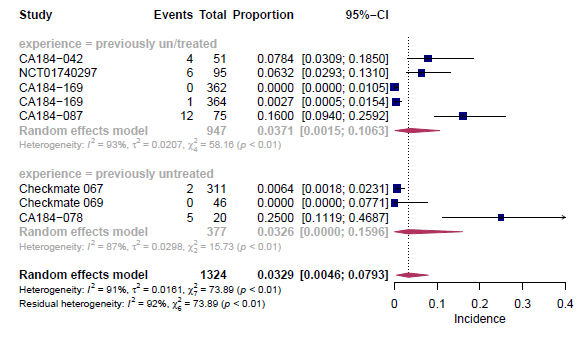 |
| --- |
| B: FDA approved dose analysis  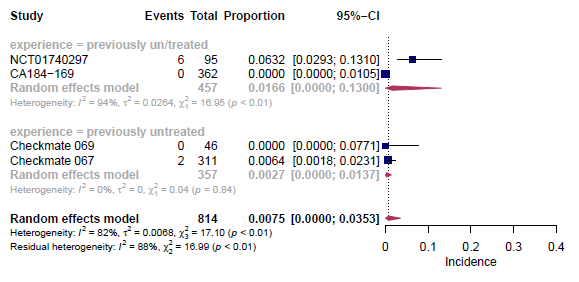 |

# **Figure S 40:** Forest plot of the incidence of all-grade elevated lipase in ipilimumab monotherapy by prior treatment experience

| A: main analysis  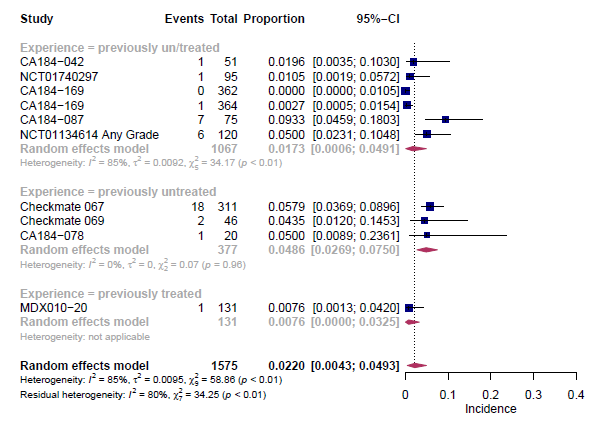 |
| --- |
| B: FDA approved dose analysis  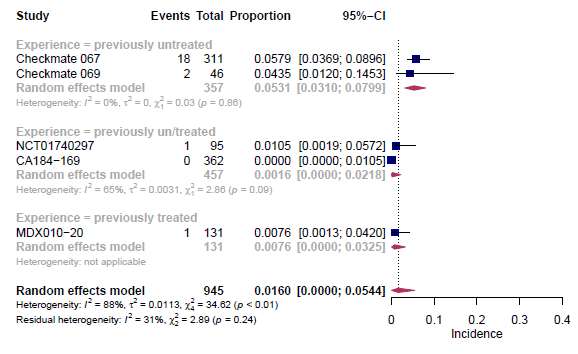 |

# **Figure S 41:** Forest plot of the incidence of grade≥3 elevated lipase in ipilimumab monotherapy by prior treatment experience

| A: main analysis  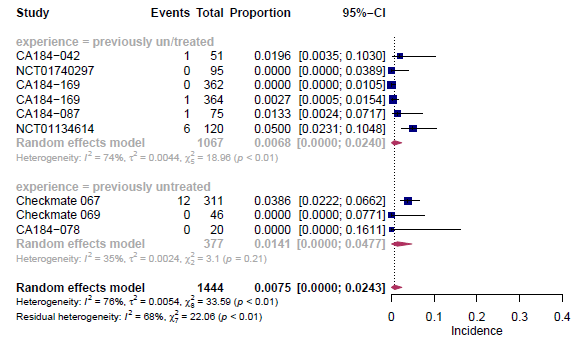 |
| --- |
| B: FDA approved dose analysis  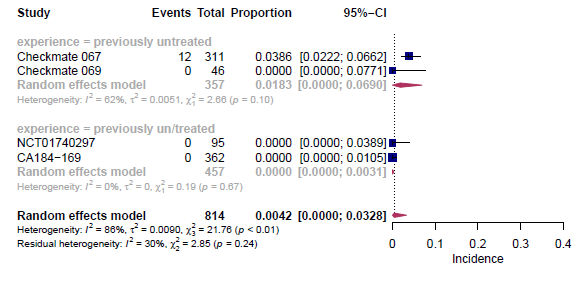 |

# **Figure S 42:** Forest plot of the incidence of all-grade pruritus in ipilimumab monotherapy by prior treatment experience

| A: main analysis  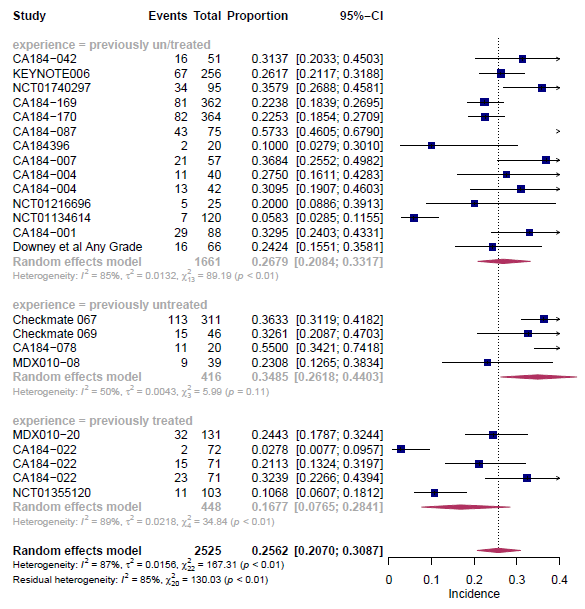 |
| --- |
| B: FDA approved dose analysis  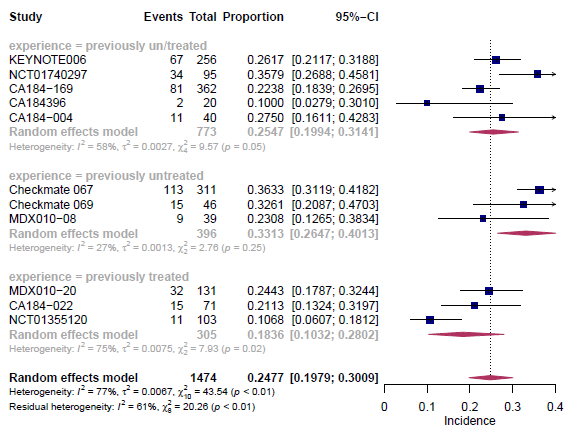 |

# **Figure S 43:** Forest plot of the incidence of all-grade rash maculopapular in ipilimumab monotherapy by prior treatment experience

| A: main analysis  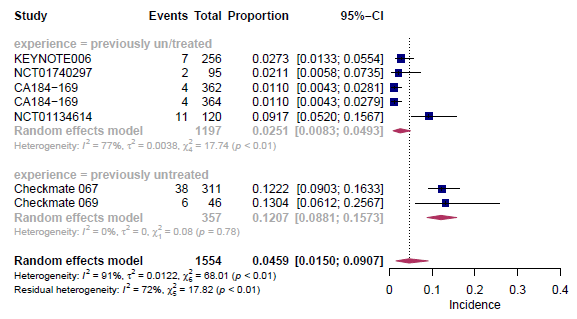 |
| --- |
| B: FDA approved dose analysis  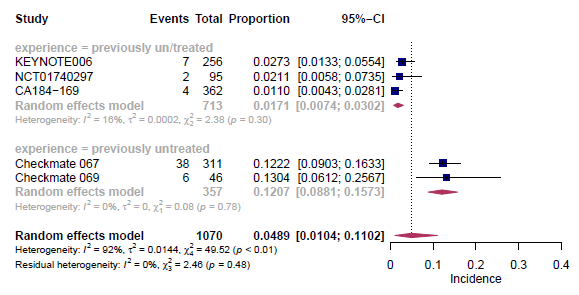 |

# **Figure S 44:** Forest plot of the incidence of all-grade elevated blood alkaline phosphatase in nivolumab monotherapy by prior treatment experience


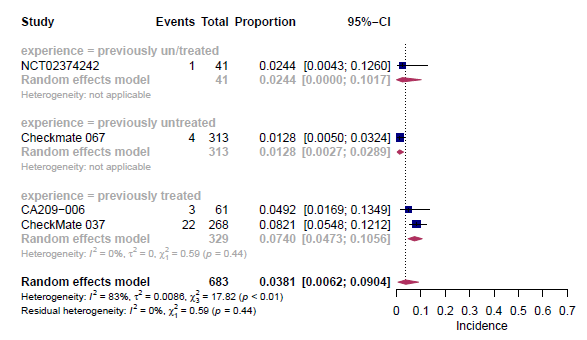


# **Figure S 45:** Forest plot of the incidence of all-grade alopecia in nivolumab monotherapy by prior treatment experience


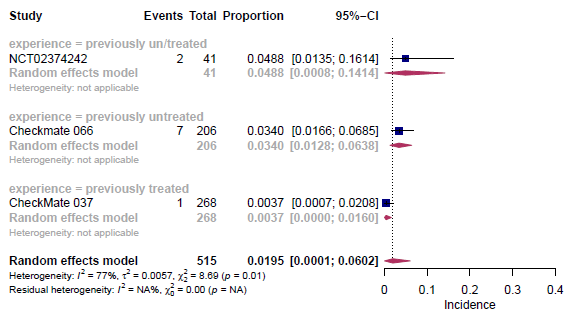


# **Figure S 46:** Forest plot of the incidence of all-grade diarrhea in nivolumab monotherapy by prior treatment experience

| A: main analysis  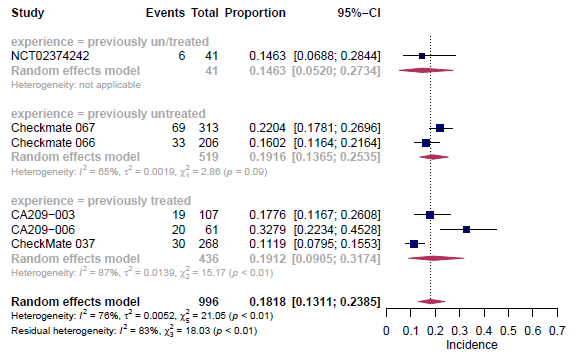 |
| --- |
| B: FDA approved dose analysis  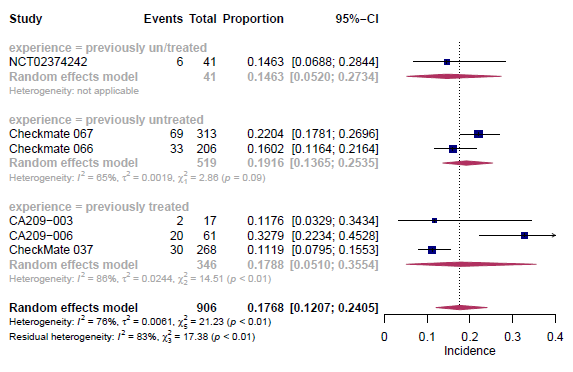 |

# **Figure S 47:** Forest plot of the incidence of all-grade elevated lipase in nivolumab monotherapy by prior treatment experience


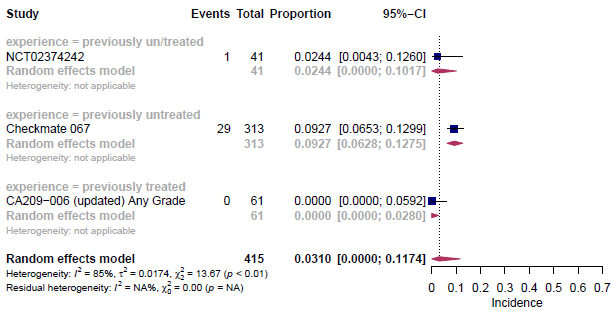


# **Figure S 48:** Forest plot of the incidence of all-grade pruritus in nivolumab monotherapy by prior treatment experience

| A: main analysis  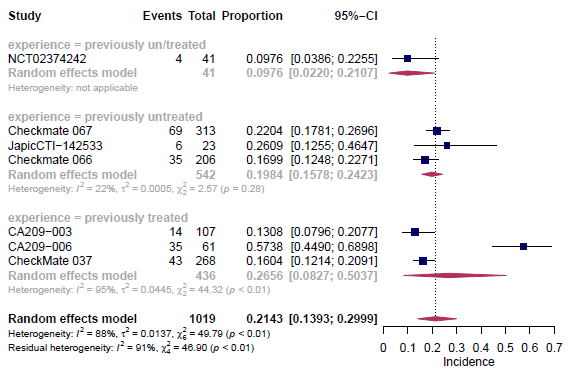 |
| --- |
| B: FDA approved dose analysis  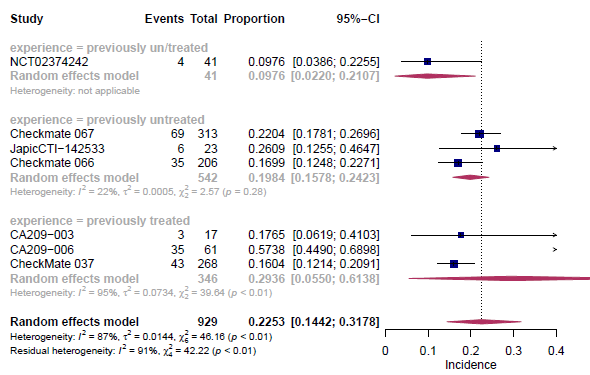 |

# **Figure S 49:** Forest plot of the incidence of all-grade rash in nivolumab monotherapy by prior treatment experience

| A: main analysis  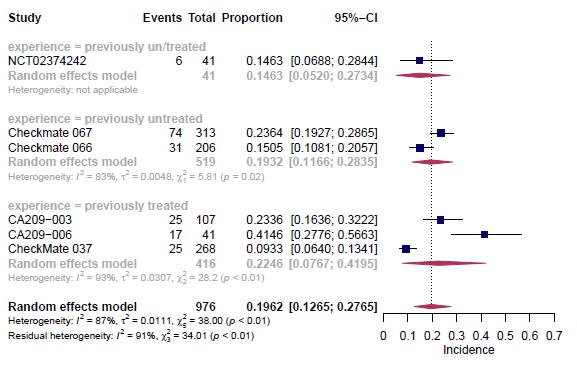 |
| --- |
| B: FDA approved dose analysis  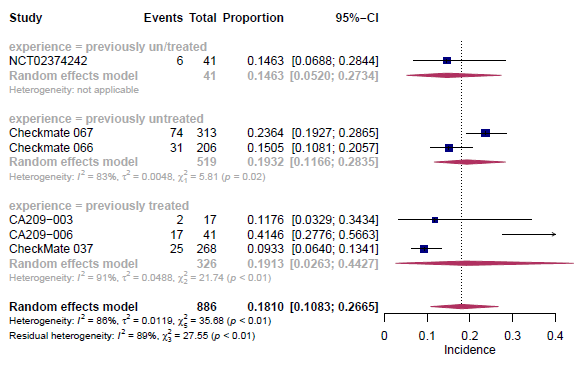 |

# **Figure S 50:** Forest plot of the incidence of all-grade maculopapular rash in nivolumab monotherapy by prior treatment experience

| A: main analysis  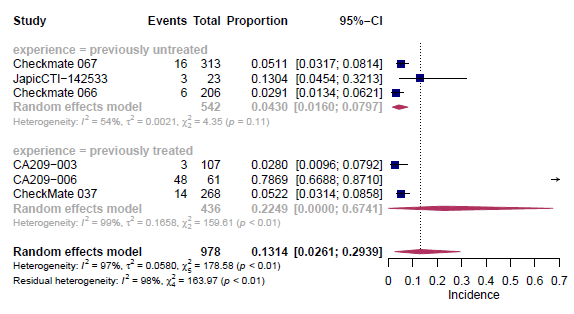 |
| --- |
| B: FDA approved dose analysis  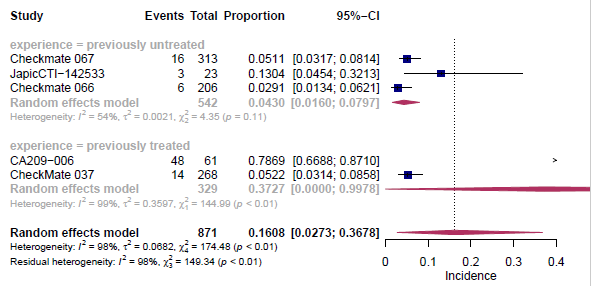 |

# **Figure S 51:** Forest plot of the incidence of all-grade elevated aspartate aminotransferase (AST) in ipilimumab and nivolumab combination therapy by prior treatment experience

| A: main analysis  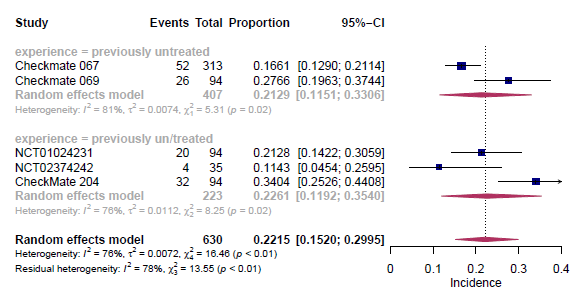 |
| --- |
| B: FDA approved dose analysis  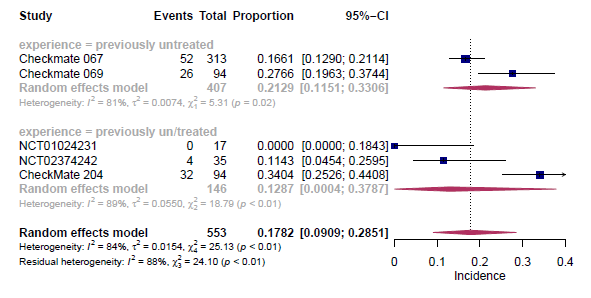 |

# **Figure S 52:** Forest plot of the incidence of all-grade hepatitis in ipilimumab and nivolumab combination therapy by prior treatment experience


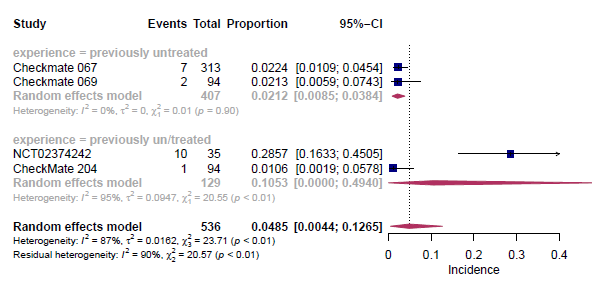


# **Figure S 53:** Forest plot of the incidence of all-grade myalgia in ipilimumab and nivolumab combination therapy by prior treatment experience


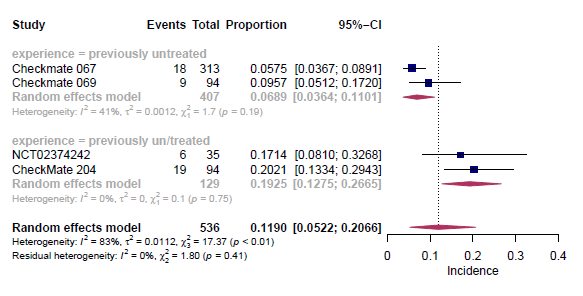


# **Figure S 54:** Forest plot of the incidence of all-grade rash in ipilimumab and nivolumab combination therapy by prior treatment experience

| A: main analysis  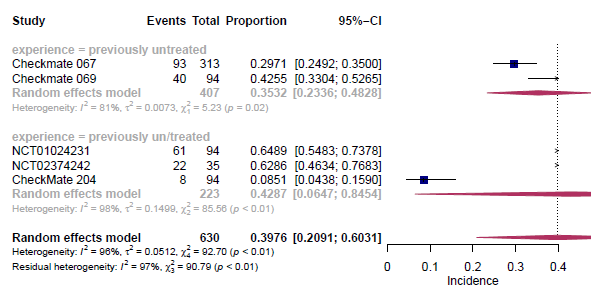 |
| --- |
| B: FDA approved dose analysis  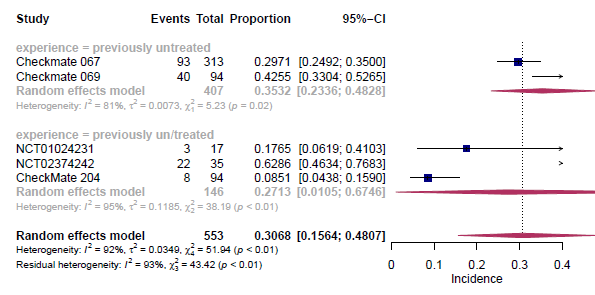 |

# **Figure S 55:** Forest plot of the incidence of all-grade maculopapular rash in ipilimumab and nivolumab combination therapy by prior treatment experience

| A: main analysis  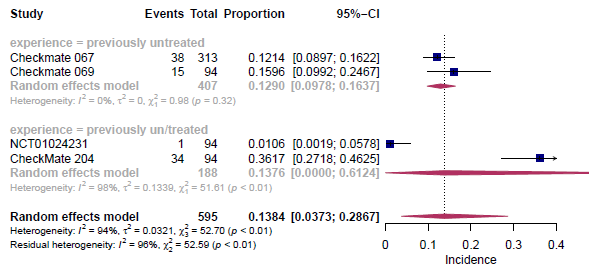 |
| --- |
| B: FDA approved dose analysis  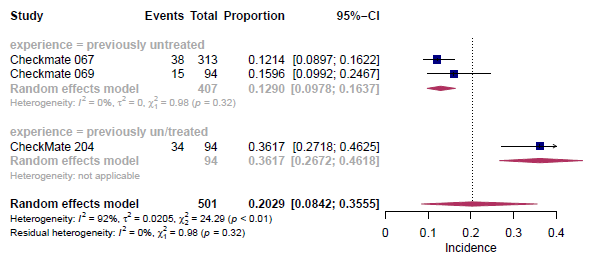 |

# **Figure S 56:** Forest plot of the incidence of grade≥3 diabetes mellites in ipilimumab (FDA dose) monotherapy by prior treatment experience


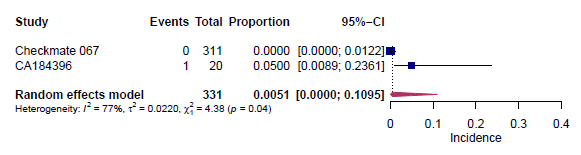


# **Figure S 57:** Forest plot of the incidence of all-grade lymphopenia in nivolumab (FDA dose) monotherapy by prior treatment experience


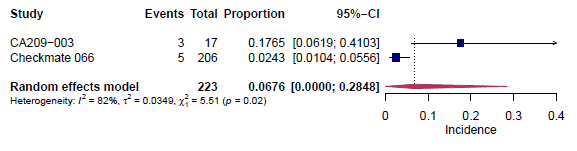


# **Figure S 58:** Forest plot of the incidence of all-grade photosensitivity in nivolumab (FDA dose) monotherapy by prior treatment experience


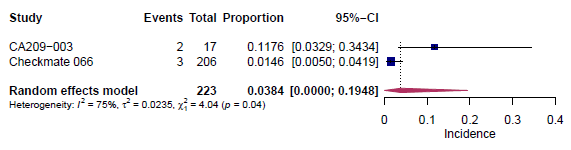


# **Figure S 59:** Forest plot of the incidence of all-grade erythema in ipilimumab and nivolumab (FDA dose) combination therapy by prior treatment experience


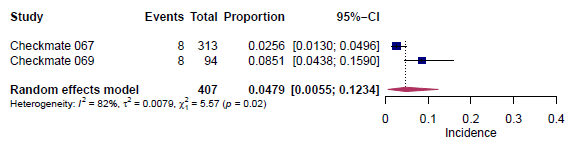


# **Figure S 60:** Forest plot of the incidence of all-grade hyperthyroidism in ipilimumab (FDA dose) monotherapy by prior treatment experience


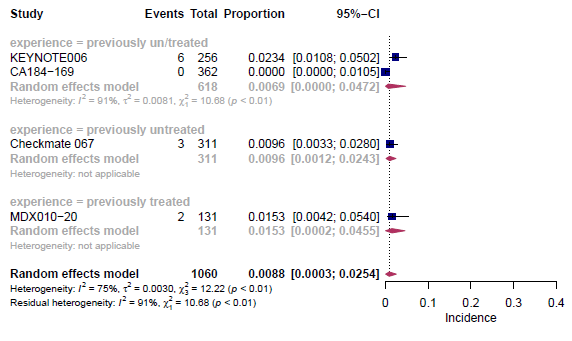


# **Figure S 61:** Forest plot of the incidence of all-grade myalgia in ipilimumab (FDA dose) monotherapy by prior treatment experience


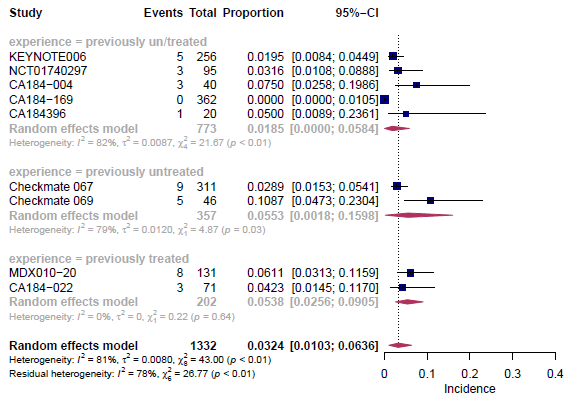


# **Figure S 62:** Forest plot of the incidence of all-grade elevated alanine aminotransferase (ALT) in ipilimumab and nivolumab (FDA dose) combination therapy by prior treatment experience


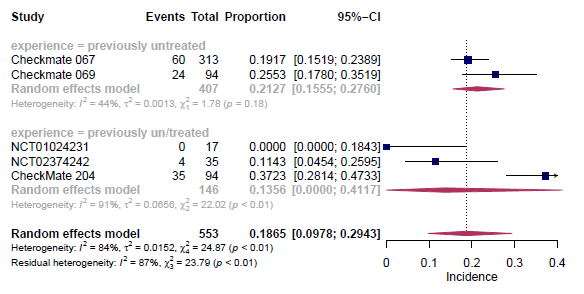

Supplement: Supplementary file 1 [file Table_1.DOCX]
